# Supplementary material for: High dose rifampin for 2 months vs standard dose rifampin for 4 months, to treat TB infection: Protocol of a 3-arm randomized trial (2R2)
Source: PLoS One. 2023 Feb 2;18(2):e0278087. doi: 10.1371/journal.pone.0278087 (PMC9894386; doi:10.1371/journal.pone.0278087)
Supplement: S1 File — (DOCX) [file pone.0278087.s002.docx]

**2R^2^**: **Higher dose Rifampin for 2 months vs Standard dose Rifampin for Latent TB:**

**a 3-arm randomized trial**

<space for signature and date>

Protocol **Version: 4**

Date**: 20 Sep 2021**

Study Principal Investigator and person authorized to sign the protocol:

Dr. Dick Menzies

Respiratory Epidemiology and Clinical Research Unit,

Montreal Chest Institute & McGill International TB Centre

5252 de Maisonneuve West, Room 3D.58

Montreal, Quebec, Canada H4A 3S5

514-934-1934 ext 32128

Names and title of Qualified Investigators* (QI) in each site:

| **Study sites** | **Qualified Investigators** |
| --- | --- |
| McGill University Health Centre (MUHC), Montreal, Canada-Montreal Chest Institute, 1001 Decarie Blvd, Montreal, QC, H4A 3J1  tel: (514) 934-1934 ext. 37217 | Dr. Dick Menzies  5252 de Maisonneuve West, Room 3D.57  Montreal, Quebec, Canada H4A 3S5  514-934-1934 ext 32128 |
| Montreal Children’s Hospital, Respiratory Service. 1001 Decarie Blvd, Montreal, QC, H4A 3J1. S1.3333 Tel: [(514) 412-4400](https://www.google.com/search?client=firefox-b-d&channel=crow&ei=u2ZMXc3sL8m0ggeLnrmYBg&q=children+hospital++GLEN&oq=children+hospital++GLEN&gs_l=psy-ab.3..0i7i30l5j0i30l5.15649.24605..24889...0.0..0.148.776.8j1......0....1..gws-wiz.......0i8i7i30.JHIl8r7PTAw&ved=&uact=5) ext. 22843 | Dr. David Zielinski, Montreal Children’s Hospital, Respiratory Service. 1001 Decarie Blvd, Montreal, QC, H4A  514-412-4400 ext. 24444 |
| BC Centre for Disease Control – TB Clinic 655 West 12th Avenue Vancouver BC V5Z 4R4  [(604) 707-2400](https://www.google.com/search?rlz=1C1CAFA_enCA705CA705&ei=3dVeXcjLHemOggfk_5CABA&q=BC+Centre+for+Disease+Control+%E2%80%93+TB+Clinic+655+West+12th+Avenue+Vancouver+BC+V5Z+4R4&oq=BC+Centre+for+Disease+Control+%E2%80%93+TB+Clinic+655+West+12th+Avenue+Vancouver+BC+V5Z+4R4&gs_l=psy-ab.3...19934.19934..20875...0.0..0.72.72.1......0....2j1..gws-wiz.18FCQIUYOGw&ved=0ahUKEwiIrsuWhZfkAhVph-AKHeQ_BEAQ4dUDCAo&uact=5)  and  New Westminster clinic 100 – 237 Columbia Street E New Westminster, BC V3L 3W4  [(604) 707-2698](https://www.google.com/search?q=New+Westminster+clinic+100+%E2%80%93+237+Columbia+Street+E+New+Westminster%2C+BC+V3L+3W4&rlz=1C1CAFA_enCA705CA705&oq=New+Westminster+clinic+100+%E2%80%93+237+Columbia+Street+E+New+Westminster%2C+BC+V3L+3W4&aqs=chrome..69i57.1213j0j8&sourceid=chrome&ie=UTF-8) | Dr. James Johnston;  BC Centre for Disease Control  c/o 1073B 655 West 12th Avenue Vancouver BC V5Z 4R4  [james.johnston@bccdc.ca](mailto:james.johnston@bccdc.ca)  604-707-5662 |
| Tuberculosis Services  106-2675 36th Street NE, Calgary, AB, T1Y 6H6,  Canada  Tel: [(403) 944-7660](https://www.google.com/search?q=Tuberculosis+Services+106-2675+36th+Street+NE%2C+Calgary%2C+AB%2C+T1Y+6H6&rlz=1C1CAFA_enCA705CA705&oq=Tuberculosis+Services+106-2675+36th+Street+NE%2C+Calgary%2C+AB%2C+T1Y+6H6&aqs=chrome..69i57.1743j0j8&sourceid=chrome&ie=UTF-8) | Dr. Dina Fisher;  3383 26th Ave NE, Calgary, T1Y 6L4  [Dina.Fisher@albertahealthservices.ca](mailto:Dina.Fisher@albertahealthservices.ca)  403-943-4971 |
| Edmonton Tuberculosis Clinic  Aberhart Centre One  11402 University Avenue, Room 9232  Edmonton, Alberta  T6G 2J3  [(780) 407-4550](https://www.google.com/search?q=Edmonton+Tuberculosis+Clinic+Aberhart+Centre+One+11402+University+Avenue%2C+Room+9232+Edmonton%2C+Alberta+T6G+2J3&rlz=1C1CAFA_enCA705CA705&oq=Edmonton+Tuberculosis+Clinic+Aberhart+Centre+One+11402+University+Avenue%2C+Room+9232+Edmonton%2C+Alberta+T6G+2J3&aqs=chrome..69i57.2070j0j7&sourceid=chrome&ie=UTF-8) | Dr. Richard Long;  3-04 South Academic Building (SAB)  University of Alberta,Edmonton, Alberta  Canada T6G 2G7  tel. (780) 407-1427  [rlong@ualberta.ca](mailto:rlong@ualberta.ca) |
| Klinik Penelitian Tuberculosis (TB research clinic)  Eijkman Building, first floor  Bandung, West Java, 40161  Indonesia | Dr. Rovina Ruslami;  Faculty of Medicine, UNPAD  Jl. Prof. Eijckman no. 38  Bandung, West Java, 40161  Indonesia +62 22 779 5594  [n.ruslami@gmail.com](mailto:n.ruslami@gmail.com) |
| Ho Chi Minh City, Vietnam;  Nam Tu Liem District Health Center, No. 3 Lien Co, Cau Dien, Nam Tu Liem District, Hanoi; Tel: +84 (024)37680005 ;  Hai Ba Trung District Health Center, No. 16B, Pham Dinh Ho Street, Hai Ba Trung District, Hanoi, Tel: +84 (024)39713204 ;  Hanoi Lung Hospital, No. 44, Thanh Nhan Street, Hai Ba Trung District, Hanoi, Tel: +84 (024)38210975 ;  Phoi Viet TB and Respiratory Diseases Clinic , No. 20-22 Ngo Quyen, Ward 5, District 10, Ho Chi Minh City, Tel: +84 (028)39575099 | Dr. Nguyen Viet Nhung - National TB Program, 463 Hoang Hoa Tham, Badinh, Hanoi,Viet Nam. Tel: (+84) 4 3832 6002.  Dr. Greg Fox - University of Sydney, NSW, Australia. Woolcock Institute of Medical Research  431 Glebe Point Road, Glebe NSW 2037  [greg.fox@sydney.edu.au](mailto:greg.fox@sydney.edu.au)  +61 29 114 0000 |

*Note : “Qualified Investigator” is equivalent to site “Principal Investigator”.

Dr. Menzies , all site Qualified Investigators, and the members of the DSMB are also the medical experts and the qualified physicians who will be responsible for all trial related medical decisions.

Hypothesis: 4

Objectives 4

Primary objectives 4

Secondary objectives 4

Background 5

Importance of latent tuberculosis infection 5

Currently recommended regimens for treatment of latent TB 5

High dose rifampin and shorter treatment for latent tuberculosis infection: 6

Evidence for high dose rifampin in treatment of active disease 6

Evidence for high dose rifamycins to shorten treatment of active TB 9

Evidence of use of high dose rifamycins in short latent TB treatment regimens 10

Summary: Use of high dose rifampin for treatment of latent TB 10

Design 12

Rationale: 12

Outcomes 13

Primary outcomes 13

Secondary outcomes 14

Trial procedures 14

Intervention arms: 14

Comparator: 14

Accountability procedures for investigational and comparator drugs: 15

Concomitant treatments 15

Population and eligibility criteria: 16

Inclusion criteria: 16

Exclusion criteria: 17

Rationale for exclusion of children <10 years old and inclusion of children aged 10-17: 18

Sample size considerations 19

Statistical analysis 21

Randomization 24

Data gathering: 24

Quality management: 24

Pre-treatment: 25

During treatment 26

Post-treatment 27

Pharmaco-kinetics (PK) studies 27

Enrolment and follow-up during COVID-19 related measures. 28

Methods to ensure the safety of study participants: 29

Adjudication panels for secondary outcome ascertainment: 32

Ethical considerations: 32

Study committees 33

References 34

Appendix 1: Potentially important drug interactions for rifampin. 40

Appendix 2: 2R2 Supplement document: Safety and efficacy of LTBI treatment 46

**List of abbreviations:**

2R_20_ 2 months of rifampin at 20mg/kg/day

2R_30_ 2 months of rifampin at 30mg/kg/day

4R_10_ 4 months of rifampin at 10mg/kg/day

H: isoniazid

IGRA: Interferon Gamma Release Assays

INH: isoniazid

LTBI: Latent Tuberculosis Infection

R: rifampin

RIF: rifampin

TB: tuberculosis

TST: tuberculin skin test

WHO: World Health Organization

# Hypothesis:

Two months of daily self-administered rifampin (R) at double or triple the standard dose will have superior completion and non-inferior safety compared to 4-months daily self-administered rifampin at the standard dose for persons aged 10 and older who are recommended by their health care providers to take treatment for latent TB infection.

# Objectives

### Primary objectives

1) Compare completion of three different regimens of daily self-administered oral rifampin: (1) Standard = 10 mg/kg/day for 4 months (4R_10_), or (2) 20 mg/kg/day for 2 months (2R_20_), or, (3) 30 mg/kg/day for 2 months (2R_30_). Completion will be defined as taking at least 80% of doses in 120% of allowed time (48 doses within 72 days for the 2-month high dose regimens and 96 doses within 144 days for the standard regimen).

2) Compare the three regimens in terms of their rate of Grade 3 to 5 adverse events (AE) that result in permanent discontinuation of study drug and are considered probably or possibly related to the study drug by an independent 3-member adjudication panel blinded to study arm (safety).

### Secondary objectives

1. Compare the 3 regimens’ rates of grade 1 to 2 adverse events that result in permanent discontinuation of study drug and are considered probably or possibly related to the study drug by the same independent and blinded adjudication panel (safety).
2. Compare the three regimens in terms of rates of active TB within 26 months of randomization (efficacy).

# Background

## Importance of latent tuberculosis infection

It has been estimated that approximately one quarter of the entire world’s population carry latent tuberculosis infection (LTBI). Of these, approximately 10% will develop disease, meaning that about 200 million persons will develop active disease out of the current vast pool with latent infection. In the plan of the World Health Organization (WHO) to “end TB”, the treatment of latent TB is key to achieve the goal of TB elimination by 2035 (Uplekar et al. 2015). Currently treatment of LTBI is recommended by WHO to all close contacts, persons living with HIV, and persons with other immune-suppressing conditions (World Health Organization 2018a). A specific goal mentioned at the United Nations General Assembly on Tuberculosis held in September 2018 was that 30 million persons with latent TB infection must be diagnosed and treated in the next 5 years (World Health Organization 2018b).

A major limitation of current latent TB management is that the available diagnostic tools – the tuberculin skin test (TST) and the Interferon Gamma Release Assays (IGRA) - only identify individuals with an immune response to tuberculosis antigens. Of these, approximately 10% will develop active disease over their lifetime. However neither the TST nor the IGRA identify which of those with latent TB will develop disease. Hence, treatment must be offered to 10 persons to prevent disease in one – a very inefficient approach.

Given this, it is imperative that treatment of latent TB infection should be short, inexpensive and non-toxic. However, such a treatment (i.e. short, inexpensive and not-toxic) is not yet available. The treatment recommended by the World Health Organization (WHO), and the current standard regimen in the great majority of countries world-wide, is 6 months of INH (6H). Although multiple randomized trials (Comstock, Hammes, and Pio 1969; Gordin et al. 1997; Hawken et al. 1997; International Union Against Tuberculosis Committee on Prophylaxis 1982) and meta-analyses (Smieja MJ et al. 2000; Zenner et al. 2017) have established that this regimen does prevent TB, the efficacy in placebo-controlled trials of 6H is estimated to be only 70% (International Union Against Tuberculosis Committee on Prophylaxis 1982). In addition to sub-optimal efficacy, (and obviously not being short), 6H also can cause significant liver toxicity which may be fatal in rare circumstances. Hence, development of shorter regimens for latent TB, which are effective to prevent TB and safer is an urgent priority.

## Currently recommended regimens for treatment of latent TB

In January 2018 the WHO published updated recommendations for treatment of LTBI (World Health Organization 2018a). In LMIC three regimens are recommended as equivalent options: 6 months of isoniazid (6H), isoniazid and rifampin for 3-4 months (3-4HR), and once-weekly (1/7) isoniazid plus rifapentine for 3 months (3HP). The latter two, rifamycin containing regimens have been shown to have superior completion, and non-inferior efficacy compared to 6-9 months INH in randomized trials (Geijo et al. 2007; Hong Kong Chest Service Tuberculosis Research Centre 1992; Spyridis NP et al. 2007), and meta-analyses (Njie et al. 2018; Pease et al. 2017; Zenner et al. 2017) . However both of these shorter regimens have had similar (3HR) or significantly higher (3HP) rates of Grade 3-4 adverse events than the longer mono-INH. Hence safety remains an issue. WHO recommended 4R as a 4^th^ option for countries with TB disease incidence less than 100/100,000. In an earlier placebo-controlled trial, 3 months daily rifampin (3R) had estimated efficacy of 63% (Hong Kong Chest Service Tuberculosis Research Centre 1992), which was not significantly different from the efficacy from that of 6H, which has an estimated efficacy of 69% in earlier trials (International Union Against Tuberculosis Committee on Prophylaxis 1982). In our recently completed randomized trials in 6800 adults and children, the 4R regimen was virtually identical to 9H for prevention of active TB (Diallo et al. 2018; Menzies et al. 2018) and 9H has been estimated to have 90% efficacy for TB prevention (Comstock 1999). This evidence supported the recommendation of using 4 months as minimum duration for mono-rifampin therapy at standard doses (Canadian Thoracic Society 2014) (American Thoracic Society 2000)).

Treatment with 4R was found to have superior completion in children and adults, and significantly superior safety in adults, without any serious adverse events in children (Diallo et al. 2018; Menzies et al. 2018). Unfortunately, these 4 regimens have not been compared head-to-head in trials, although a recent network meta-analysis found that 4R was the safest and most effective regimen of the four options (Zenner et al. 2017). All of these ‘short’ regimens have lower health system costs (Aspler A et al. 2010; Doan et al. 2019), largely related to the reduced number of follow-up visits required. Fewer follow-up visits should result in reduced burden on patients as well. As a result of these trials, and the meta-analysis, 4R and 3HP will become the two preferred regimens for LTBI treatment in forthcoming recommendations by CDC/NTBCA (still confidential), and the Canadian Thoracic Society will likely issue similar recommendations in 2019 (study PI, Dr. Menzies, is involved in writing these Standards).

Although the change from 6-9 months INH to the shorter rifamycin-based regimens should result in a significant improvement in LTBI treatment completion, these new “short” regimens are still 3-4 months in duration. This remains challenging as they still result in substantial burden of time and costs – for patients and health system. A reduction in duration to two months would represent substantially less burden for patients and would significantly enhance the feasibility for health system in low and middle income countries of large scale implementation of latent TB treatment – felt to be needed to achieve sustained improvement in the global TB epidemic (World Health Organization 2018b, 2015).

## High dose rifampin and shorter treatment for latent tuberculosis infection:

### Evidence for high dose rifampin in treatment of active disease

The current dose of rifampin for treatment of active disease and latent infection is 10mg/kg per day with a maximum of 600 mg per day. This dose was established in multiple randomized trials conducted in the 1960s and 1970s. In retrospect, the dose selected was the lowest possible effective dose - to minimize adverse events and costs, as rifampin was very expensive at that time, and safety was uncertain. As shown in table 1 (attached), several studies in the 1960s and 1970s assessed higher doses of rifampin, although in most of these studies the higher doses of rifampin were given once or twice per week. In these studies once-weekly rifampin was associated with unacceptably high toxicity, primarily due to systematic drug reactions (a “flu-like syndrome”). The once-weekly high dose regimens were abandoned. The twice weekly high dose achieved similar efficacy as daily standard doses, and interest in high dose rifampin faded.

**TABLE 1: Incidence of serious adverse events with higher doses of rifampin for treatment of active TB**.

(Most studies enrolled patients with Pulmonary TB. Studies that enrolled patients with TB meningitis – marked with **) - **Abbreviations**: AE: Adverse events; SDR: systemic drug reaction; Hep: hepatotoxicity.

| **Author**  **(Year)** | **Rifampin Doses: Total in mg/day (mg/kg/day) Days/week** | **Duration of high doses** | **AE Definition** | **N Treated** | **Overall AE (N, %)** | **Hepatic AE (N, %)** | **SDR (flu-like) AE (N, %)** |
| --- | --- | --- | --- | --- | --- | --- | --- |
| Decroix (1969)(Decroix et al. 1969) | 900 (15) 2/7  600 (10) 7/7 | 6 mos | ns  ns | 32  39 | 0 (-)  3 (8%) | 0 (-)  2 (5%) | NR  NR |
| Long (1979)(Long, Snider, and Farer 1979) | 750 (12.5) 7/7  600 (10) 7/7 | 6 mos | ns  ns | 331  324 | 13 (4%)  13 (4%) | 5 (2%)  5 (2%) | NR  NR |
| Anastasatu (1973)(Anastasatu, Bungeteanu, and Sibila 1973) | 1200 (15) 2/7  900 (10) 2/7 | 6 mos | ns  ns | 53  49 | 1 (2%)  3 (6%) | NR  NR | NR  NR |
| Nitti (1973)(Nitti 1973) | 900 (15) 2/7  600 (10) 7/7 | 6 mos | ns  ns | 22  23 | 0 (-)  2 (9%) | 0 (-)  0 (-) | 0 (-)  1 (4%) |
| Decroix (1974)(Decroix, Sors, and Pujet 1974) | 900 (15) 2/7  600 (10) 7/7 | 6 mos | ns  ns | 53  49 | 1 (2%)  3 (6%) | 1 (2%)  3 (6%) | NR  NR |
| Singapore TB Service (1975)(Singapore Tuberculosis Service British Medical Research Council 1975) | 900 (15) 2/7  600 (10) 2/7 | 6 mos | ns  ns | 115  119 | 13 (11%)  8 (7%) | 1 (1%)  0 (-) | 9 (8%)  5 (4%) |
| Ruslami (2007)(Ruslami et al. 2007) | 600 (13.5) 7/7  450 (10) 7/7 | 2 weeks | ns  ns | 23  24 | 1 (4%)  3 (13%) | 1 (4%)  3 (13%) | 0 (-)  0 (-) |
| Ruslami **(2013)(Ruslami et al. 2013) | 600 (13) 7/7  450 (10) 7/7 | 2 weeks | Grade 3-4 | 29  31 | 7 (24%)  5 (16%) | 6 (21%)  5 (16%) | 0 (0%)  0 (0%) |
| Yunivita **(2016)(Yunivita et al. 2016) | 750 (17) 7/7  900 (20) 7/7  600 (13) 7/7 | 2 weeks | All grades;  grade 4 Hep | 11  9  10 | 7 (64%)  7(78%)  4 (40%) | 0  1 (11%)  0 | 0(0%)  0(0%)  0(0%) |
| Heemskerk**(2016) (Heemskerk et al. 2016) | 900 (15) 7/7  600 (10) 7/7 | 8 weeks | All grades | 408  409 | 240 (59%)  229(56%) | 17(4%)  28 (7%) | NR |
| Jindani(2016)(Jindani et al. 2016) | 600(10) 7/7  900(15) 7/7  1200 (20) 7/7 | 16 weeks | Grade 3 hep, all AE | 100  100  100 | 20 (20%)  22(22%)  13 (13%) | 1 (1%)  2 (2%)  4 (4%) | NR |
| Aarnoutse (2017)(Aarnoutse et al. 2017) | 1200 (20) 7/7  900 (15) 7/7  600 (10) 7/7 | 2 weeks | Grade 3-5 CTC criteria | 50  50  50 | 5 (10%)  1 (2%)  5 (10%) | 2 (4%)  0 (-)  1 (2%) | NR  NR  NR |
| Boeree (2017)(Boeree et al. 2017) | 1950 (35) 7/7  1200 (20) 7/7  600 (10) 7/7 | 12 weeks | Serious AE, Grade 3-4 Hep. | 63  63  123 | 4 (6%)  4 (6%)  6 (5%) | 3 (5%)  0 (-)  2 (2%) | NR  NR  NR |
| Dian ** (2018)(Dian et al. 2018) | 1350 (30) 7/7  900 (20) 7/7  450 (10) 7/7 | 30 days | Grade 3-4 | 20  20  20 | 4 (20%)  8 (40%)  3 (15%) | 4 (20%)  5 (25%)  3 (15%) | NR  NR  NR |
| Velasquez (2018) (Velasquez et al. 2018) | 975 (20) 7/7  900 (15) 7/7  450 (10) 7/7 | 8 weeks | AE 2-5 R-related | 60  60  60 | 2 (3.%)  1 (2%)  1 (2%) | 14 (23%)  14(23%)  16 (26%) | NR  NR  NR |

However, there has been renewed interest in use of high dose rifampin to improve outcomes in treatment of severe forms of active TB, specifically TB meningitis; and to reduce duration of treatment of pulmonary TB. In treatment of TB meningitis doses as high as 30mg/kg have been associated with better bacteriologic results and, in one study, reduced mortality(Dian et al. 2018; Ruslami et al. 2013; Yunivita et al. 2016). These studies enrolled small numbers of patients but reported excellent tolerability of the daily higher dose rifampin, although the evidence for long term safety over the entire treatment course is limited as the higher doses of rifampin were given for only 2-4 weeks in these studies.

Also, in a recently published retrospective study (Seijger et al. 2019) , 26 patients with severe TB illness (CNS or pulmonary) were treated with high dose rifampin (900mg/day to 1800mg/day) for 6 to 12 months. Although adverse events for these patients are not reported separately than for the 57 other patients in the same cohort (who were treated with high dose rifampin for low plasma concentrations), higher dose rifampin was well tolerated by all patients in this study.

To date, there have been no phase 3 clinical trials of high dose rifampin in treatment of active TB.

Overall, there has been no evidence of excess toxicity in studies that have used doses ranging from 20mg/kg/day, up to 35 mg/kg per day. However, in these studies, the high dose rifampin was given for only 2 weeks up to 3 months, and sample sizes were small, limiting power to detect less common events such as serious AE.

### Evidence for high dose rifamycins to shorten treatment of active TB

Rifampin: A few recent studies, summarized in Table 1, have assessed the efficacy and safety of high dose rifampin for treatment of pulmonary TB (Boeree et al. 2017; Diacon et al. 2007; Jindani et al. 2016; Ruslami et al. 2007; Svensson et al. 2018; Velasquez et al. 2018). The primary objective of these phase 2 studies was to detect differences in safety; however, they showed early bactericidal activity (Diacon et al. 2007) and more rapid sputum culture conversion (Jindani et al. 2016; Svensson et al. 2018). These outcomes are considered surrogates for end-of-treatment outcomes; hence these results suggest that shortening of treatment of active pulmonary TB may be achieved with higher doses of rifampin.

Rifapentine: Rifapentine is a drug that has similar anti-mycobacterial activity as rifampin, but has a prolonged half-life – up to 5 times longer than that of rifampin. Therefore, to achieve higher exposure to rifapentine – dosing can be once weekly, but a much higher dose is given (1200 mg once a week), or more frequent doses are given (daily 7.5mg/kg, 10mg/kg, 15mg/kg, 20mg/kg). Daily dosing result in serum drug levels that increase over the first two weeks and achieve steady state serum levels equivalent to the serum levels achieved with triple the daily standard doses of rifampin. Recent trials have tested use of rifapentine at higher doses once a week (Jindani A et al. 2014) or normal doses given daily (Conde et al. 2016; Dawson et al. 2015; Dorman et al. 2015), which results in much higher serum drug levels given the long half life of this drug. These studies showed that these higher doses achieved more rapid sputum sterilization or other favourable treatment outcomes. However, these were phase-2 studies, with limited power to detect efficacy differences.

### Evidence of use of high dose rifamycins in short latent TB treatment regimens

Mouse Models - Concurrent with studies on use of high dose rifampin for treatment of active TB, there have been efforts to assess the activity of high dose rifamycins in treatment of latent TB infection. In a mouse-model of latent TB infection, daily dosing of rifapentine has been shown to result in relapse-free cure of latent infection after as little as one month of treatment (Zhang et al. 2009). Notably, in these mouse studies rifapentine was given at the same doses ***daily*** (on a mg/kg basis) as were used when rifapentine was given weekly in previous studies. As explained before, because rifapentine has a prolonged half-life this means that daily dosing will result in serum drug levels equivalent to the serum levels achieved with triple the daily standard doses of rifampin.

Trials in humans of shorter treatment of latent TB - A trial of one month of daily INH (standard doses) and daily Rifapentine (same doses as when given weekly – which should result in much higher serum levels of RPT – as in the mouse model) was recently published (Swindells et al. 2019). In this study an “ultra short” regimen of daily self-administered INH and rifapentine for one month (1HP) was compared to 9 months daily INH in HIV infected adults with presumed latent TB. The 1HP regimen had non-inferior efficacy and was also well tolerated with very good completion rates. There were a number of important methodologic issues with this trial – most notably that latent TB was not confirmed in 80% of participants, so the non-inferior result for TB prevention may simply reflect the fact that the majority of those treated were not at risk for disease. Nevertheless, these interesting results suggest that it may be possible to successfully shorten duration of latent TB therapy without impacting efficacy by achieving higher serum levels of a rifamycin. These preliminary results also suggest that higher doses of a rifamycin can be given safely.

### Summary: Use of high dose rifampin for treatment of latent TB

Our rationale for the trial is based on the following: 1) acceptance, completion and costs of latent TB therapy are superior with shorter regimens; 2) therapy must be safe, and the current safest regimen appears to be 4R; 3) the best completion rate, which has been seen in study conditions with 4R, is still less than 80%; 4) in treatment of active TB, high dose rifampin achieves faster culture conversion; 5) in a mouse model higher rifamycin levels achieve faster clearance of latent TB infection; 6) a recent study achieved promising results with treatment of latent TB with one month of high dose Rifapentine paired with INH.

Taken together, the available evidence suggests that a shorter regimen of high dose daily rifampin may be as effective as the standard 4R. However, there is insufficient evidence to determine the optimal dose that will achieve similar efficacy as 4R, without any increase in adverse events. As a first step, in a phase 2 trial, we plan to verify that high dose rifampin monotherapy is safe, as treatment of latent TB implies treatment of otherwise healthy persons. Our concern about toxicity and motivation to assess this carefully in a phase 2 trial is based on the experience with the 2-month rifampin-pyrazinamide regimen (2RZ). In the 1990s a series of randomized trials, conducted exclusively in HIV infected populations, demonstrated non-inferior efficacy of 2RZ compared to the standard of 9-12 months INH. In these HIV infected study populations, the 2RZ regimen appeared very safe – with similar rates of hepatotoxicity as the INH regimens. Hence 2RZ was strongly recommended in guidelines in the US and Canada in 2000. But when this regimen was subsequently introduced widely in HIV uninfected adults the results were disastrous - with high rates of hepatotoxicity and even deaths due to this regimen. This regimen was abandoned, but the ‘damage’ to TB prevention was done, and many doctors, already concerned about the toxicity of INH, simply do not prescribe LTBI treatment, for fear of side-effects (Adhikari and Menzies 1995; Blum et al. 1993; Dasgupta et al. 2000; Dobler, Bosnic-Anticevich, and Armour 2018; Elzi et al. 2007; Onofre Moran-Mendoza 2004; Yuan, Richardson, and Kendall 1995)

Hence, any regimen that looks promising for TB prevention must be tested carefully for safety and tolerability in the same populations who are likely to receive this – meaning both HIV infected and uninfected, and all ages (adults and children). Rifapentine based regimens have a number of drawbacks, including concerns regarding safety due to potentially severe systemic reactions (Sterling TR et al. 2015), higher costs, and poor accessibility as this drug is not licensed in many countries. This also means providers are inexperienced with this drug, further slowing uptake. Rifampin has the advantage of being already licensed and used widely in all countries so providers are familiar with the drug, plus it has lower cost, and an excellent safety record (at standard dose). Doubling the standard dose (20mg/kg/day) to date has an excellent safety profile, albeit in small studies of patients with serious forms of disease (Boeree et al. 2017; Dian et al. 2018; Jindani et al. 2016; Yunivita et al. 2016). There is less experience with the higher doses (30mg/kg/day or more), but in studies published to date these doses have also been well-tolerated (Boeree et al. 2017; Dian et al. 2018).

Published results suggest that higher doses of rifampin or rifapentine for shorter duration may be effective for TB prevention. Hence we plan to assess the treatment of latent TB with high-dose rifampin for 2 months using a dose of 20 or 30 mg/kg (2R_20_ or 2R_30_). But higher doses of rifampin may increase risk of adverse events. Currently the 4R_10_ regimen is the **only** LTBI regimen with significantly lower rate of Grade 3-4 AE than the comparator arm of 9H in published randomized trials. This is a very important advantage, and it is crucial that any potential gain in completion from a shorter duration must not be offset by worse safety and tolerability.

Further information on rifampin use for LTBI can also be found in the attached supplementary document “2R2 Supplement document: Safety and efficacy of LTBI treatment” (Appendix 2).

# Design

Three-arm, phase 2b, partially-blind, randomized trial of patients who are recommended to take latent TB therapy. The trial will test superiority of completion and non-inferiority of safety. All members of the same household of a patient with newly diagnosed active pulmonary TB (household contacts) will be randomized together (i.e. cluster randomized). The two higher doses will be administered double-blind. This means that household members will be aware of the duration of their regimen – i.e. whether they are randomized to two months (of high dose 20 or 30 mg/kg/day) or four months (of standard dose 10 mg/kg/day) of treatment. But participants and treating teams will remain blinded to the specific dose (i.e. 20 or 30 mg/kg) for those randomized to the two-month high-dose regimens.

# Rationale:

We propose a phase 2b trial initially to assess acceptability, tolerability and safety – through the combined outcome of completion, plus a careful assessment of safety through detailed independent review of all Grade 3 or worse adverse events. This trial will have several mechanisms to ensure safety of study participants, including review within 24 hours by the coordinating centre of all adverse events associated with suspension of study drug, subsequent review by an independent panel of all potentially serious drug related adverse events, and periodic as well as ad hoc review of safety data by an independent data safety and monitoring board (DSMB). If this trial demonstrates that higher doses of rifampin are as well tolerated and safe as standard doses of rifampin, and when given for two months have better completion, this will justify a full-scale phase 3 future trial to assess efficacy of these regimens. If the highest dose (30mg/kg/day) is equally well tolerated as the 20mg/kg/day dose, then we will select the highest dose for the phase 3 trial. On the other hand, if the 30mg/kg/day dose is less well tolerated or associated with a higher rate of serious adverse events then we will use the 20mg/kg/day dose in phase 3.

We propose an open label trial for duration – because the difference in duration is the expected key determinant of treatment completion and the short duration of only two months is the major advantage for patients. Since there is a possibility of worse tolerability and safety with higher doses, the difference in duration is necessary to offset any potential disadvantages of the short higher dose regimen. We have used an open label design successfully in previous trials; to overcome potential bias in ascertainment of outcomes we will utilize blinded independent review panels for the outcomes of adverse events and active TB – as we have done in past studies (Diallo et al. 2018; Menzies D et al. 2008; Menzies et al. 2018). We will blind the two higher doses, as we expect that patients and health care providers may be more likely to report side effects with the higher doses, and the duration is the same.

We propose a cluster randomized trial for household contacts (HHC), with household as the unit of clustering – to avoid potential mix-up of medications taken by different HHC within the same household (particularly for the higher dose arms). We also wish to prevent potential problems of non-completion that might arise once some HHC have finished a 2-month regimen, while other HHC, in the same household must continue a 4 month regimen.

We propose to conduct a pharmacokinetic (PK) sub-study within this trial. This sub-study will include an intensive PK and a population PK. Although there are a few data on pharmacokinetics of higher dose rifampicin when used for therapy of active TB (Svensson et al. 2018)**,** less is known of the PK of this drug in patients with LTBI and in specific weight and age categories, particularly children above 25 kg; and adult above 60kg.

# Outcomes

## Primary outcomes

The study has two primary outcomes: regimen completion and safety

Regimen completion: we have selected regimen completion as one of the two primary outcomes because we consider this to represent a summary measure of tolerability – i.e. the combined impact of patient decision to stop due to symptoms, such as stomach upset, anorexia, or fatigue, and health care provider decision to stop based on objectively measured events such as rash, or liver or hematological toxicity. This outcome has the additional advantages of being easy to define and measure, and does not require the added judgment of investigators, or an outside panel. Treatment completion is also readily comparable to completion measured in other studies.

This outcome will be based on the number of pills dispensed and pill counts during treatment phase follow-up visits. For the 2-month high-dose rifampin regimens, treatment phase medical follow-up visits by patients will be made two weeks after the start of therapy, at 4 weeks, and at end of therapy, and for 4R_10_ after 1 and 2 months and at end of therapy (total 3 follow-up medical visits for each regimen). Completion will be defined as taking 80% of doses within 120% of allowed time (48 doses within 72 days for the two-month regimens, and 96 doses within 144 days for the 4-month regimen).

Safety: Grade 3 to 5 adverse events (and Grade 1-2 rash) which result in permanent discontinuation of the study drug, and that are considered probably or possibly related to the study drug by the majority of a three-member independent and blinded Adverse Events panel. This panel (see below for details on panel) will review all events when the study investigator / primary health care provider stopped the study drug permanently because of their concerns of a potential drug-related adverse event. The panel will review these incidents blinded to rifampin dose, and independently from each other. Grading will be standardized using criteria established by the American Thoracic Society for hepato-toxicity (Saukkonen et al. 2006) and the National Cancer Institute for all other adverse events (National Cancer Institute Terminology Criteria for Adverse Events v4.0, at: <https://evs.nci.nih.gov/ftp1/CTCAE/CTCAE_4.03/Archive/CTCAE_4.0_2009-05-29_QuickReference_8.5x11.pdf>

## Secondary outcomes

1. Grade 1 to 2 adverse events which result in permanent discontinuation of the study drug and are considered probably or possibly related to the study drug by the adverse events panel.
2. Active TB – symptoms will be ascertained monthly by direct questioning during treatment phase follow-up visits, and by telephone call every 3 months up to 26 months after randomization. This allows two years of follow-up after completion of the shorter regimen. Patients with possible active TB will be investigated following a detailed and standardized protocol. The final diagnosis will be established by an independent 3-member Active TB panel who will review all possible TB diagnoses blinded and independently. Active TB will also be determined passively by sending the names of all study participants to the local TB authorities to cross-check their list of all persons notified to have active TB against our list of study participants. (This procedure will be described in participants’ informed consents).

# Trial procedures

## Intervention arms:

Arm 1: 60 doses daily self-administered rifampin at 20 mg/kg (**2R_20_** – maximum 1200 mg/day).

Arm 2: 60 doses daily self-administered rifampin at 30 mg/Kg (**2R_30_**) - maximum 1800 mg/day).

## Comparator:

Arm 3 (standard): 120 doses daily self-administered rifampin at a dose of 10mg per kg per day (4R_10_maximum 600mg per day).

For Canadian sites, the study formulations of rifampicin 20 mg/kg and 30 mg/kg, will be procured by each site, purchased from marketed rifampin capsules (Valeant Canada or Sanofi-Aventis Canada), with dose adjustment, packaging and coding provided by another supplier (Pharmacie Linda Frayne, 5858 Chemin de la Côte-des-Neiges #400, Montréal, QC H3S 1Z1, Montreal, QC). Formulations of standard dose of rifampicin 10 mg/kg, will be procured in the usual manner as for standard of care in each site in Canada.

For Indonesia rifampin for all study arms, will be purchased from an Indonesian supplier (Jl. Indofarma No. 1 Cikarang Barat, Bekasi, Jawa Barat 17530, Indonesia). Because this supplier provides Rifampin in capsules of 300mg and 450mg, there will be no need for re-compounding.

For Vietnam, rifampin for high dose arms will pre purchased from Svizera Europe B.V. (Antennestraat 84, 1322 AS Almere, The Netherlands) and rifampin for standard arm from Mekophar Chemical Pharmaceutical Joint Stock Company (297/5 Ly Thuong Kiet Street, Ward 15, District 11, Ho Chi Minh City), Vietnam.

## Accountability procedures for investigational and comparator drugs:

For intervention arms (high dose arms) in Canadian site: the drugs for the two experimental arms, will be purchased as ROFACT 300mg (DIN 00343617), or RIFADIN 300mg (DIN [02092808](https://health-products.canada.ca/dpd-bdpp/info.do?lang=en&code=19034)). The capsules will be shipped with secure courier to the re-compounding pharmacy (Linda Frayne, Pharmacie Frayne & associés, 5858, Côte-des-Neiges suite 400, Montréal, Québec, H3S 1Z1, [(514) 342-8696](https://www.google.com/search?client=firefox-b-d&channel=crow&q=linda+frayne+pharmacy&spell=1&sa=X&ved=0ahUKEwiGpOCj9t7iAhXGV98KHfNyDIUQBQgtKAA&biw=1280&bih=608)) in Montreal. The blind, re-compounded capsules of 300mg and 450mg strength will then be shipped, also by secure courier, to the study site pharmacies in bottles. The labels for each bottle will specify if this contains the 300mg or the 450mg capsules. Each capsule (of 300mg and 450mg) will look the same, for blinding purposes and will not have anything written on it. At each site, for each participant randomized to the high dose arms, the pharmacist, or another health professional designated by the site qualified investigator, will be unblinded. This unblinded pharmacist (or health professional as above) will prepare a bottle with the number of doses needed for that participant until the next planned treatment phase follow-up visit. The bottle given to the participant will be labeled with their (participant’s) name, study ID, and a code corresponding to the arm to which participant has been randomized to. The code that identified each study arm will be known only to the pharmacist (or other designated health professional as above) at each site, designated to prepare the bottles for the participants. The code will also be known to the central study coordinator. This will ensure that the participant and his/her treating team will remain blinded.

At each visit, participants will bring their pill bottles, so that any remaining pills can be counted. Bottles will be refilled with doses needed until the next planned visit. The pharmacy at the site will keep a log of drug dispensed to participants, linking the content of the bottles prepared for each participant to the original bottle received from Linda Frayne pharmacy.

In Indonesia and Vietnam the same procedures will be used except that study drugs will be provided directly by Indofarma (for Indonesia) and from Svizera Europa B.V. (for Vietnam), as 300mg and 450mg capsules, with no need for re-compounding before use. The capsules of 300mg and 450mg will look the same for blinding purposes. At each site there will be a person in charge of keeping the codes for the blind arms, who will use the code received at randomization, to prepare the study drug supply until the next planned study visit.

For standard dose (comparator) arm: participants who are randomized to be in the control arm will be provided rifampin at the daily dose specified in the protocol, based on their body weight (10 mg/kg/day, 600mg maximum per day). In Canadian sites, the capsules of rifampin needed will be procured and provided to participants as per standard care. In Indonesia capsules of rifampin for standard arm will be procured from Indofarma and in Vietnam from Mekophar.

## Concomitant treatments

Rifampin may accelerate the metabolism and reduce the activity of many co-administered drugs. The treating team will evaluate possible interaction with concomitant medications pre-randomization (baseline) and at each follow-up visit and judge if the potential interaction is manageable or not. If an interaction is judged not manageable by the treating team, and if treating team or participant does not wish to change the concomitant medication, the treatment with rifampin will be stopped (or not started). If this is decided before randomization, the participant will not be enrolled in the study (as per exclusion criteria, see page 17), and will be offered alternative treatment and follow up for latent TB. If a new medication is started after randomization and during treatment phase, then the study drug (rifampin) will be permanently stopped. This will be reported as an adverse event (as per protocol definition) and the participant will be offered alternative treatment (such as INH) and follow-up for latent TB. Discussions about alternative treatment and follow up will be individualized by the treating team.

As a guide, a partial list of drugs with important interactions with rifampin is included in appendix 1 of this protocol. But, at any time during the trial, there may be other, new concomitant medication taken by participants, which are not included in this list. Therefore, any new medications should be checked – by pharmacists at the site, or the trial coordination center – for potential interactions.

## Population and eligibility criteria:

Study sites: Calgary, Edmonton, Montreal and Greater Vancouver (Canada); Bandung (Indonesia), and Ho Chi Minh City (Vietnam).

Participants: Potentially eligible participants are adults and children aged 10 and older, who are eligible to take LTBI treatment according to Canadian guidelines in the Canadian sites, and according to WHO guidelines in the international sites. All eligible persons must have evidence of latent TB infection defined as a positive tuberculin skin test (5mm or greater or 10mm or greater, based on National guidelines) or positive interferon gamma release assay, and active TB has been excluded. Eligible persons will include household contacts of newly diagnosed patients with active microbiologically confirmed pulmonary TB, or with other risk factors, including HIV infection, other causes of immune suppression (see <https://bmjopen.bmj.com/content/6/11/e013488> for a detailed list), and fibronodular disease on chest-x-ray (CXR). Household contacts will be defined as having slept in the same house at least one night per week or spent at least 5 hours per week visiting the house, on average, over the last 3 months prior to the diagnosis of the index TB patient.

### Inclusion criteria:

1. Adults, and children aged 10 and older (see rationale for exclusion of younger children below) who weigh at least 25kg.
2. Evidence of latent TB infection: positive tuberculin skin test (5mm or greater or 10mm or greater, based on National guidelines) or positive interferon gamma release assay.
3. Eligible to take latent TB treatment according to Canadian guidelines in the Canadian sites, and according to WHO guidelines in the international sites. This include household contacts, other contacts, HIV infected, other causes of immune suppression, fibronodular disease on CXR, or other indication.

### Exclusion criteria:

1. Children aged 0-9 and children aged 10 or older who weigh less than 25kg
2. Pregnancy
3. Baseline AST or ALT that is at least 3 times higher than upper limit of normal
4. Baseline Grade 3-4 abnormalities of hematological tests (WBC, platelets or hemoglobin)
5. Prior treatment for latent or active TB.
6. Rifampin contra-indicated – due to potential drug interactions that are considered too important, or difficult to manage, by health care provider; or due to history of allergy/ hypersensitivity to rifampin, rifabutin or rifapentine.
7. Household contacts of index TB patients with confirmed, or suspected rifampin resistant TB will be excluded.

In centers where culture and drug sensitivity testing is routinely performed for all patients with active TB, then the index TB patients must have rifampin sensitive TB.

In settings where phenotypic drug susceptibility testing (DST) are not available, but GeneXpert is available, then the index patients must have a GeneXpert result predicting rifampin susceptibility.

If neither DST nor GeneXpert are available, then the index TB patients must have no prior TB therapy (i.e. **new** case) to reduce the probability of drug resistance. (In the two international sites the prevalence of rifampin resistance (MDR or RR) among previously untreated patients with newly diagnosed active pulmonary TB (new cases) is respectively 4.1% in Vietnam and 2.4% in Indonesia (according to WHO Country profile, 2017).

Note: In settings with phenotypic DST, household contacts may be enrolled and randomized before the DST results are available, as long as the index TB patient is not expected to have drug resistant TB. If a HHC is enrolled and later the DST reveals rifampin resistance in their index TB patient, they can be excluded post-randomization.

Valid exclusions post-randomization:

In settings where the results of required laboratory tests are not available on the same day, participants, without a history of liver or hematological disease, may be randomized before all test results are known. In this situation, randomized participants will be informed that they should NOT start therapy until they are informed by research staff that their initial laboratory test results are normal. If any of the lab tests are abnormal, and exceed levels specified in the study’s exclusion criteria (for example, liver transaminases are more than 3 times upper limit of normal, or pregnancy test is positive), participants will be told not to start the study medication and will be excluded per protocol post-randomization. In this situation the coordinating center will be informed, and participants will be referred back to the treating team for re-assessment. If the treating team decides to give LTBI treatment, medications and all follow-up will be given through routine care.

In patients with history of liver or hematological disease the lab test results must be available before randomization.

### Rationale for exclusion of children <10 years old and inclusion of children aged 10-17:

There is little published data regarding the pharmacokinetics (PK) of high dose rifampin in children. Therefore, we think it is important to acquire this information in a pediatric population that would otherwise receive doses of treatment based solely on studies in adults.

Previous studies have shown that the metabolism of rifampin is different in young children (under 5 years of age) than in adults. When using standard doses of rifampin, plasma maximum concentrations and AUC were lower among children under five than in older children and adults (Verhagen et al. 2012). The recommended dose for children less than 25kg is therefore 15m/kg/day, with a range 10 to 20mg/kg/day (World Health Organization 2014) , compared to 10mg/Kg for adults. Hence, we think that the population of children under five, for which a higher daily dose (up to double) is already recommended for standard therapy, will not be suitable for this first trial on safety of higher doses of rifampin in LTBI.

In our recently concluded 4R vs 9H studies, of the 422 children enrolled in the 4R arm, 215 were 10 to 17 years old. Among these children, there were no Grade 3-4 adverse events at all with daily rifampin at 10mg/Kg/day (Diallo et al. 2018). Furthermore, adults in the same trial, had significantly fewer Grade 3-5AE with daily rifampin at standard doses compared to those taking daily INH (Menzies et al. 2018). Hence, we believe that the risk of serious adverse events for rifampin at standard dose or at high dose should be lower in children aged 10-17 than in adults. As well, there is published evidence that older children have similar metabolism as adults; therefore WHO recommends adult dosing (i.e. 10mg/kg with maximum 600 mg) for children who weigh 25kg or more (World Health Organization 2014).

There is some published evidence of safety of high dose rifampin among adolescents, although the number of adolescents who were included in these previous studies is very small (Dian et al. 2018; Yunivita et al. 2016). Because older children and adolescents are expected to have metabolism of rifampin similar to adults’, and because no specific side effect was found in the adolescents included in previous studies of TB meningitis (Dian et al. 2018; Yunivita et al. 2016), we believe it is reasonable to include in this study children with age of 10 or older who weigh at least 25kg. This will allow a more thorough assessment of the safety of high dose rifampin in this population, and will provide pharmacokinetic data to establish the ideal dosing.

If the higher doses are shown to be safe and well tolerated in adults and children aged 10 years and older, then we will plan an initial intensive PK study to determine the doses needed in children aged 0-9, followed by a phase 2 study to assess safety in this age group of children, with careful adverse event monitoring.

# Sample size considerations

**For primary outcome of completion:**

We want to detect whether the 2R_20_, and 2R_30_ regimens have superior completion compared to 4R_10_. If the two months regimens do not have significantly superior completion rates, then the rationale to further assess these regimens would be weaker, since the 4R_10_ regimen has the advantages of well documented safety and efficacy.

**Table 2: Sample size required to detect superior completion of 2R_20_ or 2R_30_ compared to 4R_10_**

| Completion rate | | Number required per group to detect significant difference*. | | | |
| --- | --- | --- | --- | --- | --- |
| **4R_10_** | **2R_20_ or 2R_30_** | N per arm – non-HHC | N per arm –HHC | N per arm – mixed | Total N  (3 arms) |
| 70% | 75% | 1248 | 2484 | 2075 | 6226 |
|  | 80% | 290 | 578 | 483 | 1450 |
|  | 85% | 118 | 235 | 196 | 589 |
| **75%** | 80% | 1091 | 2172 | 1815 | 5446 |
|  | **85%** | **247** | ***493*** | ***412*** | ***1237*** |
|  | 90% | 97 | 193 | 151 | 453 |
| 80% | 85% | 903 | 1797 | 1505 | 4515 |
|  | **90%** | 196 | 391 | 306 | 917 |
|  | 92.5% | 115 | 230 | 180 | 540 |

**Power = 80% and alpha = 0.05. The calculation of total number required is based on 3 assumptions: (i) 65% of all enrolled subjects will be Household Contacts (HHC), and 35% will be other risk groups, as was seen in our recently completed 4R trial; (ii) The intra-class correlation coefficient (ICC) or clustering effect of households on completion will be 0.33 – as was demonstrated among study subjects who had at least one other family member in the 4R vs 9H study; (iii) We will use 4 as estimated average number of household contacts, based on two systematic reviews (Morrison J, Pai M, and Hopewll PC 2008; Fox GJ et al. 2013).*

In the 4R vs 9H trials, 78% of adult participants in the phase 2 trial (Menzies D et al. 2008), and the phase 3 trial (Menzies et al. 2018) completed the 4-month regimen. Assuming a similar rate of completion in this planned trial of 75%, then we would need to enroll **412** participants into each arm to detect a 10% better completion rate. Allowing for a 10% withdrawal, or otherwise not analyzable participants, this would inflate the number per arm to 453, so we plan to enroll a total of **1359** participants. As seen above, this number would provide limited power to detect smaller differences in completion. For example, 1815 participants per arm would be needed to detect a 5% better completion rate.

The approximate anticipated number of participants enrolled in each study site is as follows: 100 in Calgary, 100 in Edmonton, 100 in Montreal, 150 in Greater Vancouver (Canada); 500 in Bandung (Indonesia), and 450 in Ho Chi Minh City (Vietnam). Enrollment will be closed when the target 1359 is reached.

**For the primary outcome of safety:** Grade 3-4 Adverse events considered possibly/probably related to study drug:

We want to detect whether the 2R_20_, and 2R_30_ regimens are *not worse* than 4R_10_ in terms of serious adverse events (SAE) that are plausibly related to the study drugs, as defined above. Hence this requires a non-inferiority analysis, and the sample size must be adequate to detect whether the high dose rifampin arms are non-inferior to standard 4R in terms of drug-related SAE.

As shown in Table 3 below, we expect the SAE rate with 4R to be 1% (as seen in the 4R vs 9H phase 3 trial) or 2% (as seen in the 4R vs 9H phase 2 trial), but have also included estimates if the SAE rate is as high as 3%. We define the maximum allowable (maximum tolerated) difference to be **4% more**. (*This is fairly stringent, but we believe a stringent non-inferiority margin is needed, since for an LTBI regimen to be acceptable, it is very important that the regimen is safe. Indeed safety is ESSENTIAL for an LTBI treatment regimen – as these are given to otherwise healthy persons.*) We must account for the clustering effect of households, since, as above, we expect that up to 65% of our participants will be HHC. However, in the just completed 4R vs 9H trial, the cluster effect (or ICC) in terms of serious AE was only 0.05, so the impact on sample size is much less than for completion.

**Table 3; Sample size required to conclude non-inferiority of** **2R_20,_ or 2R_30_ in terms of SAE compared to 4R (***Maximum tolerated difference = 4%)*

| Grade 3-5 Adverse event rate | | Number required per group to conclude non-inferiority. *(Assumes 4 contacts per HH, and ICC = 0.05)* | |
| --- | --- | --- | --- |
| **4R_10_** | **2R_20_ or 2R_30_** | N per arm | Total N – for 3 arms |
|  | | | |
| **1%** | **2%** | **234** | **702** |
|  | 3% | 698 | 2094 |
|  | 4% | 3468 | 10,404 |
|  | | | |
| **2%** | **3%** | **386** | **1158** |
|  | 4% | 1035 | 3105 |
|  | 5% | 4805 | 14,415 |
|  | | | |
| 3% | 4% | 534 | 1602 |
|  | 5% | 1366 | 4098 |
|  | 6% | 6114 | 18432 |

** In this table, for simplicity, all subjects are assumed to be HHC – since the ICC is low and has very modest effect on sample size required.*

Table 3 shows that if SAE rate is only 1% in 4R_10_, and 2% in **2R_20_** or **2R_30_**, then we would need 234 patients per arm, or 702 patients in total to conclude that the high dose regimens were non-inferior to the standard dose for serious adverse events. But, if the real adverse event in the higher dose arms is 3% (ie 2% higher) then we would require 698 per arm, or 2,094 total participants. Hence, we run the risk of not having enough power to conclude non-inferiority if the actual serious adverse event rate is as little as 2% higher. This means we will ‘tolerate’ a very small excess in adverse events. If the actual SAE with **2R_20_** is 4% then the number needed to conclude non-inferiority increases even more markedly. On the other hand, if the SAE rate with 4R is actually 2% (as in phase 2) then the sample size required will be larger. But, with the 1359 anticipated enrolment needed to address our primary objective of completion, we should have adequate power to conclude non-inferiority if the high dose regimens have 3% rate (1% greater occurrence) of serious adverse events.

# Statistical analysis

Primary analyses

The are two planned primary analyses:

1) Superiority of treatment completion: treatment completion is defined as taking at least 80% of the recommended doses within 120% of allowed time. Since this is a dichotomous outcome, this primary analysis will be a logistic regression, using an identity link, and estimated via generalized estimating equations to account for clustering by household. An exchangeable correlation structure and empirical standard errors will be used. We will compare the proportion having treatment completion in each high dose arm against the standard arm – 10 mg/kg/day rifampin given for 4 months.

Given that some exclusion post randomization could occur (for example, contacts are enrolled, but the DST results of their index case were not available at the randomization, then later the DST shows resistance to rifampin), a modified intention to treat analysis will be used – with these valid post-randomization exclusions not included in the primary analysis. A secondary analysis will be done among patients who completed therapy per protocol.

2) Non-inferiority of safety: analysis of grade 3-5 adverse events will be as for relatively rare dichotomous outcomes. Poisson regression will be used to compare the occurrence of the grade 3-5 adverse events between each of the two experimental arms and the conventional arm. To account for clustering by household, we will use Generalized Estimating Equation (GEE), with an exchangeable correlation structure and empirical standard errors. To assess non-inferiority, we will use the confidence interval approach, and compare the upper limit of the difference versus a margin of 4%.

Secondary analyses:

Non-inferiority of completion. A planned secondary analysis will assess non-inferiority of completion. The maximum allowable (maximum tolerated) difference will be **5%, with one-sided significance level.**  With the number of participants we expect to enroll for the primary analysis (1359), we will have 80% power to show non-inferiority if any of the experimental arms has 5% higher completion than the control arm, assuming the completion in control arm will be between 70 and 80 % (as it was in the previous RCT).

- We will compare the occurrence of grade 1-2 adverse events reported by study investigators and judged by the independent blinded adjudication panel to be possibly or probably related to study drug in the same way as grade 3-5 adverse events.

- Active TB – the incidence of active TB (microbiologically confirmed and all forms) in the 26 months post-randomization per 100 person-years of follow-up will be compared between all three arms.

All analyses will include adjustment for clustering by household. In stratified analysis, results will be presented by indication for LTBI treatment. Sensitivity analyses will be conducted where by analysis are stratified by study centre and by country.

Interim analyses

To optimize protection of safety of study participants, we plan at least two interim analyses of study regimen completion and of Grade 3-5 adverse events. The first analysis will be conducted after the first 150 participants randomized (i.e. approximately 50 in each arm) have completed therapy. This means we will assess outcomes that have occurred in each of these individuals up to 146 days post-randomization. The second interim analysis will be conducted 146 days after randomization of the 450^th^ Participant – i.e. after approximately 150 participants should have completed therapy in all 3 arms. However, as described below, the 2^nd^ analysis may be performed sooner if there are concerns about excess toxicity with either high-dose arm.

Our goal is to identify as early as possible if the high dose rifampin regimens are associated with a clinically important increased rate Grade 3-5 AE, or intolerance – defined as failure to complete therapy. The study bio-statistician will conduct both interim analyses in a blinded fashion. Given that there are two planned interim analyses, the p value -adapted for multiplicity of testing- that will be used to consider significant the difference between proportions is of p<0.022 (Pocock’s method).The Data Safety and Monitoring Board (DSMB – see below for more details) will also consider results while remaining blinded to the identity of the regimens. For each interim analysis, if any of the regimens meets criteria for a clinically important difference in adverse events or completion, then we will unblind in step-wise fashion. If unblinding is deemed necessary, the person in charge of code breaking will be the principal investigator (Dr. Menzies) and the study coordinator (Dr. Federica Fregonese). Same procedures will be followed if DSMB asks for unblinding in specific cases for serious and unexpected adverse events.

Most clinical trial experts do not suggest formal statistical stopping rules for safety. Instead, the DSMB will receive frequent safety reports and will “make judgments about the strength of the evidence and the absolute magnitude and seriousness of any safety signals” (Pocock, Clayton, and Stone 2015). As approximate guidance, we will suggest to the DSMB that for the 1^st^ interim analysis non-completion must be at least 10% worse and/or grade 3-5 adverse events more than 10% more frequent than the standard arm. For the 2^nd^ interim analysis occurrence of grade 3-5 adverse events would have to be 7.5% worse, and treatment completion at least 7.5% worse to consider dropping that arm. Grade 3-5 adverse events are very uncommon events with 4R (1-2% in our prior trials), meaning that we expect zero or only one AE in the standard arm with 50 participants. Hence occurrence of Grade 3-5 AE in only 5 or 6 of the first 50 participants randomized to either experimental arm would result in an AE rate difference of 10% for the 1^st^ interim analysis. This could happen due to chance alone. At the 2^nd^ interim analysis – we expect 2-3 persons with Grade 3-5 AE among the 150 randomized to 4R_10;_ to detect a difference of 5%, would mean at least 9-10 participants in the highest dose arm would have to have Grade 3-5 AE, or 18-20 participants with these AE in both high dose arms, before stopping the trial; this number with AE would be very unlikely due to chance alone. But, as noted above – these are guidelines only that will be suggested to the DSMB. The decision to stop enrolment to one or both experimental arms will be based not only on these differences, but also how serious the adverse events are. The threshold to stop will be lower if the events are more serious. As well, the DSMB can request more frequent analyses of adverse events – if there is any evidence on greater rate of AE with the higher dose arms.

We also do not wish to unblind the DSMB unnecessarily as this can bias the DSMB decisions. First, we will reveal if the arm with higher rate of AE/lower rate of completion is the standard 4 month arm. If so, we will not reveal the identity of the other two arms, and enrolment to all three arms will continue. If the arm with substantially higher rate of events of Grade 3-5 AE or non-completion, is one of the two experimental arms then we will reveal the identity of all three arms to the DSMB. If the 30 mg/kg arm has the excess events, but not the 20 mg/kg arm – then we will stop enrolment to the highest dose arm. On the other hand, if the 20 mg/kg arm has the excess events, but not the 30 mg/kg arm, this will be considered more likely due to chance (since it is biologically less plausible). In this situation we will combine results from the two high dose arms, to determine if the combined outcomes are considered a clinically important difference in AE or completion compared to the standard arm. If the DSMB believes these criteria are met – then enrolment to the trial will stop, and we will report results.

The criteria to conclude that an experimental arm has higher rates of Grade 3-5 AE or non-completion that are clinically important (the “stopping rules”) will be different for the two interim analyses, as we want to guard against a ‘false positive’ results. We wish to avoid concluding incorrectly that a high dose regimen has excess toxicity, and therefore stopping enrolment. One safeguard against falsely concluding the rate of non-completion is worse with the high dose regimens is that completion should be better, since the regimen is shorter. Hence if it is actually worse, then the most important presumed benefit of the regimen is actually not achieved. (*Interestingly, the infamous 2RZ regimen described earlier* ***did not have better completion rates*** *than the much longer comparator regimens of 6H or 12H (Gao et al. 2006) – a potentially important warning of the 2RZ regimen’s poor tolerability. This finding was ignored in 2000, but recognized a year later in the ‘post-mortem’ analyses of the 2RZ failure*).

# Randomization

Randomization will be by individual, except if there are more than one participant from the same household. If a HHC is enrolled and belongs to the same household as someone who has already been enrolled and randomized, that HHC (and all subsequent HHC from the same household) will be allocated to the same regimen *as long as they are enrolled within 14 days*. If they are enrolled later, they will be excluded. This is to avoid preferential enrollment of patients in the same household who may deem one regimen more attractive than the other, as was seen in our earlier 4R vs 9H trial.

Randomization will be computer generated and web-based. Investigators will enter all information from the initial case report form (baseline CRF). The randomization program will verify eligibility, and whether another member of the same household has already been randomized. Randomization will be in blocks of variable size, and stratified by country, and within Canada by city. The web based programme will be developed by collaborators (Eric Rousseau and team) at the Laboratoire de télématique biomédicale (LTB) du Réseau en santé respiratoire du Québec (RSR), Sherbrooke, QC, who were responsible for all web-based data entry, randomization and adjudication panel work in our prior multi-centres trials (for demo see 2R^2^ link: [http://2r2-demo.crc.chus.qc.ca](http://2r2-demo.crc.chus.qc.ca/)  ID: 2r2demo Password: demo)

# Data gathering:

## Quality management:

The study will be conducted in compliance with this protocol and the ICH-GCP in all sites. In each site, requirements of the Country health authority (Health Canada for Canadian sites) will be followed.

To ensure high quality data, all staff at all participating sites will receive initial training in the study protocol, procedures, and data requirements. This will include training in good clinical practice (GCP). Note that all sites participating in this trial have previously participated in randomized trials, notably our 4R vs 9INH trial, and have previously received GCP training. Hence for most this will be refresher training.

Study sites will also receive periodic monitoring visits during the conduct of the trial, to ensure that the study is conducted in accordance with protocol and to check for quality of data collected.

The principal investigator at coordinating site and the principal investigators at study sites, will be in charge of maintenance of the master binder with essential documents of the study, following GCP recommendations for the documents with participants’ confidential information.

During monitoring visits from the coordinating center, and if any visits occur for auditing, REB review and regulatory inspections, study site investigators will provide direct access to all essential documents including source data and documents.

Data management of collected data will be done during the study and before final analysis by the coordinating center. Queries will be sent to study sites to correct erroneous data or complete missing information. All data collected will be used in primary and secondary analysis.

## Pre-treatment:

Potential participants who may meet the eligibility criteria will be asked by the treating team, if they would like to meet research staff. Those who are not interested in participating will receive standard care from the treating team. For those who accept to meet with study team, their eligibility criteria will be checked and those who are eligible will be asked to provide informed consent. For those who consent a baseline evaluation will take place. In this baseline evaluation, all usual clinical and demographic information will be collected including indication for LTBI treatment (i.e. risk factors as being a close household contact, having immunosuppression, etc).

A close household contact (HHC) will be defined as persons who, over the past 3 months, slept in the same house at least one night per week, or spent at least 5 hours per week in the same house as a patient with newly microbiologically diagnosed active pulmonary TB (index TB patient). In sites in which DST or GeneXpert is done routinely for pulmonary TB patients, DST or GeneXpert results for rifampin for the index patient of each HHC will also be recorded.

Prior to enrolment all potential participants must have no symptoms suggestive of active TB, a chest x-ray (within a maximum of 6 months from the date of enrolment) that is considered to have no evidence of active TB, and a positive TST or IGRA as defined above. If the potential participants have abnormalities on chest X-ray, or TB symptoms, they will undergo investigation including sputum AFB smears/ TB cultures and/or GeneXpert. If these investigations to exclude active TB are negative, then they will be eligible for enrolment.

A pregnancy test will be performed prior to enrolment for all women of child bearing potential, unless the treating team judges it is not appropriate. For adolescents, results of pregnancy test will be disclosed to participants who are 14 years old or older and to participants and caregivers if less than 14 years old. Participants who are found to be pregnant will not be enrolled in the study, but an alternative individualized plan for LTBI management will be discussed with treating team and patients.

## During treatment

Patients will be seen by their primary health care providers after 2, 4 and 8 weeks of treatment for those randomized to **2R_20_**  or **2R_30_** and after 4, 8, and 16 weeks of treatment for those randomized to **4R_10_**. In this way the number, and approximate spacing of visits with their health care providers will be the same for all arms, to minimize any effect of visit frequency, and intensity of follow-up, on treatment completion. During these treatment phase visits, patients will be examined and questioned regarding symptoms of active TB as well as symptoms of adverse events. Participants will be asked to bring their medications with them at all visits. Pill counts will be done, and pills for the next treatment interval dispensed. The number of doses taken will be calculated at each visit. If sub-optimal adherence is noted (less than 80% of doses dispensed) the participant will be counselled by the usual treating team, following normal clinic procedures.

If a participant does not come on time for a scheduled treatment phase follow-up visit (is a no-show), they will be reminded – by the usual treating team and following usual procedures for non-study patients at the study site. Patients who decide to stop therapy – because of intolerance, or other reasons, will be encouraged to restart and complete therapy – by the usual treating team, and following normal procedures for non-study patients at the study site. If they maintain the decision to stop, then the study personnel will explain the need for post-treatment follow-up for two years. In our prior Phase 3 trial, while almost 30% of all patients stopped study treatment early, less than 5% were lost to follow-up. This was achieved through balancing the messages given to patients who were considering stopping therapy, or had sub-optimal adherence – emphasizing the desirability of completing study therapy, but also that they have the right to decide to stop taking therapy, or switch to alternative therapy, without any negative consequences to their care.

Routine blood tests for hemogram (hemoglobin, platelet counts, and leukocytes), and liver transaminases plus bilirubin will be drawn for all participants pre-treatment and after one month. For participants in the two high dose treatment arms, the same tests will also be done at the first follow-up visit after 2 weeks of treatment. For all participants additional tests can also be ordered at the discretion of the treating doctor. Creatinine and blood urea nitrogen will be tested after one month. The same protocol for lab testing will be followed in adults and children. Two blood draws (at 2 and 4 hours after drug intake) will be done after one month in all participants for population PK sub-study (see below). Information on rifampin drug levels will be collected for these patients in specific CRF.

If active TB is suspected then this will be investigated, managed and reported following a strict protocol. If active TB is diagnosed at another center then the study staff will travel to that center to obtain all relevant information including results for microbiological and radiologic and historic tests.

## Post-treatment

Patients will be contacted by telephone (or home visits if necessary) every 3 months after end of treatment until 26 months post-randomization. During these calls patients will be questioned regarding current symptoms of active TB, and any intercurrent diagnoses of active TB, or hospitalization for any reason. At the end of the follow-up period, home visits will be done for participants who could not be contacted by telephone or other means. If at any time active TB is suspected – then this will be investigated, managed and reported as specified above.

Every effort will be made to minimize loss to follow-up post-treatment.

## Pharmaco-kinetics (PK) studies

Two PK sub-studies will be conducted – an intensive study to determine the PK profile over many hours in a small group of participants at a single site, and a population PK – with limited sampling in a larger group of participants at all sites to assess determinants of variability in a diverse study population (age, sex, weight, ethnicity). Both PK sub-studies will be conducted after 4 weeks of therapy in all study arms, because we expect rifampin to reach steady state by this time. The population PK sampling will be performed at the same as the 4-weeks follow-up visit. The exact timing of the intensive PK sampling will be decided with the participants.

**Intensive PK sub-study**: This intensive sampling PK will be done in one of the study sites, in Bandung, Indonesia. Nine participants per arm in two age groups: 10-17, and 18 or older, will be recruited for the intensive PK sub-study (total 54 participants). On the PK sampling day, the participants will be asked to refrain from food at least 8h (4 h for children aged 10-17) before witnessed drug intake of a single dose of the study regimen at the clinic. They will remain fasting until 2 h after administration of the study drug. Serial venous blood samples will be collected just before, and at 1, 2, 4, 8, and 12 h after drug intake. Serial sampling will use an indwelling venous catheter (inserted with a standardised method) and a minimum of 1 cc of blood will be withdrawn at each sampling time. Blood samples will be immediately centrifuged and plasma will be stored at -80^0^C before bio-analysis using UPLC with a validated methods (Ruslami et al. 2007) at the pharmacokinetic lab of Dr. Ruslami (the site PI in Indonesia) in Bandung, Indonesia (Pharmacokinetic Laboratory, Faculty of Medicine Universitas Padjadjaran, Bandung, Indonesia.  Jl. Prof. Eykman No. 38, Bandung 40161,Indonesia). To obtain PK parameters of rifampicin, PK analysis will be done using standard noncompartmental methods in Phoenix WiNonLin version 6.3 (Ruslami et al. 2007).

**Population PK sub-study**: Samples for population PK sub-study will be collected in all participants. All samples collected for PK population sub-study can be utilized only for PK studies within this trial. Two blood samples (of 5 ml each) per subject will be taken 2h and 4 h after drug intake (van Beek et al. 2019). On the day of PK sampling, subjects will take their study drug at home with empty stomach. This will be self-administered and not supervised although they will be asked to record the exact time they took that day’s dose. At the clinic blood samples will be withdrawn using single sterile needle. Blood samples will be immediately centrifuged, plasma will be divided in 0.5ml aliquots and stored at -80^0^C. Frozen plasma samples from sites in Canada and Vietnam will be shipped (on dry ice) to the pharmacokinetic lab of Dr. Ruslami in Bandung, Indonesia, for the PK bioanalysis. All sampling process and bioanalysis afterward will be the same than for Intensive PK. To estimate the individual rifampicin exposure (AUC), PK analysis will be done with population PK approach (van Beek et al. 2019).

Information for the population PK will be contained in the consent for all participants at all study sites. Participants will have the option to agree to participate only in the main study – ie they can opt out of the population PK sub-study if they wish. Participants in the intensive PK sub-study at the Indonesia site will be asked to sign a separate consent for this sub-study.

## Enrolment and follow-up during COVID-19 related measures

During the COVID-19 pandemic, the following alternative procedures can be used for enrolment and follow up, although these alternate procedures must be approved by the local ethic committee, before being used. Site investigators will decide which procedure to use considering: the COVID-19 precautions in place at their site for health services delivery and the specific risks for each participant.

*Alternative enrolment procedures:* If the TB clinic operates so that LTBI treatment is started during a virtual (not-in-person) visit, enrolment can also be done virtually. The medical team that is recommending LTBI treatment will inform the potential participant of the study, and if the potential participant agrees, then the research team will contact them by phone. They will explain the study and read the consent form fully to the potential participant; if that person agrees, the same research staff person will sign the last page, attesting verbal agreement to participate in the trial.

*Alternative follow-up during treatment*: if TB clinic operates so that patients on LTBI treatment are followed virtually, then research follow-up visits can also be done virtually. Virtual visits are conducted by the treating team, and the research personnel will obtain the protocol required information from the treating team and may call participants if other, study specific, information are needed.

If any symptoms or other information is reported by the participant during a virtual follow-up visit that indicate the need for an in-person visit for a medical examination or lab tests this in-person visit will be scheduled as soon as possible.

Note: blood tests will be done following the planned schedule (i.e. pre-treatment, at 2 weeks and at 4 weeks after treatment starts for participants in high dose arms; pre-treatment and at 4 weeks for participants in standard dose arm).

*Data gathering during virtual visits*: data collected during virtual visits will be for most part the same that previously planned, as most information can be asked to the participants by phone or retrieved by the electronic participant’s chart (i.e. the laboratory results; radiology and microbiology results; participant’s weight).

The following two information will be collected differently during virtual visits:

- Medical exam at pre-treatment visit: if participant does neither report any respiratory nor any other symptoms, the research team will report the medical exam as “not done”.

- Count of remaining pills at each follow-up visit: during virtual visits the research team will ask the patient to report on remaining pills they have at home, so that the count will be “estimated” by participants instead of done in person by research team.

*Study drugs dispensing*: if follow-up is done virtually, dispensing of study drugs should happen at the same time as samples are drawn for blood tests. This means that the study medication would be given to the participants once they come at the clinic for the blood test (so that there are not extra in person visit for these participants), with attention to minimize the waiting time for the medications.

If study drugs must be dispensed at a different when the blood samples are drawn, or if the blood tests are done elsewhere than in the clinic, the study drugs can be delivered to the participants by courier.

During all in-person visits research personnel will comply with the procedures to minimize COVID-19 transmission risk that are in place at the clinic.

*Pharmacokinetic population study*: the blood sampling for the population PK component of the study will be done only if it is considered this will not cause any additional risk for COVID-19 transmission.

# Methods to ensure the safety of study participants:

Risk and benefits of use of rifampin and of high dose rifampin are detailed in the consent form and reviewed in section “Background” of the protocol (pages 6-11).

Rifampin is a safe drug that has been used by millions of people to treat TB disease for about 45 years. Minor side effects of rifampin include headache, tiredness, weakness, gastro-intolerance, diarrhea, itchiness, rash or loss of appetite. These usually resolve spontaneously, and it is not necessary to stop the treatment. More serious reactions, such as hepatitis, are more rare. Usually hepatitis resolves once treatment is stopped.

Rifampin can also cause tears, sweat, spit, bowel movements and urine to turn orange during treatment. Contact lenses might stay stained with this orange color.

Rifampin can also affect other medications, see more details in “Concomitant treatment” at page 16.

Side effects are expected to be similar in nature to standard dose, and frequency has been similar when used in previous studies, as reviewed in “Background” (pages 6-11).

This trial will have several mechanisms to ensure the safety of study participants. During treatment phase participants will be followed closely with regular visits at which questions are asked about occurrence of new symptoms as well as laboratory monitoring as described above. All new symptoms will be recorded and managed with appropriate follow-up by the treating team. Participants will also be encouraged to call or visit the study centre if they experience any new symptoms. If at any time during treatment (up to 30 days after end of treatment) an adverse event is suspected by study staff, or treating team, this will be investigated, managed and reported following strict protocols that vary according to the type of adverse event. (Note: for possible adverse events occurring after end of treatment, investigations needed and reporting is decided on a case by case basis by site investigator and coordinating center). Adverse events of grade 3 or 4; and rash of any grade, that are considered possibly or probably drug-related will require study medication **permanent discontinuation**. Providers may, on an individualized basis, decide to continue or restart the study drug if the AE was a mild rash (Grade 1-2) or an alternate explanation was found. For example, another drug or food was ingested that could have caused the rash. In these circumstances close follow-up (i.e. daily or every other day) is required and if the rash worsens, management should be re-evaluated.

For other suspected Grade 1-2 drug-related adverse events, the treating team will decide if study medication should be discontinued on a case by case base. If treatment can be continued, the symptoms will be noted in follow-up forms, and the patient followed as judged necessary by the treating team. If the study drug is stopped by the treating team for more than 48 hours because of a suspected adverse event, including a new potential drug interaction, the site investigator must enter an initial AE report on the web-site, within 24 hours of deciding to stop the study drug, or becoming aware of the event. Note: this initial report must be completed for all events of Grade 3 or higher even if they do not require drug discontinuation. This initial report triggers an automatic notification to the coordinating centre, who will review the report within 24 hours. The PI will be notified immediately of all adverse events that are Grade 3, 4 or death. Staff at the coordination centre will contact the site PI to verify the situation, remind them of the procedures for diagnosis and management of a suspected outcome. These staff will continue to monitor the AE – as it is investigated and managed. The DSMB (see below) will be notified immediately of all Grade 3-4 AE that the PI believes were not anticipated, and were possibly related to the study regimens, and any deaths that the PI believes could possibly be related to the study regimen. Unexpected serious adverse events related to study drug, will also be reported to local IRB and to Health Canada.

When the investigation has been finalized (and/or the event has resolved), the site PI will complete the AE final report. This will be reviewed by coordinating center staff (unblinded), who will verify if the report is complete, and check that no information about the regimen used is included in the report. Once this is completed the PI (DM) will review the blinded report. This is to check again for any event that was serious and unanticipated (which are to be reviewed immediately by the DSMB). This will also serve as a final check to ensure that the report is complete and coherent enough that the adjudication panel can render judgments.

Then the full blinded report will be made available to the 3-member panel for adverse events. This panel will function as in our prior 4R vs 9H trial. All three panel members will make independent judgements about the AE – severity, type, and likely attribution to study regimen (unlikely related, possibly related, or probably related). If judgments are discordant we will take the majority opinion (two out of three). If all three opinions are discordant, then the panel will be asked to re-review. If the panel raises concerns about a particular AE report, these concerns will be communicated to the PI, and to the chair of the DSMB at the same time.

These same procedures will apply if virtual follow-up is done. In case participants need to be seen for investigations and follow-up related to a possible adverse event an in-person visit will be scheduled as soon as possible.

Participants who permanently stop treatment with high dose rifampin will discuss alternative options with their treating medical team. During initial site training, medical teams at participating sites will be informed of the different options for participants. Depending on the reasons why high dose rifampin was stopped the alternatives could be: taking rifampin, but at the standard dose, taking an alternative LTBI treatment approved by the national TB programme of the country (such as INH) or having close follow up with periodic symptoms screen and chest-x ray for early detection of active TB. The choice of the alternative would be individualized and decided by the primary health care provider and the participant.

All participants who permanently stop the study drug (either in high dose or in standard dose arm) will be followed after the stop of study treatment as per “Follow-up post treatment” procedures of all other participants who completed the study treatment, until 26 months from randomization.

At the end of the post-treatment follow-up, all participants will be provided with instructions about symptoms of active TB, and how to seek medical care if symptoms of active TB arise. They will also be given a card with information on the trial and contact information for the treating and research teams. The card will specify that the efficacy of the experimental treatment is not yet proven and therefore the risk of developing active TB could be higher than in people treated with the current standard regimen.

Data Safety and Monitoring Board (DSMB)

The DSMB will be responsible to review two planned interim safety analyses, also will review on an ad-hoc and immediate basis any Grade 3-4 adverse events that are unexpected, as well as any deaths that occur during treatment phase (regardless of the judgement about possible role of study drugs in the death). Ad-hoc reviews of unexpected serious adverse events and deaths will be done whenever needed.

Note that a few serious adverse events (but not deaths) can be *expected* as these occurred in our prior trials with 4R_10._ These will not be brought to the attention of the DSMB when they occur, but will be included in all safety analyses, and they are also mentioned in the consent, and will be explained to the potential participants’ during the consent process.

The two planned interim safety analyses, and procedures for unblinding are explained in more detail in Statistical analysis section (page 21). The DSMB will review the results of these two planned analyses and make recommendations regarding continuing or stopping enrolment to study arms, or the overall study. All DSMB recommendations, and the results of the planned interim analyses will be sent to the research ethics committees at all participating sites, and the trial steering committee.

# Adjudication panels for secondary outcome ascertainment:

We will have two separate 3-member outcome adjudication panels – one for active TB, and the other for adverse events, as in our prior 4R vs 9H trial. When an outcome requiring adjudication (AE, or active TB) occurs or is suspected the site must enter a preliminary report on the trial web-site to notify the coordinating center, within 24 hours. Staff at the coordination centre will work with the site staff to evaluate the event, and to remind site staff of the procedures for diagnosis and management of the event. Coordinating centre staff will continue to monitor the event– as it is investigated and managed.

When the investigation has been finalized (or resolved), the site PI will complete the event final report. This will be reviewed by coordinating center staff (unblinded), to verify if the report is complete, and check that the report is blinded to study regimen, which will then be made available to the panel. For each panel, all three members make independent judgements about the outcomes; if discordant the majority opinion (two out of three) is used, and if all three are discordant, then the panel will re-review.

# Ethical considerations:

In this trial, the major ethical issue is the safety and efficacy of the higher dose shorter regimens. There is reasonable evidence that up to 3 months of higher dose rifampin is safe and well tolerated in patients with active TB disease. There is no reason to expect that serious adverse events will be more frequent in persons with latent TB. However, in these earlier trials the power to detect a significantly greater rate of serious adverse events was limited by small numbers of patients. Hence at least two interim analyses are planned (and more if the DSMB requests this) – to ensure that relatively small differences in rates of adverse events (or poor completion) can be detected and acted upon as soon as possible. All results of interim analyses, and the DSMB’s recommendations will be sent to the IRB.

As well, we consider it plausible that persons with asymptomatic latent TB infection may be less willing to tolerate minor adverse events, such as fatigue, loss of appetite, nausea, or other gastro-intestinal disturbances. We expect the occurrence of these symptoms, even if they do not result in health care providers’ decision to stop therapy, may well result in patients’ decisions to stop therapy early. Although it is normal practice for treating team to encourage patients to obtain full benefit of therapy by completing it, this will be balanced with the need to allow patients to stop therapy without undue pressure because the regimen causes unpleasant symptoms. Hence all study personnel and participating treating teams will be instructed that patients’ decision to stop study therapy, due to poor tolerance, must be respected.

Participants may voluntarily withdraw from the study at any time without prejudice to ongoing or future treatment from the treating team. Management of all participants who decide to withdraw from the study, will be decided by their treating team after discussion with each participant.

All participants will provide signed informed consent, before randomization. For children aged 10-17, we will obtain parental signed consent, and also ask the child to sign assent forms.

If enrollment is occurring during COVID-19 measures, and site PI considers participants can be enrolled at-distance, verbal consent can be given, provided that procedures specified in the section “**Enrolment and follow-up during COVID-19 related measures**” are followed.

This protocol as well as consent and assent forms, will be reviewed by the research ethics board of the McGill University Health Center. After approval by this board, it will be reviewed by research ethics committees/boards at all participating centres.

The Web-based registration system is non-nominal. At the time of registration, all patients are assigned a unique study identification (ID) number. This will be used to label forms, clinical data, and all case report forms. Only non-nominal patient information is entered via the Web-based forms. All patient information sent to the coordinating centre, or reviewed by independent panels, will contain only the study ID.

All contact information including names, address, telephone numbers, other contact persons, and full date of birth will be stored at each site, double locked, in a secure location, and safeguarded by the site PI. Study documents will be stored for 25 years after completion of the study.

In Canada, lists of participants’ names will be forwarded by registered courier to Provincial health authorities to assess if they develop active TB, following procedures approved by provincial privacy commissions. At the international sites the patients’ names will be cross-checked against reported TB cases at State, or National level, using appropriate safeguards for patient confidentiality. Protocols will be written for protection of confidentiality; these will be a focus of training and ongoing monitoring of all sites.

# Study committees

Scientific Advisory Committee (SAC)

The responsibilities of the SAC are to provide advice and recommendations regarding scientific aspects of the studies, particularly study design, interventions and outcomes as well as the statistical analysis plan. We have already formed a SAC for all projects within the CIHR Foundation grant.

Members of the SAC are: Dr. Soumya Swaminathan, Chief Scientist at the World Health Organization in Geneva; Dr. Bill Burman, director of Denver Public Health and long-standing member of TB Trials Consortium; Dr. Andy Vernon, director of Science, TB division, Centre for Disease Control, Atlanta, Georgia and scientific director of TB Trials Consortium; Dr. Ben Marais, professor of University of Melbourne, Australia – considered the global authority on pediatric tuberculosis; Dr. Olivia Oxlade, epidemiologist and modeler at McGill International TB center.

Data Safety and Monitoring Board (DSMB)

The DSMB will be responsible to review the two planned interim analyses, as well as any unexpected Grade 4 adverse events, or deaths that could be related to study regimens. All DSMB recommendations, and the results of the planned interim analyses will be sent to the research ethics committees at all participating sites, and the trial steering committee.

The current DSMB (originally constituted for the 4R vs 9H trial) includes: Dr. Rick O’Brien, chair, formerly director of science at Centre for Disease Control (USA), Dr. Mike Lauzardo, director of the TB centre of Southeast USA (Gainesville, Florida), and Dr. Randall Reeves, Professor of Medicine in Infectious Diseases at the University of Colorado Denver School of Medicine, Denver, USA.

Trial steering committee

The trial steering committee will review progress of the ongoing trial, including enrolment and randomization, pragmatic problems such as difficulties with enrolment or withdrawal of consent, as well as need for study amendments. The trial steering committee will also review recommendations of the SAC and the DSMB. The trial steering committee will be responsible for the final decision regarding stopping enrolment to any study arm, or the study. If such a decision is taken, the research ethics committees at all participating sites will be notified.

This committee will be comprised of the principal investigator (Dr. Dick Menzies), the Foundation project manager (Lisandra Lannes ), the 2R^2^ trial coordinator (Dr. Federica Fregonese), and the trial biostatistician (Dr. Andrea Benedetti) as well as the qualified investigators from each of the six sites.

# References

Aarnoutse, R. E., G. S. Kibiki, K. Reither, H. H. Semvua, F. Haraka, C. M. Mtabho, S. G. Mpagama, J. van den Boogaard, I. M. Sumari-de Boer, C. Magis-Escurra, M. Wattenberg, J. G. M. Logger, L. H. M. Te Brake, M. Hoelscher, S. H. Gillespie, A. Colbers, P. P. J. Phillips, G. Plemper van Balen, M. J. Boeree, and Acea Consortium Pan. 2017. 'Pharmacokinetics, Tolerability, and Bacteriological Response of Rifampin Administered at 600, 900, and 1,200 Milligrams Daily in Patients with Pulmonary Tuberculosis', *Antimicrob Agents Chemother*, 61.

Adhikari, N., and R. Menzies. 1995. 'Community-based tuberculin screening in Montreal: A cost-outcome description', *Am J Public Health*, 85: 786-90.

American Thoracic Society. 2000. 'Diagnostic standards and classification of tuberculosis in adults and children', *Am Rev Resp Dis*, 161: 1376-95.

Anastasatu, C., G. Bungeteanu, and S. Sibila. 1973. 'The intermittent chemotherapy of tuberculosis with rifampicin regimens on ambulatory basis', *Scand J Respir Dis Suppl*, 84: 136-9.

Aspler A, Long R, Trajman A, Dion MJ, Khan K, Schwartzman K, and Menzies D. 2010. 'Impact of treatment completion, intolerance and adverse events on health system costs in a randomized trial of 4 months rifampin or 9 months isoniazid for latent TB', *Thorax*, 65: 582-87.

Blum, Raymond N., Louis B. Polish, Janet M. Tapy, Barbara J. Catlin, and David L. Cohn. 1993. 'Results of screening for tuberculosis in foreign-born persons applying for adjustment of immigration status', *Chest*, 103: 1670-74.

Boeree, M. J., N. Heinrich, R. Aarnoutse, A. H. Diacon, R. Dawson, S. Rehal, G. S. Kibiki, G. Churchyard, I. Sanne, N. E. Ntinginya, L. T. Minja, R. D. Hunt, S. Charalambous, M. Hanekom, H. H. Semvua, S. G. Mpagama, C. Manyama, B. Mtafya, K. Reither, R. S. Wallis, A. Venter, K. Narunsky, A. Mekota, S. Henne, A. Colbers, G. P. van Balen, S. H. Gillespie, P. P. J. Phillips, M. Hoelscher, and Acea consortium Pan. 2017. 'High-dose rifampicin, moxifloxacin, and SQ109 for treating tuberculosis: a multi-arm, multi-stage randomised controlled trial', *Lancet Infect Dis*, 17: 39-49.

Canadian Thoracic Society. 2014. 'Canadian Tuberculosis Standards 7th Edition.', Chapter 6:Treatment of Latent Tuberculosis Infection.

Comstock, GW. 1999. 'How much isoniazid is needed for prevention of tuberculosis in immunocompetent adults', *Int J Tuberc Lung Dis*, 3: 847-50.

Comstock, GW., LM. Hammes, and A. Pio. 1969. 'Isoniazid Prophylaxis in Alaskan Boarding Schools', *Am Rev Resp Dis*, 100: 773-79.

Conde, M. B., F. C. Mello, R. S. Duarte, S. C. Cavalcante, V. Rolla, M. Dalcolmo, C. Loredo, B. Durovni, D. T. Armstrong, A. Efron, G. L. Barnes, M. A. Marzinke, R. M. Savic, K. E. Dooley, S. Cohn, L. H. Moulton, R. E. Chaisson, and S. E. Dorman. 2016. 'A Phase 2 Randomized Trial of a Rifapentine plus Moxifloxacin-Based Regimen for Treatment of Pulmonary Tuberculosis', *PLoS One*, 11: e0154778.

Dasgupta, K., K. Schwartzman, R. Marchand, TN. Tannenbaum, P. Brassard, and D. Menzies. 2000. 'Comparison of cost effectiveness of tuberculosis screening of close contacts and foreign-born populations', *Am J Respir Crit Care Med*, 162: 2079-86.

Dawson, R., K. Narunsky, D. Carman, N. Gupte, A. Whitelaw, A. Efron, G. L. Barnes, J. Hoffman, R. E. Chaisson, H. McIlleron, and S. E. Dorman. 2015. 'Two-stage activity-safety study of daily rifapentine during intensive phase treatment of pulmonary tuberculosis', *Int J Tuberc Lung Dis*, 19: 780-6.

Decroix, G., B. Kreis, C. Sors, J. Birembaum, M. Le Lirzin, and G. Canetti. 1969. '[Comparison between regimes of rifampicin-isoniazid administered daily and administered twice a week (initial results of a comparative study conducted in 4 medical services of the Parisian region)]', *Rev Tuberc Pneumol (Paris)*, 33: 751-68.

Decroix, G., C. Sors, and J. C. Pujet. 1974. '[Complications of intermittent rifampicin treatment. Role of immuno-allergic factors. Study of 8 cases]', *Nouv Presse Med*, 3: 1555-7.

Diacon, A. H., R. F. Patientia, A. Venter, P. D. van Helden, P. J. Smith, H. McIlleron, J. S. Maritz, and P. R. Donald. 2007. 'Early bactericidal activity of high-dose rifampin in patients with pulmonary tuberculosis evidenced by positive sputum smears', *Antimicrob Agents Chemother*, 51: 2994-6.

Diallo, T., M. Adjobimey, R. Ruslami, A. Trajman, O. Sow, J. Obeng Baah, G. B. Marks, R. Long, K. Elwood, D. Zielinski, M. Gninafon, D. A. Wulandari, L. Apriani, C. Valiquette, F. Fregonese, K. Hornby, P. Z. Li, P. C. Hill, K. Schwartzman, A. Benedetti, and D. Menzies. 2018. 'Safety and Side Effects of Rifampin versus Isoniazid in Children', *N Engl J Med*, 379: 454-63.

Dian, S., V. Yunivita, A. R. Ganiem, T. Pramaesya, L. Chaidir, K. Wahyudi, T. H. Achmad, A. Colbers, L. Te Brake, R. van Crevel, R. Ruslami, and R. Aarnoutse. 2018. 'Double-Blind, Randomized, Placebo-Controlled Phase II Dose-Finding Study To Evaluate High-Dose Rifampin for Tuberculous Meningitis', *Antimicrob Agents Chemother*, 62.

Doan, T. N., G. J. Fox, M. T. Meehan, N. Scott, R. Ragonnet, K. Viney, J. M. Trauer, and E. S. McBryde. 2019. 'Cost-effectiveness of 3 months of weekly rifapentine and isoniazid compared with other standard treatment regimens for latent tuberculosis infection: a decision analysis study', *J Antimicrob Chemother*, 74: 218-27.

Dobler, C. C., S. Bosnic-Anticevich, and C. L. Armour. 2018. 'Physicians' perspectives on communication and decision making in clinical encounters for treatment of latent tuberculosis infection', *ERJ Open Res*, 4.

Dorman, S. E., R. M. Savic, S. Goldberg, J. E. Stout, N. Schluger, G. Muzanyi, J. L. Johnson, P. Nahid, E. J. Hecker, C. M. Heilig, L. Bozeman, P. J. Feng, R. N. Moro, W. MacKenzie, K. E. Dooley, E. L. Nuermberger, A. Vernon, M. Weiner, and Consortium Tuberculosis Trials. 2015. 'Daily rifapentine for treatment of pulmonary tuberculosis. A randomized, dose-ranging trial', *Am J Respir Crit Care Med*, 191: 333-43.

Elzi, L., M. Schlegel, R. Weber, B. Hirschel, M. Cavassini, P. Schmid, E. Bernasconi, M. Rickenbach, H. Furrer, and H. I. V. Cohort Study Swiss. 2007. 'Reducing tuberculosis incidence by tuberculin skin testing, preventive treatment, and antiretroviral therapy in an area of low tuberculosis transmission', *Clin Infect Dis*, 44: 94-102.

Fox GJ, Barry SE, Britton WJ, and Marks GB. 2013. 'Contact investigation for tuberculosis: a systematic review and meta-analysis', *Eur Respir J*, 41: 140-56.

Gao, XF., L. Wang, GJ. Liu, J. Wen, X. Sun, Y. Xie, and YP. Li. 2006. 'Rifampicin plus pyrazinamide versus isoniazid for treating latent tuberculosis infection: a meta-analysis', *Int J Tuberc. Lung Dis*, 10: 1080-90.

Geijo, MP., CR. Herranz, P. Vano, AJ. Garcia, M. Carcia, and JF. Dimas. 2007. 'Pauta corta de isoniazida y rifampicina comparada con isoniazida para la infeccion latente de tuberculosis. Ensayo clinica aleatorizado', *Enfermedades Infeccionas Microbiologia Clinica*, 25: 300.

Gordin, FM., JP. Matts, C. Miller, LS. Brown, and et al. 1997. 'A controlled trial of isoniazid in persons with anergy and human immunodeficiency virus infection who are at high risk for tuberculosis', *New Engl J Med*, 337: 315-20.

Hawken, MP., HK. Meme, JM. Chakaya, LC. Elliott, JS. Morris, WA. Githui, ES. Juma, JA. Odhiambo, LN. Thiong’o, JN. Kimari, EN. Ngugi, JJ. Bwayo, CF. Gilks, FA. Plummer, JD. Porter, PP. Nunn, and KP. McAdam. 1997. 'Isoniazid preventive therapy for tuberculosis in HIV-infected adults: results of a randomized controlled trial', *AIDS*, 11: 875-82.

Heemskerk, A. D., N. D. Bang, N. T. Mai, T. T. Chau, N. H. Phu, P. P. Loc, N. V. Chau, T. T. Hien, N. H. Dung, N. T. Lan, N. H. Lan, N. N. Lan, T. Phong le, N. N. Vien, N. Q. Hien, N. T. Yen, D. T. Ha, J. N. Day, M. Caws, L. Merson, T. T. Thinh, M. Wolbers, G. E. Thwaites, and J. J. Farrar. 2016. 'Intensified Antituberculosis Therapy in Adults with Tuberculous Meningitis', *N Engl J Med*, 374: 124-34.

Hong Kong Chest Service Tuberculosis Research Centre, Madras.British Medical Research Council. 1992. 'A double-blind placebo-controlled clinical trial of three antituberculosis chemoprophylaxis regimens in patients with silicosis in Hong Kong', *Am Rev Respir Dis*, 145: 36-41.

International Union Against Tuberculosis Committee on Prophylaxis. 1982. 'Efficacy of various durations of isoniazid preventive therapy for tuberculosis: five years of follow-up in the IUAT trial', *Bull Wld Hlth Org*, 60: 555-64.

Jindani A, Harrison TS, Nunn AJ, Philips PPJ, Churchyard GJ, Charalambous S, Hatherill M, Geldenhuys H, Mclleron HM, Svada SP, Mungofa S, Shah NA, Zizhou S, Magweta L, Shepherd J, Nyirenda s, van Dijk JH, Clouting HE, Coleman D, Bateson ALE, McHugh TD, Butcher PD, and Mitchison DA. 2014. 'High-Dose Rifapentine with Moxifloxacin for Pulmonary Tuberculosis', *N Engl J Med*, 371: 1599-608.

Jindani, A., G. Borgulya, I. W. de Patino, T. Gonzales, R. A. de Fernandes, B. Shrestha, D. Atwine, M. Bonnet, M. Burgos, F. Dubash, N. Patel, A. M. Checkley, T. S. Harrison, D. Mitchison, and St George's University of London International Consortium for Trials of Chemotherapeutic Agents in Tuberculosis. 2016. 'A randomised Phase II trial to evaluate the toxicity of high-dose rifampicin to treat pulmonary tuberculosis', *Int J Tuberc Lung Dis*, 20: 832-8.

Long, MW., Jr.DE. Snider, and LS. Farer. 1979. 'U.S. Public health service cooperative trial of three rifampin-isoniazid regimens in treatment of pulmonary tuberculosis', *Am. Rev. Resp. Dis*, 119: 879-94.

Menzies D, Long R, Trajman A, Dion MJ, Yang J, Al-Jahdali H, Memish ZA, Khan K, Gardam M, Hoeppner VH, Benedetti A, and Schwartzman K. 2008. 'Adverse events with 4 months rifampin or 9 months isoniazid as therapy for latent TB infection: results of a randomized trial.', *Annals of Internal Medicine*, 149: 689-97.

Menzies, D., M. Adjobimey, R. Ruslami, A. Trajman, O. Sow, H. Kim, J. Obeng Baah, G. B. Marks, R. Long, V. Hoeppner, K. Elwood, H. Al-Jahdali, M. Gninafon, L. Apriani, R. C. Koesoemadinata, A. Kritski, V. Rolla, B. Bah, A. Camara, I. Boakye, V. J. Cook, H. Goldberg, C. Valiquette, K. Hornby, M. J. Dion, P. Z. Li, P. C. Hill, K. Schwartzman, and A. Benedetti. 2018. 'Four Months of Rifampin or Nine Months of Isoniazid for Latent Tuberculosis in Adults', *N Engl J Med*, 379: 440-53.

Morrison J, Pai M, and Hopewll PC. 2008. 'Tuberculosis and latent tuberculosis infection in close contacts of people with pulmonary tuberculosis in low-income andmiddle -income countries:a systematic review and meta-analysis', *Lancel Infect Dis*, 8: 368.

Nitti, V. 1973. 'Controlled Clinical Evaluation of Three Intermittent Regimens Employing Low-dosage Schedules of Rifampicin in Original Treatment of Pulmonary Tuberculosis', *Scand. J. Resp. Dis*, supplement: 180-85.

Njie, G. J., S. B. Morris, R. Y. Woodruff, R. N. Moro, A. A. Vernon, and A. S. Borisov. 2018. 'Isoniazid-Rifapentine for Latent Tuberculosis Infection: A Systematic Review and Meta-analysis', *Am J Prev Med*, 55: 244-52.

Onofre Moran-Mendoza, A. 2004. 'The value of the tuberculin skin test size in predicting the development of tuberculosis in contacts of active cases', Department of Health Care and Epidemiology, University of British Columbia.

Pease, C., B. Hutton, F. Yazdi, D. Wolfe, C. Hamel, P. Quach, B. Skidmore, D. Moher, and G. G. Alvarez. 2017. 'Efficacy and completion rates of rifapentine and isoniazid (3HP) compared to other treatment regimens for latent tuberculosis infection: a systematic review with network meta-analyses', *BMC Infect Dis*, 17: 265.

Pocock, S. J., T. C. Clayton, and G. W. Stone. 2015. 'Challenging Issues in Clinical Trial Design: Part 4 of a 4-Part Series on Statistics for Clinical Trials', *J Am Coll Cardiol*, 66: 2886-98.

Ruslami, R., A. R. Ganiem, S. Dian, L. Apriani, T. H. Achmad, A. J. van der Ven, G. Borm, R. E. Aarnoutse, and R. van Crevel. 2013. 'Intensified regimen containing rifampicin and moxifloxacin for tuberculous meningitis: an open-label, randomised controlled phase 2 trail', *Lancet Infect Dis*, 13: 27-35.

Ruslami, R., HJ. Nijland, B. Alisjahbana, I. Parwait, R. van Crevel, and RE. Aarnouste. 2007. 'Pharmacokinetics and tolerability of a higher rifampicin dose versus the standard dose in pulmonary tuberculosis patients', *Antimicrob Agents Chemothe*, 51: 2551.

Saukkonen, J.J., D.L. Cohn, R.M. Jasmer, S. Schenker, J.A. Jereb, C.M. Nolan, C.A. Peloquin, F.M. Gordin, D. Nunes, D.B. Strader, J. Bernardo, R. Venkataramanan, and T.R. Sterling. 2006. 'An official ATS statement: hepatotoxicity of antituberculosis therapy', *Am J Respir Crit Care Med*, 174: 935-52.

Seijger, C., W. Hoefsloot, I. Bergsma-de Guchteneire, L. Te Brake, J. van Ingen, S. Kuipers, R. van Crevel, R. Aarnoutse, M. Boeree, and C. Magis-Escurra. 2019. 'High-dose rifampicin in tuberculosis: Experiences from a Dutch tuberculosis centre', *PLoS One*, 14: e0213718.

Singapore Tuberculosis Service British Medical Research Council. 1975. 'Controlled trial of intermittent regimens of rifampicin plus isoniazid for pulmonary tuberculosis in Singapore', *The Lancet*, 306: 1105-09.

Smieja MJ, Marchetti CA, Cook DJ, and Smaill FM. 2000. 'Isoniazid for preventing tuberculosis in non-HIV infected persons', *Cochrane Database Syst. Rev*: CD001363.

Spyridis NP, Spyridis PG, Gelesme A, Sypsa V, Valianatou M, Metsou F, Gourgiotis D, and Tsolia MN. 2007. 'The Effectiveness of a 9-Month Regimen of Isoniazid Alone versus 3- and 4- Month Regimens of Isoniazid plus Rifampin for Treatment of Latent Tuberculosis Infection in Children: REsults of an 11-Year Randomized Study', *Clinical Infectious Diseases*, 45: 715.

Sterling TR, Moro RN, Borisov AS, Phillips E, Shepherd G, Adkinson NF, Weis S, Ho C, and Villarino ME. 2015. 'Flu-like and Other Systemic Drug Reactions Among Persons Receiving Weekly Rifapentine plus Isoniazid or Daily Isoniazid for Treatment of Latent Tuberculosis Infection in the PREVENt Tuberculosis Study', *Clin Infec Dis*, 61: 527-35.

Svensson, R. J., R. E. Aarnoutse, A. H. Diacon, R. Dawson, S. H. Gillespie, M. J. Boeree, and U. S. H. Simonsson. 2018. 'A Population Pharmacokinetic Model Incorporating Saturable Pharmacokinetics and Autoinduction for High Rifampicin Doses', *Clin Pharmacol Ther*, 103: 674-83.

Swindells, S., R. Ramchandani, A. Gupta, C. A. Benson, J. Leon-Cruz, N. Mwelase, M. A. Jean Juste, J. R. Lama, J. Valencia, A. Omoz-Oarhe, K. Supparatpinyo, G. Masheto, L. Mohapi, R. O. da Silva Escada, S. Mawlana, P. Banda, P. Severe, J. Hakim, C. Kanyama, D. Langat, L. Moran, J. Andersen, C. V. Fletcher, E. Nuermberger, R. E. Chaisson, and Brief Tb A. Study Team. 2019. 'One Month of Rifapentine plus Isoniazid to Prevent HIV-Related Tuberculosis', *N Engl J Med*, 380: 1001-11.

Uplekar, M., D. Weil, K. Lonnroth, E. Jaramillo, C. Lienhardt, HM. Dias, D. Falzon, K. Floyd, G. Gargioni, H. Getahun, C. Gilpin, P. Glaziou, M. Grzemska, F. Mirzayev, H. Nakatani, and M. Raviglione. 2015. 'WHO's new End TB Strategy', *Lancet*, 385: 1799-801.

van Beek, S. W., R. Ter Heine, R. J. Keizer, C. Magis-Escurra, R. E. Aarnoutse, and E. M. Svensson. 2019. 'Personalized Tuberculosis Treatment Through Model-Informed Dosing of Rifampicin', *Clin Pharmacokinet*.

Velasquez, G. E., M. B. Brooks, J. M. Coit, H. Pertinez, D. Vargas Vasquez, E. Sanchez Garavito, R. I. Calderon, J. Jimenez, K. Tintaya, C. A. Peloquin, E. Osso, D. B. Tierney, K. J. Seung, L. Lecca, G. R. Davies, and C. D. Mitnick. 2018. 'Efficacy and Safety of High-Dose Rifampin in Pulmonary Tuberculosis. A Randomized Controlled Trial', *Am J Respir Crit Care Med*, 198: 657-66.

World Health Organization. 2014. "Guidance for national tuberculosis programmes on the management of tuberculosis in children –

2nd ed." In.

———. 2015. "The End TB Strategy." In.

———. 2018a. "Latent TB Infection : Updated and consolidated guidelines for programmatic management." In. <https://www.who.int/tb/publications/2018/latent-tuberculosis-infection/en/>.

———. 2018b. "World leaders commit to bold targets and urgent action to end TB." In.

Yuan, L., E. Richardson, and PRW. Kendall. 1995. 'Evaluation of a tuberculsosis screening program for high-risk students in Toronto schools', *Can Med Assoc J*, 153: 925-32.

Yunivita, V., S. Dian, A. R. Ganiem, E. Hayati, T. Hanggono Achmad, A. Purnama Dewi, M. Teulen, P. Meijerhof-Jager, R. van Crevel, R. Aarnoutse, and R. Ruslami. 2016. 'Pharmacokinetics and safety/tolerability of higher oral and intravenous doses of rifampicin in adult tuberculous meningitis patients', *Int J Antimicrob Agents*, 48: 415-21.

Zenner, D., N. Beer, R. J. Harris, M. C. Lipman, H. R. Stagg, and M. J. van der Werf. 2017. 'Treatment of Latent Tuberculosis Infection: An Updated Network Meta-analysis', *Ann Intern Med*, 167: 248-55.

Zhang, T., M. Zhang, I. M. Rosenthal, J. H. Grosset, and E. L. Nuermberger. 2009. 'Short-course therapy with daily rifapentine in a murine model of latent tuberculosis infection', *Am J Respir Crit Care Med*, 180: 1151-7.

## Appendix 1: Potentially important drug interactions for rifampin.

**NOTE *These lists are not exhaustive. If you are unsure about a specific medication, please contact the coordinating office.*** (note: “adjust levels” in Reccomendations column refers to “adjust level of concomitant medication”)

| **Group** | **Drug class** | **Drug name** | **Effects** | **Recommendations** |
| --- | --- | --- | --- | --- |
| Antiacids | Aluminum hydroxide gel, Magnesium hydroxide gel |  | Antacid reduces RIF absorption | RIF should be taken 1 hour before or 2 hours after antacid |
| Antiarrhythmics | Amiodarone, Digoxin | Aceyldigoxin, Amiodarone, Disopyramide, Mexiletine, Quinidine, Tocainide | RIF decreases levels of antiarrhythmics | Follow and adjust levels |
| Antibiotics |  | Chloramphenicol, Clarithromycin, Doxycycline, Dapsone | RIF decreases levels of antibiotics | Follow and adjust levels |
| Antibiotics |  | Clarithromycin, Erythromycin | These antibiotics increase levels of RIF | Follow and adjust levels |
| Anticoagulants |  | Anisindone, Dicoumarol, Nicoumalone,  Warfarine | RIF decreases levels of anticoagulants | Follow PT, INR |
| Anticonvulsants | Barbiturates, Benzodiazepines, Phenytoin | Diazepam, Lorazepam, etc | RIF decreases levels of anticonvulsants | Follow and adjust levels |
| Antidepressants | Tricylciques | Amitiptyline, Amoxapine, | RIF decreases levels of antidepressants | Follow and adjust levels |
| Antifungal |  | Fluconazole, Itraconazole, Ketoconazole | RIF decreases levels of antifungals, Ketoconazole reduces RIF absorption | Follow and adjust levels, separate ketoconazole and RIF dose by 12 hours |
| Antihypertensive |  | B-blockers (Metoprolol), Calcium-channel blockers (Amlodipine, Verapamil, Diltiazem. ) | RIF decreases levels of antihypertensives | Follow and adjust levels |
| Antimalarials | Quinine |  | RIF decreases levels of Quinine | Follow and adjust levels |
| Antipsychotic | Haloperidol |  | RIF decreases levels of Haloperidol | Follow and adjust levels |

**Potentially important drug interactions with RIF (continued)**

| **Group** | **Drug class** | **Drug name** | **Effects** | **Recommendations** |
| --- | --- | --- | --- | --- |
| Bronchodilatators | Theophyllines | Aminophylline, Diprophylline, Dyphylline, Oxtriphylline, Theophylline, | RIF decreases levels of Theophylline | Follow and adjust levels of theophylline |
| Erectile dysfunction | Sildenafil | Viagra | RIF decreases levels of Sildenafil | Follow and adjust levels |
| General anaesthetics |  |  | RIF decreases levels of General anaesthetics | Follow and adjust levels |
| Hormonal contraceptives |  |  | RIF decreases levels of hormonal contraceptives | Additional barrier method of contraception should be used |
| Immunosuppressive medications |  | Cyclosporine, Tacrolimus, Corticosteroids (prednisone) | RIF decreases levels of immunosuppressive medications | Follow and adjust levels |
| Lipid lowering |  | Clofibrate | RIF decreases levels of Clofibrate | Follow and adjust levels |
| Oral hypoglycemic agents |  | Sulfonylureas, Chlorpropamide, Glimepiride, Glyburide, Glypizide, Tolazanamide, Tolbutamide, Regaglinide | RIF decreases levels of oral hypoglycemic agents | Follow blood sugars and adjust medication |
| Thyroid hormone |  | Levothyroxine | RIF decreases levels of thyroid hormone | Follow and adjust levels |
| Uricosurics | Probenecid |  |  | Follow and adjust levels |

**Potentially important drug interactions with RIF and HIV medication. Note that for all patients placed on RIF, close monitoring is required, including CD4 counts, and virologic response**

Adapted from: AIDS info- **Guidelines for the Use of Antiretroviral Agents in Adults and Adolescents with HIV**

Available at: <https://aidsinfo.nih.gov/guidelines/html/1/adult-and-adolescent-arv/284/pi-drug-interactions>

Last Updated: December 18, 2019; Access date for this document: March 15, 2020

| **Antiretrovirals** | **Recommendations for use with Rifampin** | **Dose adjustment needed (for ART or RIF) and recommendations** | **Additional information on effect on Antiretrovirals and/or Rifampin Drug Concentrations** |
| --- | --- | --- | --- |
| **PIs** |  |  |  |
| All PIs | **Do not use with RIF** | NA | ↓ PI concentration by >75%  Increasing the dose of RTV does not overcome this interaction and may increase hepatotoxicity. Increasing the COBI dose is not recommended. |
| **NNRTIs** |  |  |  |
| DOR | **Do not use with RIF** | NA | DOR AUC ↓ 88% |
| EFV | **Can use with Rifampin** | **EFV dose should be 600mg/day**  **Do not use EFV 400 mg with rifampin.** | EFV AUC ↓ 26% |
| ETR | **Do not use with RIF** | NA | Significant ↓ ETR possible |
| NVP | **Do not use with RIF** | NA | NVP ↓ 20% to 58% |
| RPV | **Do not use with RIF** | NA | RPV AUC ↓ 80% |
| **NRTI** |  |  |  |
| TAF | **Do not use with RIF** **, unless benefits outweigh risks.** | If coadministered, monitor virologic response. | Intracellular TFV-DP levels are higher when TAF is coadministered with rifampin compared to TDF administered alone, but clinical outcomes have not been studied.  **TAF with Rifampin Compared with TDF Alone:**   - TFV-DP AUC ↑ 4.2-fold   **TAF with Rifampin Compared with TAF Alone:**   - TAF AUC ↓ 55% - TFV-DP AUC ↓ 36%   **TAF 25 mg Twice Daily with Rifampin Compared with TAF Once Daily Alone:**   - TAF AUC ↓ 14% - TFV-DP AUC ↓ 24% |
| TDF | **Can use with Rifampin** | No dose adjustment needed. | ↔ AUC TFV |
| **ISTI** |  |  |  |
| BIC | **Do not use with RIF.** |  | BIC AUC ↓ 75% |
| DTG | **Can use with Rifampin-** in patients without suspected or documented INSTI-associated resistance mutations   **Do not use with RIF** in patients with certain suspected or documented INSTI-associated resistance mutations. | Use DTG 50 mg twice daily (instead of DTG 50 mg once daily). | **Rifampin with DTG 50 mg Twice Daily Compared to DTG 50 mg Twice Daily Alone:**   - DTG AUC ↓ 54% and C_min_ ↓ 72%   **Rifampin with DTG 50 mg Twice Daily Compared to DTG 50 mg Once Daily Alone:**   - DTG AUC ↑ 33% and C_min_ ↑ 22% |
| EVG/c | **Do not use with RIF.** | NA | Significant ↓ EVG and COBI expected |
| RAL | **Can use with Rifampin** | Use RAL 800 mg twice daily instead of 400 mg twice daily.  **DO NOT USE RAL 1,200 mg once daily with rifampin**  Monitor closely for virologic response | **RAL 400 mg:**   - RAL AUC ↓ 40% and C_min_ ↓ 61%   **Rifampin with RAL 800 mg Twice Daily Compared to RAL 400 mg Twice Daily Alone:**   - RAL AUC ↑ 27% and C_min_ ↓ 53% |
| **CCR5 Antagonist** |  |  |  |
| MVC | **Can use with Rifampin if Without a Strong CYP3A Inhibitor***  **Do not use with Rifampin If Used With a Strong CYP3A Inhibitor*** | MVC 600 mg twice daily | MVC AUC ↓ 63% |

**Notes**: * examples of CYP3A inhibitors are: EVG/c, All Pis, EFV (reference: Table 20. Mechanisms of Antiretroviral-Associated Drug Interactions; available at: <https://aidsinfo.nih.gov/guidelines/htmltables/1/7346>)

**Abbreviations:** **BIC**: bictegravir; **COBI** or **/c:** cobicistat; **DOR**: doravirine; **DTG** :dolutegravir; **EFV**: efavirenz ; **ETR**: etravirine; **EVG**: elvitegravir; **MVC**: maraviroc; **NVP**: nevirapine; **RAL**: raltegravir; **RPV**: rilpivirine; **TAF**: tenofovir alafenamide; **TDF**: tenofovir disoproxil fumar

## Appendix 2: 2R2 Supplement document: Safety and efficacy of LTBI treatment

**Additional background information related to trial protocol**

Table of Contents of appendix 2

1. Currently recommended regimens for treatment of latent TB 1

2. Safety of currently available TB preventive treatment regimens: 2

6-9 months INH 2

3-4 months INH & Rifampin 3

3 Months INH & Rifapentine (3HP) 4

1 Month isoniazid-rifapentine (1HP) 5

4 Months Rifampin: 5

Comparing Adverse events - with different TPT regimens 8

3. High dose rifampin 9

Evidence for high dose rifampin in treatment of active disease 9

Use of high dose rifapentine for treatment of active TB 12

Use of high dose rifamycins in short latent TB treatment regimens 13

Summary: Use of high dose rifampin for treatment of latent TB 14

Rationale for two months duration: 15

References 17

# 1. Currently recommended regimens for treatment of latent TB

In January 2018 the WHO published updated recommendations for treatment of LTBI (World Health Organization 2018a). In LMIC three regimens are recommended as equivalent options: 6 months of isoniazid (6H), isoniazid and rifampin for 3-4 months (3-4HR), and once-weekly (1/7) isoniazid plus rifapentine for 3 months (3HP). The latter two rifamycin containing regimens have been shown to have superior completion, and non-inferior efficacy compared to 6-9 months INH in randomized trials (Geijo et al. 2007; Hong Kong Chest Service Tuberculosis Research Centre 1992; Spyridis NP et al. 2007), and meta-analyses (Njie et al. 2018; Pease et al. 2017; Zenner et al. 2017) . However, both of these shorter regimens have had similar (3HR, (Ena and Valls 2005) or significantly higher (3HP, (Sterling TR et al. 2015; Sterling TR et al. 2011) rates of Grade 3-4 adverse events than the longer mono-INH. Hence safety remains an issue.

In the same guidelines, WHO recommended 9 months INH (9H) and 4 months Rifampin (4R) as additional and equivalent options for LTBI treatment in countries with TB disease incidence less than 100/100,000. 9H has been estimated to have 90% efficacy for TB prevention (Comstock 1999), which is substantially better than the estimated efficacy of 69% of 6H when compared to placebo in earlier trials (International Union Against Tuberculosis Committee on Prophylaxis 1982). In one placebo-controlled trial, 3 months daily rifampin (3R) had estimated efficacy of 63% (Hong Kong Chest Service Tuberculosis Research Centre 1992), which was non-significantly better than the efficacy of 6H in the same trial. In our recently completed randomized trials in 6800 adults and children, the 4R regimen was slightly (non-significantly) better than 9H for prevention of active TB (Diallo et al. 2018; Menzies et al. 2018) This evidence supported the recommendation of using 4R therapy at standard doses (Canadian Thoracic Society 2014) (American Thoracic Society 2000)).

Unfortunately, 3HP, 3HR and 4R regimens have not been compared head-to-head in trials, although a recent network meta-analysis found that 4R was the safest and most effective regimen of the four options (Zenner et al. 2017). All of these ‘short’ regimens have lower health system costs (Aspler A et al. 2010; Doan et al. 2019), largely related to the reduced number of follow-up visits required. Fewer follow-up visits should result in reduced burden on patients as well. As a result of these trials, and the meta-analysis, 4R and 3HP will become the two preferred regimens for LTBI treatment in forthcoming recommendations by Center for Diseases Control/ National Tuberculosis Controllers Association (Sterling TR In press), and the Canadian Thoracic Society will likely issue similar recommendations in 2019 (study PI, Dr. Menzies, is involved in writing these Standards).

Although the change from 6-9 months INH to the shorter rifamycin-based regimens should result in a significant improvement in LTBI treatment completion, these new “short” regimens are still 3-4 months in duration. This remains challenging as they still result in substantial burden of time and costs – for patients and health system. A reduction in duration to two months would represent substantially less burden for patients and would significantly enhance the feasibility for health system in low and middle income countries of large scale implementation of latent TB treatment – felt to be needed to achieve sustained improvement in the global TB epidemic (World Health Organization 2018b, 2015).

# 2. Safety of currently available TB preventive treatment regimens:

## 6-9 months INH

As seen in Table S1, INH induced hepato-toxicity is well known to be strongly associated with older age. Previous studies have documented incidence of clinically significant toxicity of more than 1% in persons over the age of 35, and more than 5% in those older than 65. Surveillance studies, summarized in Table S2, have identified significant mortality from INH, although the rate of mortality has declined over the years, likely due to better patient selection and better follow-up during therapy

**Table S1: Incidence of clinically significant hepato-toxicity from INH** (Kopanoff, Snider, and Caras 1978; Stead et al. 1985)

| **Age group (in years)** | **Incidence of INH hepato-toxicity** |
| --- | --- |
| 0-20 | <0.1% |
| 20-34 | 0.3% |
| 35-49 | 1.2% |
| 49-64 | 2.3% |
| 65+ | >5% |

**Table S2: Mortality from INH associated liver disease - over the years**

| **Study** | **Years** | **Age Group**  **(in years)** | **Mortality**  **(per 100,000)** |
| --- | --- | --- | --- |
| **USPHS Surveillance** (Kopanoff, Snider, and Caras 1978) | 1971-72 | <35 | 0 |
|  |  | >35 | 98 |
| **IUAT Eastern European trial** (International Union Against Tuberculosis Committee on Prophylaxis 1982) | 1969-72 | 35-65 | 14 |
| **CDC Surveillance** (Snider and Caras 1992) | 1972-73 | All | 54 |
|  | 1974-83 | All | 14 |
|  | 1984-88 | All | 6 |
| **Salpeter 1993** (Salpeter 1993) | 1983-92 | <35 | 0.6 |
|  |  | >35 | 2.4 |

## 3-4 months INH & Rifampin

As shown in Table S3, a meta-analysis of 5 randomized trials (Ena and Valls 2005) found that serious adverse events, most of which were hepato-toxicity, were equal in patients receiving 3 or 4 months of INH and Rifampin daily when compared to regimens of 6 or 12 months of INH. Interestingly in the same analysis they showed the efficacy was also about the same. This regimen is attractive in LMIC settings as the fixed dose combination tablets of INH & Rifampin used for active TB can be used for latent TB. But this meta-analysis showed that the gain of a few months less duration was offset by a similarly unacceptable high rate of liver toxicity.

**Table S3: Serious adverse events with 3-4 months INH & Rifampin vs 6-12 months INH** (Ena and Valls 2005)

| **Author (Country, Population)** | **INH** | **INH&RIF** | **Rate Difference**  **(RIF - INH)** |
| --- | --- | --- | --- |
| BMRC (Hong Kong, Silicosis)(Hong Kong Chest Service Tuberculosis Research Centre 1992) | 13/173 | 8/167 | -2.7% |
| Martinez (Spain, PLHIV)(Martinez Alfaro et al. 1998) | 9/98 | 7/98 | -2.0% |
| **Martinez (Spain, PLHIV)**(Martinez Alfaro et al. 2000) | 15/64 | 5/69 | - 16% |
| **Rivero (Spain, PLHIV)**(Rivero et al. 2003) | 6/83 | 15/82 | + 11% |
| **Whalen (Uganda, PLHIV)**(Whalen et al. 1997) | 3/536 | 13/556 | + 1.7% |
| Pooled estimates | 46/954 | 48/972 | +0.1% |

## 3 Months INH & Rifapentine (3HP)

This regimen has been studied in several large-scale randomized trials and also in observational studies. In the largest trial in HIV-uninfected persons, the efficacy of 3HP was non-inferior to 9-months INH (in fact was almost superior). Subsequent studies have confirmed that the rates of active TB in persons completing the 12-week course of therapy are very low, and experimental studies in non-human primates provide evidence that this is a sterilizing regimen. However, this regimen is also associated with higher rates of adverse events than 9-months INH in randomized trials, whether these are grades 3-4 adverse events or drugs stopped for adverse events (Conde et al.) This is not due to liver toxicity - which is actually substantially and significantly lower. The higher rates of adverse events are due to the excess occurrence of systemic drug reactions as shown in Table S4, and in more detail in Table S5. These systemic drug reactions can result in hospitalization. In the initial large-scale trial (Sterling TR et al. 2015; Sterling TR et al. 2011) there were 4 hospitalizations for this problem in the patients randomized to 3HP. This problem is still not well understood or explained. Risk factors for this problem in the initial study were quite ethnic origin, females, older and at ideal body weight (not a very helpful set of characteristics as this was the most common clinical group in the entire study). However, there have been no deaths associated with this problem or any other adverse events associated with the use of 3HP.

**Table S4: Overall rates of adverse events in a RCT of 3HP vs 9H** (Sterling TR et al. 2011)

|  | **9INH** | **3HP** |
| --- | --- | --- |
| **Randomized, N** | 3649 | 3895 |
| **Total- Grade 3-4 AE (%)** | 7.4% | 6.0% |
| **Drugs stopped for AE(%)** | 3.6% | 5.0% |
| **Hepatotoxicity (%)** | 2.8% | 0.5% |
| **Hypersensitivity (%)** | 0.8% | 4.0% |

**Table S5: Systemic drug reactions, among 7552 persons received at least 1 dose of INH or 3HP** (Sterling TR et al. 2015)

|  | **3HP** | **9INH** | **P Value** |
| --- | --- | --- | --- |
| **Incidence** | | | |
| **Any SDR** | 138 (3.3%) | 15 (0.4)% | <.001 |
| **Severe SDR** | 13 (0.3%) | 1 (<0.1)% | <.001 |
| **Incidence per type of reaction** | | | |
| **Cutaneous (rash)** | 23 (0.5%) | 9 (0.2)% | Ns |
| **Flu-like** | 87 (2.1%) | 2 (<0.1%) | <.001 |
| **Gastro-intestinal** | 7 (0.2%) | 1 (0%) | <.01 |
| **Respiratory** | 6 (0.1%) | 0 (0%) | <.01 |

## 1 Month isoniazid-rifapentine (1HP)

A short treatment of daily self-administered isoniazid and rifapentine taken for one month (1HP) has been recently tested in a phase 3 trial (Swindells et al. 2019). The multicenter trial, which enrolled 3000 adult participants with HIV, found that the regimen had equal efficacy and similar toxicity as daily self-administered 9-months INH. Completion, based on self-report at monthly visits by participants, was 90% in the 9INH arm compared to 97% in the 1HP arm. These rather remarkable rates of completion were accompanied by similar rates of efficacy and toxicity. The interesting findings of this study must be confirmed, in other trials in people living with HIV and also in non-HIV-infected adults and children.

## 4 Months Rifampin:

This regimen has been studied extensively. In observational studies, completion rates have been superior to comparator regimens of 6 to 9 months INH, and adverse events, particularly hepato-toxicity, significantly lower with 4-months Rifampin. In a population-based study of 19,773 patients receiving 6 to 9 months INH or 4-months Rifampin, the rates of severe hepato-toxicity were substantially lower with 4RIF and only occurred in elderly people with comorbidities, as shown in table S6 (Ronald LA In press).

**Table S6: Comparison of number and rate of severe hepato-toxicity in Quebec: 1998-2007. A population -based study** (Ronald LA In press).

|  | **With other co-morbidities,**  **N(rate)** | | | | **Without other co-morbidities,**  **N(rate)** | | | | **Total** |
| --- | --- | --- | --- | --- | --- | --- | --- | --- | --- |
| **Age (in years)** | **0-19** | **20-34** | **35-64** | **≥65** | **0-19** | **20-34** | **35-64** | **≥65** | **All ages** |
| **4 RIF** | 0 | 0 | 0 | 1 (0.9) | 0 | 0 | 0 | 0 | 1 (0.1) |
| **6-9 INH** | 0 | 1 (0.4) | 2 (0.6) | 2 (1.1) | 2 (0.1) | 2 (0.1) | 5 (0.2) | 1 (0.2) | 15 (0.2) |

In three randomized trials (Diallo et al. 2018; Menzies D et al. 2008; Menzies et al. 2018), two in adults and one in children but all from the same group, the occurrence of all Grade 3-4 adverse events judged related to therapy and resulting in permanent cessation of study drug was significantly lower with 4RIF compared to 9-months INH. In particular, grade 3-4 hepato-toxicity was substantially and significantly lower with a rate of 3 per 1,000 in those randomized to 4RIF, compared to 20 per 1,000 (2%) in those randomized to 9INH. In children, 4RIF was well tolerated (as was 9INH); there were no serious drug-related adverse events at all. Minor adverse events and symptoms were similar in children taking 4RIF or 9-months INH.

**Table S7: Occurrence of Adverse events in 3 randomized trials of 4RIF vs 9INH** (Diallo et al. 2018; Menzies D et al. 2008; Menzies et al. 2018)

|  | **4RIF** | **9INH** | **Risk Difference**  **(4RIF-9INH, %)** |
| --- | --- | --- | --- |
| **Results in Adults (18+)** |  |  |  |
| **Total Number treated** | N=3280 | N=3205 |  |
| **Treatment stopped for AE (All), n (%)** | 93 (2.8) | 179 (5.6) | -2.9 (-3.9, -1.9) |
| **Treatment stopped due to AE Judged Related to Therapy. n (%)** | 68 (2.1) | 131 (4.1) | -2.0 (-2.9, -1.2) |
| **Grade 1-2, n (%)** | 37 (1.1) | 56 (1.7) | -0.6 (-1.2, -0.03) |
| **Grade 3-4, n (%)** | 31 (0.9) | 74 (2.3) | -1.4 (-2.0, -0.8) |
| **Grade 5 (Death), n (%)** | 0 | 1 (0.03) | -0.03 (-0.1, 0.03) |
| **Adverse Event Type (in adults)**  **(Judged Related to Therapy)** |  |  |  |
| **Grade 3-4 Hepatotoxicity, n (%)** | 11 (0.3) | 65 (2.0) | -1.7 (-2.2, -1.2) |
| **Grade 1-4 Rash, n (%)** | 25 (0.8) | 13 (0.4) | 0.4 (-0.01, 0.7) |
| **Grade 3-4 Hematologic, n (%)** | 6 (0.2) | 0 | 0.2 (0.04, 0.3) |
| **Grade 3-5 Other, n (%)** | 8 (0.2) | 8 (0.2) | 0 (-0.3, 0.2) |
| **Results in children (0-17)** |  |  |  |
| **Total Number treated** | N=422 | N=407 |  |
| **Serious Adverse Event, n** | 1 (death) | 1 (pregnancy) | 0 |
| **Serious adverse events attributed to treatment** | 0 | 0 | 0 |
| **Did not have any follow-up visits (N, %)** | 26 (6.2) | 25 (6.1) |  |
| **Seen at least once in follow-up (N, %)** | 396 (93.8) | 382 (93.9) |  |
| **Average (STD) % of visits during follow-up when any minor symptom was reported** | 8.1 (20.3) | 8.5 (18.9) | -0.3% (-3.3, 2.7) |
| **Average (STD) % of visits during follow-up when a minor symptom that may have been related to the study drugs was reported** | 4.4 (14.5) | 4.2 (11.1) | 0.3% (-1.7, 2.4) |

## Comparing Adverse events - with different TPT regimens

As shown in table S8, 3 major landmark trials published in the past decade have used the same comparator regimen of 9-months INH. In these 3 trials, the incidence of adverse events varied, as might be expected given the differences in the patient populations, and settings, as well as study procedures. However, the incidence of hepato-toxicity was virtually identical in all three trials with 9-months INH, suggesting the AE rates may, in fact, be directly comparable. Hepato-toxicity was uncommon in those taking 3HP or 4RIF (and very similar rates) but higher in those taking 1-month isoniazid rifapentine (see below). On the other hand, other adverse events and total adverse events were significantly lower **only with 4-months Rifampin** compared to 9-months INH. Hence 4R appears to be the safest regimen of all currently available treatment for Latent TB.

**Table S8: Summary - comparing safety of 3 regimens: 4R vs 9H, 3HP vs 9H and 1HP vs 9H**

| **Author, year** | **Sterling et al. 2011** | | **Menzies et al. 2018** | | **Swindells et al. 2019** | |
| --- | --- | --- | --- | --- | --- | --- |
| **Event** | **9H** | **3HP** | **9H** | **4R** | **9H** | **1HP** |
| Drug stopped for adverse events | 3.7% | 4.9% | 3.7% | 1.6% | 2% | 1% |
| Adverse events possibly drug related | 5.5% | 8.2% | 2.3%§ | 0.9%§ | NR | NR |
| Grade 3-4 Hepatotoxicity | 2.7% | 0.4% | 2.9% | 0.3% | 3%^#^ | 2%^#^ |

**Note**: **#** Adverse events in this trial were measured for up to a year post end of treatment.

Table S9, summarizes the major findings of a network meta-analysis of 57 studies, which compared safety and efficacy of commonly used regimens for latent TB (Zenner et al. 2017). In this analysis the regimens of 6-months INH and 9-months INH had lowest efficacy and highest hepato-toxicity, whereas 3HP and 4R were ranked best with lowest risk of hepato-toxicity (meaning least likely to cause liver toxicity) and highest efficacy. Note that in this independent evaluation, 4R was overall ranked #1.

**Table S9: Results of a network meta-analysis, comparing safety and efficacy of 5 commonly used regimens for latent TB (Zenner et al. 2017)**

| **Regimen** | **Incidence of TB (efficacy)** | | **Hepato-toxicity** | |
| --- | --- | --- | --- | --- |
|  | **OR (vs no treatment)** | **Rank**  **(Best=1)** | **OR (vs no treatment)** | **Rank (Best=1)** |
| **6 INH** | 0.4 (0.3, 0.6) | 4 | 1.1 (0.4, 3.2) | 4 |
| **9 INH** | 0.5 (0.2, 0.95) | 5 | 1.7 (0.4, 8.1) | 5 |
| **3-4 HR** | 0.3 (0.2, 0.5) | 2 | 0.7 (0.2, 2.4) | 3 |
| **3HP** | 0.4 (0.2, 0.7) | 3 | 0.5 (0.1, 2.2) | 2 |
| **4R** | 0.3 (0.1, 0.6) | 1 | 0.1 (0.1, 0.8) | 1 |

# 3. High dose rifampin

## Evidence for high dose rifampin in treatment of active disease

The current dose of rifampin for treatment of active disease and latent infection is 10mg/kg per day with a maximum of 600 mg per day. This dose was established in multiple randomized trials conducted in the 1960s and 1970s. In retrospect, the dose selected was the lowest possible effective dose - to minimize adverse events and costs, as rifampin was very expensive at that time, and safety was uncertain. As shown in Table S10, several other studies, also conducted in the 70s, assessed higher doses of rifampin, although in most of these studies the higher doses of rifampin were given once or twice per week. In these studies once-weekly rifampin was associated with unacceptably high toxicity, primarily due to systematic drug reactions (a “flu-like syndrome”). The once-weekly high dose regimens were abandoned. The twice weekly high dose achieved similar efficacy as daily standard doses, and interest in high dose rifampin faded.

**TABLE S10: Incidence of serious adverse events with higher doses of rifampin for treatment of active TB**.

| **Author**  **(Year)** | **Rifampin Doses: Total in mg/day (mg/kg/day) Days/week** | **Duration of high doses** | **AE Definition** | **N Treated** | **Overall AE (N, %)** | **Hepatic AE (N, %)** | **SDR (flu-like) AE (N, %)** |
| --- | --- | --- | --- | --- | --- | --- | --- |
| **Studies on pulmonary TB- intermitted treatment** |  |  |  |  |  |  |  |
| Decroix (1969)(Decroix et al. 1969) | 900 (15) 2/7  600 (10) 7/7 | 6 mos | ns  ns | 32  39 | 0 (-)  3 (8%) | 0 (-)  2 (5%) | NR  NR |
| Anastasatu (1973)(Anastasatu, Bungeteanu, and Sibila 1973) | 1200 (15) 2/7  900 (10) 2/7 | 6 mos | ns  ns | 53  49 | 1 (2%)  3 (6%) | NR  NR | NR  NR |
| Nitti (1973)(Nitti 1973) | 900 (15) 2/7  600 (10) 7/7 | 6 mos | ns  ns | 22  23 | 0 (-)  2 (9%) | 0 (-)  0 (-) | 0 (-)  1 (4%) |
| Decroix (1974)(Decroix, Sors, and Pujet 1974) | 900 (15) 2/7  600 (10) 7/7 | 6 mos | ns  ns | 53  49 | 1 (2%)  3 (6%) | 1 (2%)  3 (6%) | NR  NR |
| Singapore TB Service (1975)(Singapore Tuberculosis Service British Medical Research Council 1975) | 900 (15) 2/7  600 (10) 2/7 | 6 mos | ns  ns | 115  119 | 13 (11%)  8 (7%) | 1 (1%)  0 (-) | 9 (8%)  5 (4%) |
| **Studies on pulmonary TB- daily treatment** |  |  |  |  |  |  |  |
| Long (1979)(Long, Snider, and Farer 1979) | 750 (12.5) 7/7  600 (10) 7/7 | 6 mos | ns  ns | 331  324 | 13 (4%)  13 (4%) | 5 (2%)  5 (2%) | NR  NR |
| Ruslami (2007)(Ruslami et al. 2007) | 600 (13.5) 7/7  450 (10) 7/7 | 2 weeks | ns  ns | 23  24 | 1 (4%)  3 (13%) | 1 (4%)  3 (13%) | 0 (-)  0 (-) |
| Diacon (2007) | 1200 (20) daily | 5 days | ns | 13 | NR | NR | NR |
| Jindani(2016)(Jindani et al. 2016) | 1200 (20) 7/7  900(15) 7/7  600(10) 7/7 | 16 weeks | Grade 3 hep, all AE | 100  100  100 | 13 (13%)  22(22%)  20 (20%) | 4 (4%)  2 (2%)  1 (1%) | NR |
| Aarnoutse (2017)(Aarnoutse et al. 2017) | 1200 (20) 7/7  900 (15) 7/7  600 (10) 7/7 | 2 weeks | Grade 3-5 CTC criteria | 50  50  50 | 5 (10%)  1 (2%)  5 (10%) | 2 (4%)  0 (-)  1 (2%) | NR  NR  NR |
| Boeree (2017)(Boeree et al. 2017) | 1950 (35) 7/7  1200 (20) 7/7  600 (10) 7/7 | 12 weeks | Serious AE, Grade 3-4 Hep. | 63  63  123 | 4 (6%)  4 (6%)  6 (5%) | 3 (5%)  0 (-)  2 (2%) | NR  NR  NR |
| Velasquez (2018) (Velasquez et al. 2018) | 975 (20) 7/7  900 (15) 7/7  450 (10) 7/7 | 8 weeks | AE 2-5 R-related | 60  60  60 | 2 (3.%)  1 (2%)  1 (2%) | 14 (23%)  14(23%)  16 (26%) | NR  NR  NR |

**Abbreviations**: AE: Adverse events; SDR: systemic drug reaction; Hep: hepatotoxicity.

**TABLE S10- Continuation**

| **Author**  **(Year)** | **Rifampin Doses: Total in mg/day (mg/kg/day) Days/week** | **Duration of high doses** | **AE Definition** | **N Treated** | **Overall AE (N, %)** | **Hepatic AE (N, %)** | **SDR (flu-like) AE (N, %)** |
| --- | --- | --- | --- | --- | --- | --- | --- |
| **Studies on TB meningitis- daily treatment** |  |  |  |  |  |  |  |
| Ruslami (2013)(Ruslami et al. 2013) | 600 (13) 7/7  450 (10) 7/7 | 2 weeks | Grade 3-4 | 29  31 | 7 (24%)  5 (16%) | 6 (21%)  5 (16%) | 0 (0%)  0 (0%) |
| Yunivita(2016)(Yunivita et al. 2016) | 750 (17) 7/7  900 (20) 7/7  600 (13) 7/7 | 2 weeks | All grades;  grade 4 Hep | 11  9  10 | 7 (64%)  7(78%)  4 (40%) | 0  1 (11%)  0 | 0(0%)  0(0%)  0(0%) |
| Heemskerk(2016) (Heemskerk et al. 2016) | 900 (15) 7/7  600 (10) 7/7 | 8 weeks | All grades | 408  409 | 240 (59%)  229(56%) | 17(4%)  28 (7%) | NR |
| Dian (2018)(Dian et al. 2018) | 1350 (30) 7/7  900 (20) 7/7  450 (10) 7/7 | 30 days | Grade 3-4 | 20  20  20 | 4 (20%)  8 (40%)  3 (15%) | 4 (20%)  5 (25%)  3 (15%) | NR  NR  NR |

However, there has been renewed interest in use of high dose rifampin to improve outcomes in treatment of severe forms of active TB, specifically TB meningitis; and to reduce duration of treatment of pulmonary TB. In treatment of TB meningitis doses as high as 30mg/kg have been associated with better bacteriologic results and, in one study, reduced mortality(Dian et al. 2018; Ruslami et al. 2013; Yunivita et al. 2016). These studies enrolled small numbers of patients but reported excellent tolerability of the daily higher dose rifampin, although the evidence for long term safety over the entire treatment course is limited as the higher doses of rifampin were given for only 2-4 weeks in these studies.

In a recently published retrospective study (Seijger et al. 2019) , 26 patients with severe TB illness (CNS or pulmonary) were treated with high dose rifampin (900mg/day to 1800mg/day) for 6 to 12 months. Adverse events for these patients were reported together with another 57 patients who were treated with high dose rifampin for low plasma concentrations; higher dose rifampin was well tolerated by all patients in this study.

To date, there have been no published phase 3 clinical trials of high dose rifampin in treatment of active pulmonary TB. From the phase 2 trials, in which doses of rifampin used were between 12.5mg/kg/day and 35mg/kg/day, there has been no evidence of excess toxicity (Boeree et al. 2017; Diacon et al. 2007; Jindani et al. 2016; Ruslami et al. 2007; Velasquez et al. 2018). In these studies, the high dose rifampin was given for 2 weeks up to 3 months (table S10). Sample sizes were small, limiting power to detect less common events such as serious AE.

The primary objective of these phase 2 studies was to detect differences in safety; however, they showed early bactericidal activity (Diacon et al. 2007) and more rapid sputum culture conversion (Jindani et al. 2016; Svensson et al. 2018). These outcomes are considered surrogates for end-of-treatment outcomes; hence these results suggest that shortening of treatment of active pulmonary TB may be achieved with higher doses of rifampin.

Several phase 3 trials, in which doses of rifampin between 20 and 35mg/kg/day are used to shorted the treatment of pulmonary TB, are ongoing (examples are RIFASHORT, SAEFRIF, TRUNCATE TB trial, registered in Clinicaltrials.gov.

## Use of high dose rifapentine for treatment of active TB

Rifapentine is a drug that has similar anti-mycobacterial activity as rifampin, but has a prolonged half-life – up to 5 times longer than that of rifampin. Therefore, to achieve higher exposure to rifapentine – dosing can be once weekly with a higher dose such as 1200 mg instead of the usual 600 mg once weekly, or doses are given daily (at 7.5mg/kg, 10mg/kg, 15mg/kg, or 20mg/kg). Daily dosing result in serum drug levels that increase over the first two weeks and achieve steady state serum levels equivalent to the serum levels achieved with triple the daily standard doses of rifampin. Recent trials have assessed rifapentine 1200mg once a week (Jindani A et al. 2014) or 600mg daily (Conde et al. 2016; Dawson et al. 2015; Dorman et al. 2015). In these studies much higher serum drug levels were achieved with these higher doses, resulting in more rapid sputum sterilization or other favorable treatment outcomes. However, these were phase-2 studies, with limited power to detect efficacy differences.

**Table S11: Safety of high dose Rifapentine* in treatment of pulmonary TB**

| **Study** | **Rifapentine** daily dose (mg/kg), days/week** | **Duration of high dose** | **N** | **Overall safety** |
| --- | --- | --- | --- | --- |
| Jindani et al, 2014 (RIFAQUIN) | 20 1/7  15 2/7  10 7/7 | 4m  2m  6m | 277  275  275 | AE related to study drug similar in the 3 arms |
| Conde et al, 2016 | 7.5 7/7  RIF 10 7/7 | 8w  8w | 62  59 | Similar grade 3; higher discontinuation in intervention arm |
| Dorman et al,  2015 | 20 7/7  15 7/7  10 7/7  RIF 10 7/7 | 8w  8w  8w  8w | 87  81  81  85 | Similar proportion of discontinuation and SAE. |
| Dawson et al, 2015 | 10 7/7  7.5 7/7  RIF 10 7/7 | 8w  8w  8w | 51  54  48 | Similar proportion of discontinuations and Grade 3-5 AE |

**Notes**:

* Rifapentine was given in association with other anti-TB drugs, which varied by study.

** If Rifampin was given in one arm, it is reported as RIF, followed by the daily dose.

## Use of high dose rifamycins in short latent TB treatment regimens

There are number of converging lines of evidence that suggest that high dose Rifampin may allow treatment shortening in active TB and therefore in latent TB as well. First, it should be understood that the current dose of Rifampin used is the lowest dose which achieved reasonable results in early randomized trials conducted in the 1960s. The current dose was selected in part to minimize toxicity, but also to minimize cost as Rifampin in the early 1970s was very expensive. Studies of therapeutic drug monitoring have revealed that Rifampin levels are low in as many as half of all patients routinely treated, and there was evidence that these low drug levels are associated with increased risk of relapse and other adverse treatment outcomes.

Mouse Models - Concurrent with studies on use of high dose rifampin for treatment of active TB, there have been efforts to assess the activity of high dose rifamycins in treatment of latent TB infection. In a mouse-model of latent TB infection, daily dosing of rifapentine has been shown to result in relapse-free cure of latent infection after as little as one month of treatment (Zhang et al. 2009). Notably, in these mouse studies rifapentine was given at the same doses ***daily*** (on a mg/kg basis) as were used when rifapentine was given weekly in previous studies. As explained before, because rifapentine has a prolonged half-life this means that daily dosing will result in serum drug levels equivalent to higher dose of rifampin. In particular, mouse study showed that the total drug AUC (of the 0-168h after drug assumption) values for RPT were 2.5 times higher than for RIF at a given dose, so that a daily intake of 7.5mg/kg of RPT was had similar values of 20mg/kg.day of Rifampin. The C_max_ values were 1.4 times higher for RPT, so a daily intake of 7.5mg of RPT gave similar values of 10mg/kg of rifampin (Rosenthal et al. 2012).

**Table S12. Simulated steady-state PK parameters for rifampim and rifapentine in BALB/c mice, adapted from (Rosenthal et al. 2012)**

| **Regimen** | **PK parameter for total drug** | | | |
| --- | --- | --- | --- | --- |
| **Rifampin** | **C_max_** | **t_1/2_** | **AUC_0–24_** | **AUC_0–168_** |
| 10mg | 13.52 | 2.35 | 142.1 | 711 |
| 15mg | 20.27 | 2.35 | 213.2 | 1,066 |
| 20mg | 27.03 | 2.35 | 284.2 | 1,421 |
| 40mg | 54.06 | 2.35 | 568.4 | 2,842 |
| **Rifapentine** |  |  |  |  |
| 5mg | 9.28 | 18.77 | 179.8 | 920 |
| 7.5mg | 13.93 | 18.77 | 269.7 | 1,380 |
| 10mg | 18.57 | 18.77 | 359.6 | 1,840 |
| 20mg | 37.14 | 18.77 | 719.2 | 3,681 |

Trials in humans of shorter treatment of latent TB - A trial of one month of daily INH (standard doses) and daily Rifapentine (same doses as when given weekly – which should result in much higher serum levels of RPT – as in the mouse model) was recently published (Swindells et al. 2019). In this study an “ultra short” regimen of daily self-administered INH and rifapentine for one month (1HP) was compared to 9 months daily INH in HIV infected adults with presumed latent TB. The 1HP regimen had non-inferior efficacy. There were a number of important methodologic issues with this trial – most notably that latent TB was not confirmed in 80% of participants, so the non-inferior result for TB prevention may simply reflect the fact that the majority of those treated were not at risk for disease. Nevertheless, these interesting results suggest that it may be possible to successfully shorten duration of latent TB therapy without impacting efficacy by achieving higher serum levels of a rifamycin. These preliminary results also suggest that higher doses of a rifamycin can be given safely.

## Summary: Use of high dose rifampin for treatment of latent TB

Our rationale for the trial is based on the following: 1) acceptance, completion and costs of latent TB therapy are superior with shorter regimens; 2) therapy must be safe, and the current safest regimen appears to be 4R; 3) the best completion rate, which has been seen in study conditions with 4R, is still less than 80%; 4) in treatment of active TB, dose rifampin of 20mg/kg.day or higher achieves faster culture conversion; 5) in a mouse model higher rifamycin levels achieve faster clearance of latent TB infection; 6) a recent study achieved promising results with treatment of latent TB with one month of high dose Rifapentine paired with INH.

Taken together, the available evidence suggests that a shorter regimen of high dose daily rifampin may be as effective as the standard 4R. However, there is insufficient evidence to determine the optimal dose that will achieve similar efficacy as 4R, without any increase in adverse events. As a first step, in a phase 2 trial, we plan to verify that high dose rifampin monotherapy is safe, as treatment of latent TB implies treatment of otherwise healthy persons. Our concern about toxicity and motivation to assess this carefully in a phase 2 trial is based on the experience with the 2-month rifampin-pyrazinamide regimen (2RZ). In the 1990s a series of randomized trials, conducted exclusively in HIV infected populations, demonstrated non-inferior efficacy of 2RZ compared to the standard of 9-12 months INH. In these HIV infected study populations, the 2RZ regimen appeared very safe – with similar rates of hepatotoxicity as the INH regimens. Hence 2RZ was strongly recommended in guidelines in the US and Canada in 2000. But when this regimen was subsequently introduced widely in HIV uninfected adults the results were disastrous - with high rates of hepatotoxicity and even deaths due to this regimen. This regimen was abandoned, but the ‘damage’ to TB prevention was done, and many doctors, already concerned about the toxicity of INH, simply do not prescribe LTBI treatment, for fear of side-effects (Adhikari and Menzies 1995; Blum et al. 1993; Dasgupta et al. 2000; Dobler, Bosnic-Anticevich, and Armour 2018; Elzi et al. 2007; Onofre Moran-Mendoza 2004; Yuan, Richardson, and Kendall 1995)

Hence, any regimen that looks promising for TB prevention must be tested carefully for safety and tolerability in the same populations who are likely to receive this – meaning both HIV infected and uninfected, and all ages (adults and children). Rifapentine based regimens have a number of drawbacks, including concerns regarding safety due to potentially severe systemic reactions (Sterling TR et al. 2015), higher costs, and poor accessibility as this drug is not licensed in many countries. This also means providers are inexperienced with this drug, further slowing uptake. Rifampin has the advantage of being already licensed and used widely in all countries so providers are familiar with the drug, plus it has lower cost, and an excellent safety record (at standard dose). Doubling the standard dose (20mg/kg/day) to date has an excellent safety profile, albeit in small studies of patients with serious forms of disease (Boeree et al. 2017; Dian et al. 2018; Jindani et al. 2016; Yunivita et al. 2016). There is less experience with the higher doses (30mg/kg/day or more), but in studies published to date these doses have also been well-tolerated (Boeree et al. 2017; Dian et al. 2018).

Published results suggest that higher doses of rifampin or rifapentine for shorter duration may be effective for TB prevention. Hence we plan to assess the treatment of latent TB with high-dose rifampin for 2 months using a dose of 20 or 30 mg/kg (2R_20_ or 2R_30_). As noted above, standard dose Rifampin has very low rates of liver toxicity and appears to be the safest TPT regimen. Hence, it is reasonable to assume that double dose Rifampin and even triple dose Rifampin should be safe, and it is also plausible that it will be more effective, although this remains to be confirmed. But higher doses of rifampin may increase risk of adverse events. Currently the 4R_10_ regimen is the **only** LTBI regimen with significantly lower rate of Grade 3-4 AE than the comparator arm of 9H in published randomized trials. This is a very important advantage, and it is crucial that any potential gain in completion from a shorter duration must not be offset by worse safety and tolerability.

## Rationale for two months duration:

In one earlier trial, conducted in Hong Kong among men with positive tuberculin skin tests and pulmonary silicosis, 3-months Rifampin (10 mg/kg/day) alone was the most efficacious with substantially lower rates of active TB in the 5 years of follow-up than 6-months of INH. This suggests that 3 months of standard dose Rifampin may be adequate (rather than 4 months which has been tested). Hence 2 months is not that much of a reduction in duration from a regimen with known efficacy. The advantage of a 2-month regimen is simply that of improved completion, and diminished burden of both patients and health systems in terms of the need for visits. As seen in Figure S1, adherence falls off steadily with every successive month with 4RIF and 9INH. Hence it can be expected that a 2-month regimen should have substantially and significantly better completion than a 4-month regimen.

This is the rationale for selecting a 2-month regimen of higher dose Rifampin. We are testing both doses, with double and triple doses because at this time, we are unsure both of safety and of efficacy. Our plan is to extend our study to a full phase study to test efficacy using the single dose that is best tolerated. If the higher dose is well tolerated, and there is no increased occurrence of adverse events, then we will use the higher dose as this theoretically at least is more likely to be efficacious.

**Figure S1: Dropouts from LTBI treatment, stratified by starting regimen (INH vs RMP), Quebec, stratified by periods of treatment start years (N=****19,773) (Ronald LA In press)**


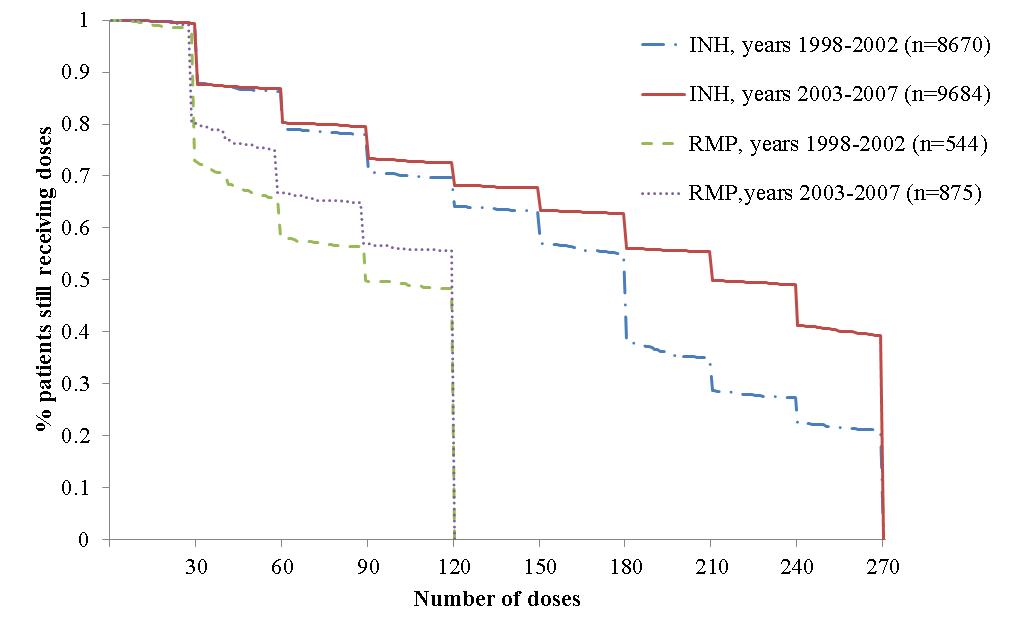


INH=isoniazid; RMP=rifampin

# References for appendix 2

Aarnoutse, R. E., G. S. Kibiki, K. Reither, H. H. Semvua, F. Haraka, C. M. Mtabho, S. G. Mpagama, J. van den Boogaard, I. M. Sumari-de Boer, C. Magis-Escurra, M. Wattenberg, J. G. M. Logger, L. H. M. Te Brake, M. Hoelscher, S. H. Gillespie, A. Colbers, P. P. J. Phillips, G. Plemper van Balen, M. J. Boeree, and Acea Consortium Pan. 2017. 'Pharmacokinetics, Tolerability, and Bacteriological Response of Rifampin Administered at 600, 900, and 1,200 Milligrams Daily in Patients with Pulmonary Tuberculosis', *Antimicrob Agents Chemother*, 61.

Adhikari, N., and R. Menzies. 1995. 'Community-based tuberculin screening in Montreal: A cost-outcome description', *Am J Public Health*, 85: 786-90.

American Thoracic Society. 2000. 'Diagnostic standards and classification of tuberculosis in adults and children', *Am Rev Resp Dis*, 161: 1376-95.

Anastasatu, C., G. Bungeteanu, and S. Sibila. 1973. 'The intermittent chemotherapy of tuberculosis with rifampicin regimens on ambulatory basis', *Scand J Respir Dis Suppl*, 84: 136-9.

Aspler A, Long R, Trajman A, Dion MJ, Khan K, Schwartzman K, and Menzies D. 2010. 'Impact of treatment completion, intolerance and adverse events on health system costs in a randomized trial of 4 months rifampin or 9 months isoniazid for latent TB', *Thorax*, 65: 582-87.

Blum, Raymond N., Louis B. Polish, Janet M. Tapy, Barbara J. Catlin, and David L. Cohn. 1993. 'Results of screening for tuberculosis in foreign-born persons applying for adjustment of immigration status', *Chest*, 103: 1670-74.

Boeree, M. J., N. Heinrich, R. Aarnoutse, A. H. Diacon, R. Dawson, S. Rehal, G. S. Kibiki, G. Churchyard, I. Sanne, N. E. Ntinginya, L. T. Minja, R. D. Hunt, S. Charalambous, M. Hanekom, H. H. Semvua, S. G. Mpagama, C. Manyama, B. Mtafya, K. Reither, R. S. Wallis, A. Venter, K. Narunsky, A. Mekota, S. Henne, A. Colbers, G. P. van Balen, S. H. Gillespie, P. P. J. Phillips, M. Hoelscher, and Acea consortium Pan. 2017. 'High-dose rifampicin, moxifloxacin, and SQ109 for treating tuberculosis: a multi-arm, multi-stage randomised controlled trial', *Lancet Infect Dis*, 17: 39-49.

Canadian Thoracic Society. 2014. 'Canadian Tuberculosis Standards 7th Edition.', Chapter 6:Treatment of Latent Tuberculosis Infection.

Comstock, GW. 1999. 'How much isoniazid is needed for prevention of tuberculosis in immunocompetent adults', *Int J Tuberc Lung Dis*, 3: 847-50.

Comstock, GW., LM. Hammes, and A. Pio. 1969. 'Isoniazid Prophylaxis in Alaskan Boarding Schools', *Am Rev Resp Dis*, 100: 773-79.

Conde, M. B., F. C. Mello, R. S. Duarte, S. C. Cavalcante, V. Rolla, M. Dalcolmo, C. Loredo, B. Durovni, D. T. Armstrong, A. Efron, G. L. Barnes, M. A. Marzinke, R. M. Savic, K. E. Dooley, S. Cohn, L. H. Moulton, R. E. Chaisson, and S. E. Dorman. 2016. 'A Phase 2 Randomized Trial of a Rifapentine plus Moxifloxacin-Based Regimen for Treatment of Pulmonary Tuberculosis', *PLoS One*, 11: e0154778.

Dasgupta, K., K. Schwartzman, R. Marchand, TN. Tannenbaum, P. Brassard, and D. Menzies. 2000. 'Comparison of cost effectiveness of tuberculosis screening of close contacts and foreign-born populations', *Am J Respir Crit Care Med*, 162: 2079-86.

Dawson, R., K. Narunsky, D. Carman, N. Gupte, A. Whitelaw, A. Efron, G. L. Barnes, J. Hoffman, R. E. Chaisson, H. McIlleron, and S. E. Dorman. 2015. 'Two-stage activity-safety study of daily rifapentine during intensive phase treatment of pulmonary tuberculosis', *Int J Tuberc Lung Dis*, 19: 780-6.

Decroix, G., B. Kreis, C. Sors, J. Birembaum, M. Le Lirzin, and G. Canetti. 1969. '[Comparison between regimes of rifampicin-isoniazid administered daily and administered twice a week (initial results of a comparative study conducted in 4 medical services of the Parisian region)]', *Rev Tuberc Pneumol (Paris)*, 33: 751-68.

Decroix, G., C. Sors, and J. C. Pujet. 1974. '[Complications of intermittent rifampicin treatment. Role of immuno-allergic factors. Study of 8 cases]', *Nouv Presse Med*, 3: 1555-7.

Diacon, A. H., R. F. Patientia, A. Venter, P. D. van Helden, P. J. Smith, H. McIlleron, J. S. Maritz, and P. R. Donald. 2007. 'Early bactericidal activity of high-dose rifampin in patients with pulmonary tuberculosis evidenced by positive sputum smears', *Antimicrob Agents Chemother*, 51: 2994-6.

Diallo, T., M. Adjobimey, R. Ruslami, A. Trajman, O. Sow, J. Obeng Baah, G. B. Marks, R. Long, K. Elwood, D. Zielinski, M. Gninafon, D. A. Wulandari, L. Apriani, C. Valiquette, F. Fregonese, K. Hornby, P. Z. Li, P. C. Hill, K. Schwartzman, A. Benedetti, and D. Menzies. 2018. 'Safety and Side Effects of Rifampin versus Isoniazid in Children', *N Engl J Med*, 379: 454-63.

Dian, S., V. Yunivita, A. R. Ganiem, T. Pramaesya, L. Chaidir, K. Wahyudi, T. H. Achmad, A. Colbers, L. Te Brake, R. van Crevel, R. Ruslami, and R. Aarnoutse. 2018. 'Double-Blind, Randomized, Placebo-Controlled Phase II Dose-Finding Study To Evaluate High-Dose Rifampin for Tuberculous Meningitis', *Antimicrob Agents Chemother*, 62.

Doan, T. N., G. J. Fox, M. T. Meehan, N. Scott, R. Ragonnet, K. Viney, J. M. Trauer, and E. S. McBryde. 2019. 'Cost-effectiveness of 3 months of weekly rifapentine and isoniazid compared with other standard treatment regimens for latent tuberculosis infection: a decision analysis study', *J Antimicrob Chemother*, 74: 218-27.

Dobler, C. C., S. Bosnic-Anticevich, and C. L. Armour. 2018. 'Physicians' perspectives on communication and decision making in clinical encounters for treatment of latent tuberculosis infection', *ERJ Open Res*, 4.

Dorman, S. E., R. M. Savic, S. Goldberg, J. E. Stout, N. Schluger, G. Muzanyi, J. L. Johnson, P. Nahid, E. J. Hecker, C. M. Heilig, L. Bozeman, P. J. Feng, R. N. Moro, W. MacKenzie, K. E. Dooley, E. L. Nuermberger, A. Vernon, M. Weiner, and Consortium Tuberculosis Trials. 2015. 'Daily rifapentine for treatment of pulmonary tuberculosis. A randomized, dose-ranging trial', *Am J Respir Crit Care Med*, 191: 333-43.

Elzi, L., M. Schlegel, R. Weber, B. Hirschel, M. Cavassini, P. Schmid, E. Bernasconi, M. Rickenbach, H. Furrer, and H. I. V. Cohort Study Swiss. 2007. 'Reducing tuberculosis incidence by tuberculin skin testing, preventive treatment, and antiretroviral therapy in an area of low tuberculosis transmission', *Clin Infect Dis*, 44: 94-102.

Ena, J., and V. Valls. 2005. 'Short-course therapy with rifampin plus isoniazid, compared with standard therapy with isoniazid, for latent tuberculosis infection: a meta-analysis', *Clin Infect Dis*, 40: 670-6.

Fox GJ, Barry SE, Britton WJ, and Marks GB. 2013. 'Contact investigation for tuberculosis: a systematic review and meta-analysis', *Eur Respir J*, 41: 140-56.

Gao, XF., L. Wang, GJ. Liu, J. Wen, X. Sun, Y. Xie, and YP. Li. 2006. 'Rifampicin plus pyrazinamide versus isoniazid for treating latent tuberculosis infection: a meta-analysis', *Int J Tuberc. Lung Dis*, 10: 1080-90.

Geijo, MP., CR. Herranz, P. Vano, AJ. Garcia, M. Carcia, and JF. Dimas. 2007. 'Pauta corta de isoniazida y rifampicina comparada con isoniazida para la infeccion latente de tuberculosis. Ensayo clinica aleatorizado', *Enfermedades Infeccionas Microbiologia Clinica*, 25: 300.

Gordin, FM., JP. Matts, C. Miller, LS. Brown, and et al. 1997. 'A controlled trial of isoniazid in persons with anergy and human immunodeficiency virus infection who are at high risk for tuberculosis', *New Engl J Med*, 337: 315-20.

Hawken, MP., HK. Meme, JM. Chakaya, LC. Elliott, JS. Morris, WA. Githui, ES. Juma, JA. Odhiambo, LN. Thiong’o, JN. Kimari, EN. Ngugi, JJ. Bwayo, CF. Gilks, FA. Plummer, JD. Porter, PP. Nunn, and KP. McAdam. 1997. 'Isoniazid preventive therapy for tuberculosis in HIV-infected adults: results of a randomized controlled trial', *AIDS*, 11: 875-82.

Heemskerk, A. D., N. D. Bang, N. T. Mai, T. T. Chau, N. H. Phu, P. P. Loc, N. V. Chau, T. T. Hien, N. H. Dung, N. T. Lan, N. H. Lan, N. N. Lan, T. Phong le, N. N. Vien, N. Q. Hien, N. T. Yen, D. T. Ha, J. N. Day, M. Caws, L. Merson, T. T. Thinh, M. Wolbers, G. E. Thwaites, and J. J. Farrar. 2016. 'Intensified Antituberculosis Therapy in Adults with Tuberculous Meningitis', *N Engl J Med*, 374: 124-34.

Hong Kong Chest Service Tuberculosis Research Centre, Madras.British Medical Research Council. 1992. 'A double-blind placebo-controlled clinical trial of three antituberculosis chemoprophylaxis regimens in patients with silicosis in Hong Kong', *Am Rev Respir Dis*, 145: 36-41.

International Union Against Tuberculosis Committee on Prophylaxis. 1982. 'Efficacy of various durations of isoniazid preventive therapy for tuberculosis: five years of follow-up in the IUAT trial', *Bull Wld Hlth Org*, 60: 555-64.

Jindani A, Harrison TS, Nunn AJ, Philips PPJ, Churchyard GJ, Charalambous S, Hatherill M, Geldenhuys H, Mclleron HM, Svada SP, Mungofa S, Shah NA, Zizhou S, Magweta L, Shepherd J, Nyirenda s, van Dijk JH, Clouting HE, Coleman D, Bateson ALE, McHugh TD, Butcher PD, and Mitchison DA. 2014. 'High-Dose Rifapentine with Moxifloxacin for Pulmonary Tuberculosis', *N Engl J Med*, 371: 1599-608.

Jindani, A., G. Borgulya, I. W. de Patino, T. Gonzales, R. A. de Fernandes, B. Shrestha, D. Atwine, M. Bonnet, M. Burgos, F. Dubash, N. Patel, A. M. Checkley, T. S. Harrison, D. Mitchison, and St George's University of London International Consortium for Trials of Chemotherapeutic Agents in Tuberculosis. 2016. 'A randomised Phase II trial to evaluate the toxicity of high-dose rifampicin to treat pulmonary tuberculosis', *Int J Tuberc Lung Dis*, 20: 832-8.

Kopanoff, D. E., D. E. Snider, Jr., and G. J. Caras. 1978. 'Isoniazid-related hepatitis: a U.S. Public Health Service cooperative surveillance study', *Am Rev Respir Dis*, 117: 991-1001.

Long, MW., Jr.DE. Snider, and LS. Farer. 1979. 'U.S. Public health service cooperative trial of three rifampin-isoniazid regimens in treatment of pulmonary tuberculosis', *Am. Rev. Resp. Dis*, 119: 879-94.

Martinez Alfaro, E. M., F. Cuadra, J. Solera, M. A. Macia, P. Geijo, P. A. Sanchez Martinez, M. Rodriguez Zapata, J. Largo, M. A. Sepulveda, C. Rosa, L. Sanchez, A. Espinosa, F. Mateos, and J. J. Blanch. 2000. '[Evaluation of 2 tuberculosis chemoprophylaxis regimens in patients infected with human immunodeficiency virus. The GECMEI Group]', *Med Clin (Barc)*, 115: 161-5.

Martinez Alfaro, E., J. Solera, E. Serna, D. Cuenca, M. L. Castillejos, A. Espinosa, and L. Saez. 1998. '[Compliance, tolerance and effectiveness of a short chemoprophylaxis regimen for the treatment of tuberculosis]', *Med Clin (Barc)*, 111: 401-4.

Menzies D, Long R, Trajman A, Dion MJ, Yang J, Al-Jahdali H, Memish ZA, Khan K, Gardam M, Hoeppner VH, Benedetti A, and Schwartzman K. 2008. 'Adverse events with 4 months rifampin or 9 months isoniazid as therapy for latent TB infection: results of a randomized trial.', *Annals of Internal Medicine*, 149: 689-97.

Menzies, D., M. Adjobimey, R. Ruslami, A. Trajman, O. Sow, H. Kim, J. Obeng Baah, G. B. Marks, R. Long, V. Hoeppner, K. Elwood, H. Al-Jahdali, M. Gninafon, L. Apriani, R. C. Koesoemadinata, A. Kritski, V. Rolla, B. Bah, A. Camara, I. Boakye, V. J. Cook, H. Goldberg, C. Valiquette, K. Hornby, M. J. Dion, P. Z. Li, P. C. Hill, K. Schwartzman, and A. Benedetti. 2018. 'Four Months of Rifampin or Nine Months of Isoniazid for Latent Tuberculosis in Adults', *N Engl J Med*, 379: 440-53.

Morrison J, Pai M, and Hopewll PC. 2008. 'Tuberculosis and latent tuberculosis infection in close contacts of people with pulmonary tuberculosis in low-income andmiddle -income countries:a systematic review and meta-analysis', *Lancel Infect Dis*, 8: 368.

Nitti, V. 1973. 'Controlled Clinical Evaluation of Three Intermittent Regimens Employing Low-dosage Schedules of Rifampicin in Original Treatment of Pulmonary Tuberculosis', *Scand. J. Resp. Dis*, supplement: 180-85.

Njie, G. J., S. B. Morris, R. Y. Woodruff, R. N. Moro, A. A. Vernon, and A. S. Borisov. 2018. 'Isoniazid-Rifapentine for Latent Tuberculosis Infection: A Systematic Review and Meta-analysis', *Am J Prev Med*, 55: 244-52.

Onofre Moran-Mendoza, A. 2004. 'The value of the tuberculin skin test size in predicting the development of tuberculosis in contacts of active cases', Department of Health Care and Epidemiology, University of British Columbia.

Pease, C., B. Hutton, F. Yazdi, D. Wolfe, C. Hamel, P. Quach, B. Skidmore, D. Moher, and G. G. Alvarez. 2017. 'Efficacy and completion rates of rifapentine and isoniazid (3HP) compared to other treatment regimens for latent tuberculosis infection: a systematic review with network meta-analyses', *BMC Infect Dis*, 17: 265.

Pocock, S. J., T. C. Clayton, and G. W. Stone. 2015. 'Challenging Issues in Clinical Trial Design: Part 4 of a 4-Part Series on Statistics for Clinical Trials', *J Am Coll Cardiol*, 66: 2886-98.

Rivero, A., L. Lopez-Cortes, R. Castillo, F. Lozano, M. A. Garcia, F. Diez, J. C. Escribano, J. Canueto, J. Pasquau, J. J. Hernandez, R. Polo, F. J. Martinez-Marcos, J. M. Kindelan, R. Rey, and Infecciosas Grupo Andaluz para el estudio de las Enfermedades. 2003. '[Randomized trial of three regimens to prevent tuberculosis in HIV-infected patients with anergy]', *Enferm Infecc Microbiol Clin*, 21: 287-92.

Ronald LA, FitzGerald JM, Bartlett-Esquilant G, Schwartzman K, Benedetti A, Boivin J, Menzies D. In press. 'Treatment with Isoniazid or Rifampin for Latent Tuberculosis Infection: Population-Based Study of Hepatotoxicity, Completion, and Costs.', *EJR*.

Rosenthal, I. M., R. Tasneen, C. A. Peloquin, M. Zhang, D. Almeida, K. E. Mdluli, P. C. Karakousis, J. H. Grosset, and E. L. Nuermberger. 2012. 'Dose-ranging comparison of rifampin and rifapentine in two pathologically distinct murine models of tuberculosis', *Antimicrob Agents Chemother*, 56: 4331-40.

Ruslami, R., A. R. Ganiem, S. Dian, L. Apriani, T. H. Achmad, A. J. van der Ven, G. Borm, R. E. Aarnoutse, and R. van Crevel. 2013. 'Intensified regimen containing rifampicin and moxifloxacin for tuberculous meningitis: an open-label, randomised controlled phase 2 trail', *Lancet Infect Dis*, 13: 27-35.

Ruslami, R., HJ. Nijland, B. Alisjahbana, I. Parwait, R. van Crevel, and RE. Aarnouste. 2007. 'Pharmacokinetics and tolerability of a higher rifampicin dose versus the standard dose in pulmonary tuberculosis patients', *Antimicrob Agents Chemothe*, 51: 2551.

Salpeter, S. R. 1993. 'Fatal isoniazid-induced hepatitis. Its risk during chemoprophylaxis', *West J Med*, 159: 560-4.

Saukkonen, J.J., D.L. Cohn, R.M. Jasmer, S. Schenker, J.A. Jereb, C.M. Nolan, C.A. Peloquin, F.M. Gordin, D. Nunes, D.B. Strader, J. Bernardo, R. Venkataramanan, and T.R. Sterling. 2006. 'An official ATS statement: hepatotoxicity of antituberculosis therapy', *Am J Respir Crit Care Med*, 174: 935-52.

Seijger, C., W. Hoefsloot, I. Bergsma-de Guchteneire, L. Te Brake, J. van Ingen, S. Kuipers, R. van Crevel, R. Aarnoutse, M. Boeree, and C. Magis-Escurra. 2019. 'High-dose rifampicin in tuberculosis: Experiences from a Dutch tuberculosis centre', *PLoS One*, 14: e0213718.

Singapore Tuberculosis Service British Medical Research Council. 1975. 'Controlled trial of intermittent regimens of rifampicin plus isoniazid for pulmonary tuberculosis in Singapore', *The Lancet*, 306: 1105-09.

Smieja MJ, Marchetti CA, Cook DJ, and Smaill FM. 2000. 'Isoniazid for preventing tuberculosis in non-HIV infected persons', *Cochrane Database Syst. Rev*: CD001363.

Snider, D. E., Jr., and G. J. Caras. 1992. 'Isoniazid-associated hepatitis deaths: a review of available information', *Am Rev Respir Dis*, 145: 494-7.

Spyridis NP, Spyridis PG, Gelesme A, Sypsa V, Valianatou M, Metsou F, Gourgiotis D, and Tsolia MN. 2007. 'The Effectiveness of a 9-Month Regimen of Isoniazid Alone versus 3- and 4- Month Regimens of Isoniazid plus Rifampin for Treatment of Latent Tuberculosis Infection in Children: REsults of an 11-Year Randomized Study', *Clinical Infectious Diseases*, 45: 715.

Stead, W. W., J. P. Lofgren, E. Warren, and C. Thomas. 1985. 'Tuberculosis as an endemic and nosocomial infection among the elderly in nursing homes', *N Engl J Med*, 312: 1483-7.

Sterling TR, Moro RN, Borisov AS, Phillips E, Shepherd G, Adkinson NF, Weis S, Ho C, and Villarino ME. 2015. 'Flu-like and Other Systemic Drug Reactions Among Persons Receiving Weekly Rifapentine plus Isoniazid or Daily Isoniazid for Treatment of Latent Tuberculosis Infection in the PREVENt Tuberculosis Study', *Clin Infec Dis*, 61: 527-35.

Sterling TR, Villarino ME, Borisov AS, Shang N, Gordin F, Bliven-Sizemore E, Hackman J, Hamilton CD, Menzies D, Kerrigan A, Weis SE, Weiner M, Wing D, Conde MB, Bozeman L, Horsburg Jr CR, Chaisson RE, and and the TB Trials Consortium. 2011. 'Three months of rifapentine and isoniazid for latent tuberculosis infection', *New Engl J Med*, 365.

Sterling TR, Njie G, Zenner D, Cohn DL, et al. In press. 'Guidelines for the Treatment of Latent Tuberculosis Infection (NTCA/CDC)', *MMWR*.

Svensson, R. J., R. E. Aarnoutse, A. H. Diacon, R. Dawson, S. H. Gillespie, M. J. Boeree, and U. S. H. Simonsson. 2018. 'A Population Pharmacokinetic Model Incorporating Saturable Pharmacokinetics and Autoinduction for High Rifampicin Doses', *Clin Pharmacol Ther*, 103: 674-83.

Swindells, S., R. Ramchandani, A. Gupta, C. A. Benson, J. Leon-Cruz, N. Mwelase, M. A. Jean Juste, J. R. Lama, J. Valencia, A. Omoz-Oarhe, K. Supparatpinyo, G. Masheto, L. Mohapi, R. O. da Silva Escada, S. Mawlana, P. Banda, P. Severe, J. Hakim, C. Kanyama, D. Langat, L. Moran, J. Andersen, C. V. Fletcher, E. Nuermberger, R. E. Chaisson, and Brief Tb A. Study Team. 2019. 'One Month of Rifapentine plus Isoniazid to Prevent HIV-Related Tuberculosis', *N Engl J Med*, 380: 1001-11.

Uplekar, M., D. Weil, K. Lonnroth, E. Jaramillo, C. Lienhardt, HM. Dias, D. Falzon, K. Floyd, G. Gargioni, H. Getahun, C. Gilpin, P. Glaziou, M. Grzemska, F. Mirzayev, H. Nakatani, and M. Raviglione. 2015. 'WHO's new End TB Strategy', *Lancet*, 385: 1799-801.

van Beek, S. W., R. Ter Heine, R. J. Keizer, C. Magis-Escurra, R. E. Aarnoutse, and E. M. Svensson. 2019. 'Personalized Tuberculosis Treatment Through Model-Informed Dosing of Rifampicin', *Clin Pharmacokinet*.

Velasquez, G. E., M. B. Brooks, J. M. Coit, H. Pertinez, D. Vargas Vasquez, E. Sanchez Garavito, R. I. Calderon, J. Jimenez, K. Tintaya, C. A. Peloquin, E. Osso, D. B. Tierney, K. J. Seung, L. Lecca, G. R. Davies, and C. D. Mitnick. 2018. 'Efficacy and Safety of High-Dose Rifampin in Pulmonary Tuberculosis. A Randomized Controlled Trial', *Am J Respir Crit Care Med*, 198: 657-66.

Whalen, C. C., J. L. Johnson, A. Okwera, D. L. Hom, R. Huebner, P. Mugyenyi, R. D. Mugerwa, and J. J. Ellner. 1997. 'A trial of three regimens to prevent tuberculosis in Ugandan adults infected with the human immunodeficiency virus. Uganda-Case Western Reserve University Research Collaboration', *N Engl J Med*, 337: 801-8.

World Health Organization. 2014. "Guidance for national tuberculosis programmes on the management of tuberculosis in children –

2nd ed." In.

———. 2015. "The End TB Strategy." In.

———. 2018a. "Latent TB Infection : Updated and consolidated guidelines for programmatic management." In. <https://www.who.int/tb/publications/2018/latent-tuberculosis-infection/en/>.

———. 2018b. "World leaders commit to bold targets and urgent action to end TB." In.

Yuan, L., E. Richardson, and PRW. Kendall. 1995. 'Evaluation of a tuberculsosis screening program for high-risk students in Toronto schools', *Can Med Assoc J*, 153: 925-32.

Yunivita, V., S. Dian, A. R. Ganiem, E. Hayati, T. Hanggono Achmad, A. Purnama Dewi, M. Teulen, P. Meijerhof-Jager, R. van Crevel, R. Aarnoutse, and R. Ruslami. 2016. 'Pharmacokinetics and safety/tolerability of higher oral and intravenous doses of rifampicin in adult tuberculous meningitis patients', *Int J Antimicrob Agents*, 48: 415-21.

Zenner, D., N. Beer, R. J. Harris, M. C. Lipman, H. R. Stagg, and M. J. van der Werf. 2017. 'Treatment of Latent Tuberculosis Infection: An Updated Network Meta-analysis', *Ann Intern Med*, 167: 248-55.

Zhang, T., M. Zhang, I. M. Rosenthal, J. H. Grosset, and E. L. Nuermberger. 2009. 'Short-course therapy with daily rifapentine in a murine model of latent tuberculosis infection', *Am J Respir Crit Care Med*, 180: 1151-7.
