# Supplementary material for: High dose rifampin for 2 months vs standard dose rifampin for 4 months, to treat TB infection: Protocol of a 3-arm randomized trial (2R2)
Source: PLoS One. 2023 Feb 2;18(2):e0278087. doi: 10.1371/journal.pone.0278087 (PMC9894386; doi:10.1371/journal.pone.0278087)
Supplement: S2 File — (DOCX) [file pone.0278087.s003.docx]

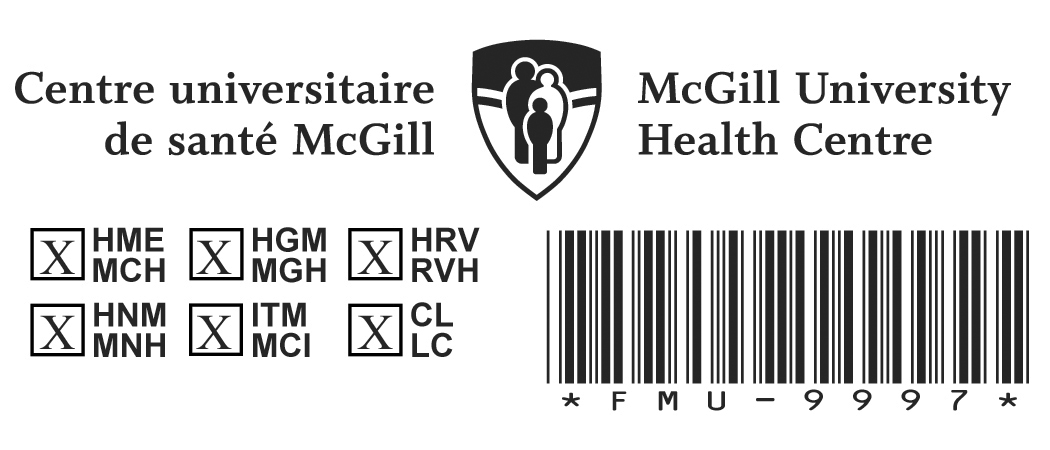

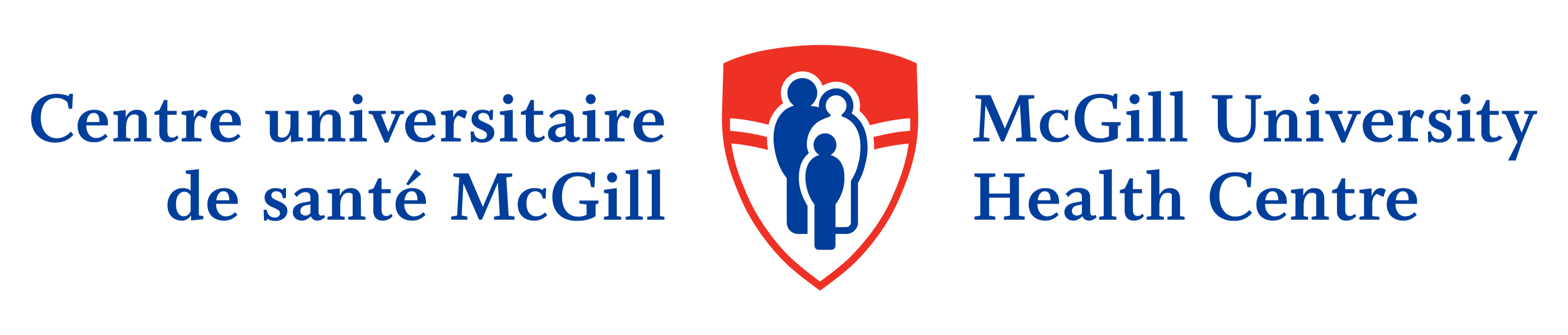


**RESEARCH INFORMATION AND CONSENT FORM**

**A 3-arm randomized clinical trial to assess completion and safety of higher dose Rifampin for 2 months vs Standard dose Rifampin for Latent TB infection (2R^2^)**

**Persons responsible:**

Principal Investigator: Dr. Dick Menzies - McGill University Health Center, Montreal Chest Institute

Co-investigator: Dr. David Zielinski - Montreal Children’s Hospital

**Funding Source:** Canadian Institutes of Health Research

**WHY ARE YOU BEING INVITED TO TAKE PART IN THIS STUDY?**

The Respirology department/service conducts research studies to try to improve treatment for people with respiratory infections. Today, we are inviting you to take part in a research study. Please read this information to help you decide if you want to participate in this research project. It is important that you understand this information. We encourage you to ask questions. Please take all the time you need to make your decision.

This study will include both adults and children. We encourage parents to include their child in the discussion and decision making to the extent that the child is able to understand.

In this research information and consent form, “you” means you or your child.

We are asking you to take part in this research study because your doctor thinks that you have been infected with the tuberculosis (TB) germ, and that you now have “latent” TB. This can happen when you have been in contact with someone who is sick with a disease called tuberculosis (or TB). Latent TB is an infection that will not cause you to feel sick or unwell right now. However, it can cause some people with the infection to become sick with TB disease later if they do not take any treatment.

**WHY IS THIS STUDY BEING DONE?**

We are doing a research study because we want to find new, shorter treatments to get rid of this TB infection, and to stop people who have been infected with the TB germ from becoming sick later.

Right now, the drug that we use to get rid of the infection is called Rifampin. This drug has to be taken every day for 4 months time. About 9 people out of 10 who take this drug are cured of their infection. But taking a drug every day for 4 months is a long time. This is why we are looking for a new treatment that is shorter. We think that people could take more of the Rifampin drug (for example, two or three times more than the usual dose) for only 2 months instead of 4. We want to know if this would cure the infection just as well without causing any additional side effects and if people will be more likely to complete the treatment if it is shorter.

We are doing this study to find out if two or three times the normal dose of the drug Rifampin is safe, if it works as well for curing the TB infection and if it helps people to complete the full treatment.

**HOW MANY PEOPLE WILL TAKE PART IN THIS STUDY?**

About 1359 people with latent TB are expected to participate in this study around the world. About 100 will be recruited at the MUHC (adults and children combined).

**WHAT WILL HAPPEN ON THIS RESEARCH STUDY?**

People who agree to participate in this study will:

1. Undergo a pregnancy test if you are a female and able to get pregnant. (Pregnant women cannot participate in this study.
2. Be assigned to one of three possible treatments groups.

iii) Take your study treatment as described and attend study visits during treatment.

iv) Have 1 extra blood test. For adults at the MUHC it is routine practice that one blood test is done before latent TB treatment starts and a second test is done after 4 weeks. As part of the study: for all participants one extra blood test will be done at the time of the 4 weeks visit. The participants assigned to either of the two high dose 2 month treatment groups will also have a blood test done at the time of the first follow-up visit - 2 weeks after starting treatment. For children at the MUHC it is not routine practice to do any blood tests for latent TB treatment. In this study, three or four blood tests will be done. For all children participating in the study: one blood test will be done before treatment starts and two blood test at the time of the 4 weeks follow-up visit. Children who receive one of the two higher dose treatments will also have a blood test done at the time of the first follow-up visit 2 weeks after starting treatment.

v) Receive phone calls every 3 months for about 2 years once the treatment is completed.

Participants in this study will be put in one of three treatment groups. The first group will take the “normal” treatment, which is Rifampin at a normal dose (10 mg/kg/day) for 4 months. The second group will take Rifampin at two times the normal dose (20 mg/kg/day) for 2 months. The third group will take Rifampin at three times the normal dose (30 mg/kg/day) for 2 months.

The choice of which group you will be in during the study will be made by chance (like flipping a coin). You have an equal chance of being in any of the 3 groups. You or your doctor cannot choose which group you are in. By leaving the decision to chance we will make sure that the people in the 3 groups are all similar, except for the specific drug that they are taking. If you are assigned to take the drug for 2 months time, you or your doctor will not know if you are being given two or three times the normal dose of Rifampin. We do this to make sure that when we analyze the 2 groups we do so as fairly and objectively as possible.

During the study, you will come back to the hospital for 3 visits. The first visit will be 2 or 4 weeks after you started taking your medications. The second visit is 2 or 4 weeks after the first. The last visit will be close to the end of your treatment. For adults, these visits are timed to coincide with the routine follow up visits, for children, we routinely do one or two (or any) follow up visits so there may be up to 3 extra visits for study purposes.

At every visit please BRING YOUR MEDICATION BOTTLE to show the nurse. The nurse will ask you about the treatment that you are taking and if you have had any problems taking it. Your doctor will also check that you are well.

If during your participation in this study, the hospital provides care through virtual visits (telephone) instead of in-person visits for people receiving treatment for LTBI, your doctor and nurses will contact you by telephone to ask you about your health, instead of asking you to come to the hospital. However if blood tests are needed, then you will have to come to have the blood samples taken.

You will also be asked to have blood tests. A first blood test will be done before you start taking your medications. For adults, these are the normal blood tests that you would have anyway, even if you were not participating in this study. A second blood test will be done at the time of the follow-up visit, four weeks after you start taking the medication. On the day of this visit, you will need to take your medication at home in the morning, without eating any food at home before or after taking the medications. After you have taken your medication, you will come to the hospital for your appointment in time to have a blood test about 2 hours after you took your medication. The nurse will do the blood test. Then you will be able to have your breakfast. In order to do so, you will be given a credit voucher to buy your breakfast in the Hospital cafeteria. The nurse and doctor will then see you as usual. Two hours after the first blood test you will have a second blood test done. You can then leave the hospital. We are doing these blood tests for two reasons. One is to check that the drug that you are taking is not causing you any harm. The second reason is to check how much Rifampin there is in your blood. If you are taking rifampin at double or triple dose, there will also be a third blood test, done at the time of the follow-up visit two weeks after you start taking the medication.

Your doctor may want to see you more than just 3 times, or you may need to have more blood tests done. This will depend on what your doctor thinks is best for you. If your doctor wants to see you more often, or do more blood tests, they will discuss that with you.

After you have finished taking your treatment, we will call you on the phone every 3 months for about 2 years. We will also send your name to an office that keeps records of people who develop TB disease (called the Public Health Department). This is not usually done for patients with latent TB but is necessary for us to be sure that a shorter treatment time is as effective at preventing active TB disease. They will tell us if you have developed TB disease since the study ended or if you were treated for TB at another hospital.

FOR HOW LONG WILL YOU PARTICIPATE IN THIS STUDY?

We expect that you will be part of this study for the duration of latent TB treatment (that is 2 or 4 months) and for 2 years following the end of your treatment. After you have finished taking your medication, we will call you on the phone every 3 months for 2 years.

WHAT ARE THE RISKS?

Rifampin is a safe drug that has been used by millions of people to treat TB disease for about 45 years. Sometimes when people take Rifampin they have minor side effects such as a headache, feeling tired or weak, having an upset stomach, heartburn, diarrhea, itchiness, rash or not feeling hungry. These usually get better by themselves, and it is not necessary to stop the treatment. Some people can have a more serious reaction that involves your liver, but this is very rare (less than 1 in 100 people have this kind of reaction), and your body usually returns to normal once you stop taking the medication. If you develop a serious reaction the medication will be stopped and your doctor will discuss with you about alternative treatment or alternative follow-up.

Rifampin can also cause your tears, sweat, spit, bowel movements and urine to turn orange. If this happens, things will return to their normal color once you stop taking your treatment. If you wear contact lenses, they might stay stained with this orange color.

Rifampin can affect other medications, including birth control medications, and stop them from working properly. You should tell the nurse, your doctor, or the study coordinator about ALL of the medications you are taking (prescribed or bought at the pharmacy). This includes any drugs you are taking now or start during the research study.

When Rifampin was given at two times or three times the usual dose it has been well tolerated in other research studies. But we do not know if these doses will be tolerated as well when it is given for a longer time (2 months) as in this study. For participants assigned to the higher doses of Rifampin, there may be more side effects than if you were taking the usual dose. Also, we do not yet know if taking Rifampin at a higher dose for a shorter amount of time will cure the infection as well as taking the standard dose for the usual amount of time. If you need to interrupt the treatment because you cannot tolerate it, your doctor will discuss with you possible alternative treatments or follow-up.

Remember that Rifampin at any dose (like any drug) can cause side effects – so if you experience any symptoms while taking this treatment, or have any concerns, you should contact:

-For adults: the TB clinic nurses at (514) 934-1934 ext. 32588; or you can come to the Montreal Chest Institute clinic as a ‘walk-in’ anytime from Monday to Friday between 8am and 4pm; at other times you can come to the MUHC (Glen) emergency room;

-For children: the Montreal Children’s Hospital TB clinic nurses (514-412-4400 ext. 22843) or come to the Montreal Children’s Hospital emergency room, which is open 24 hours every day.

BLOOD TEST:

For adults: Usually, for adults who take latent TB treatment there is one test done at the beginning of the study and one at about 4 weeks after treatment started. In addition to these two tests, done routinely, for all participants there will be another test done at the visit after 4 weeks of treatment. For participants in two of the assigned treatment groups there will also be a test done at the visit after 2 weeks of treatment.

For children: As part of this study you will have to do 3 or 4 (depending on the treatment group) extra blood tests: one at the beginning of the study; one at the visit of 2 weeks (if in the double or triple rifampin group) and two in the same day of the visit after about 4 weeks from the treatment started.

Blood tests may cause you some discomfort. You could have a brief pain where the needle is put in your skin, bruising or dizziness. It is very rare for an infection to develop where the needle is put in your skin. A cream (EMLA) can be used before the test, to reduce the discomfort in children. The total amount of blood taken for each test is 5ml (a teaspoon).

FOR FEMALE PARTICIPANTS

You should not take part in this study if you are pregnant or plan to become pregnant before completing your treatment.

As pregnant women cannot participate in this study, female participants who can get pregnant will have a pregnancy test before being included in the study. If you are pregnant, you will not be able to participate. Pregnancy test results will be returned confidentially to all participants who are at least 14 years of age. For younger participants, positive pregnancy test results will be returned to both the participant and her parent. Female participants must also take all necessary measures to avoid becoming pregnant while on the TB treatment.

Rifampin can make many forms of birth control, including the birth control pill, hormonal injections and implants work less well. If you are using any of these methods for birth control, you should speak to your doctor about other methods of birth control to use. Methods that are not effected by rifampin include not having sex, condoms, diaphragms, sponges, cervical caps or intra-uterine devices. Although Rifampin is not known to cause any birth defects, if you do become pregnant while you are taking your medication, you will have to stop taking your treatment. Your doctor will decide with you about re-starting your treatment at a later time.

SHOULD YOU SUFFER ANY HARM:

Should you suffer harm of any kind following administration of the study drug, or following any other procedure related to the research study, you will receive the appropriate care and services required by your state of health.

By agreeing to participate in this research project, you are not waiving any of your legal rights nor discharging the study doctor, the sponsor or the institution, of their civil and professional responsibilities.

ARE THERE BENEFITS TO TAKING PART IN THE STUDY?

By taking part in this research study you can help us learn more about how to treat TB infection (or “latent TB”). There is no other specific reason or advantage to taking part in this study.

WHAT OTHER OPTIONS ARE THERE?

If you choose not to take part in this study, you will be treated with the standard treatment for latent TB infection – which is Rifampin at a standard dose every day for 4 months. You do not have to participate in this study to receive treatment for latent TB.

**WHAT ARE THE COSTS OF TAKING PART IN THIS STUDY FOR ADULTS?**

You will not have to pay for the treatment or any future visits or tests that are part of this study. There is no compensation for participation in this study. However, during the visit one month after treatment has started, you will be given credit for breakfast in the Hospital cafeteria

**WHAT ARE THE COSTS OF TAKING PART IN THIS STUDY FOR CHILDREN?** If you are a child or adolescent participating in the study, you will be given a credit for breakfast in the Hospital cafeteria for the visit one month after treatment has started. Since there are up to three extra visits in the case of children, you will also receive a 12$ voucher for each of the three visits, to partially contribute to the hospital parking costs. If visit duration is under 1 hour, this will cover the whole parking fee. Had the visit to last longer, there may be additional costs that are not reimbursed by the study.

HOW IS PRIVACY ENSURED?

During your participation in this study, the study doctor and their team will collect and record information about you in a study file. They will only collect information required to meet the scientific goals of the study.

The study file may include information from your medical chart, concerning your past and present state of health, your lifestyle, as well as your sex, the results of the tests, exams, and procedures that you will undergo during this research project.

The two coded blood samples done at the 4 weeks visit, for pharmacokinetic study, will be sent to the Bandung University (in Bandung, Indonesia) laboratory of professor Rovina Ruslami, a study co-investigator who will perform the analysis for all study sites. Blood will be stored until study is completed and data analyzed. This blood will be used for the exclusive objectives of this study and then destroyed.

All the information collected during the research project will remain strictly confidential to the extent provided by law. You will only be identified by a code number. The key to the code linking your name to your study file will be kept by the study doctor. However, at the end of the study (i.e. after 2 years from when treatment stops) your name and birth date will be sent to provincial health authorities so that we can be informed if any of our study participants had developed active TB following this treatment. These provincial health authorities will not keep this list of names. After checking to see if anyone on the list developed active TB, the list will be destroyed.

To ensure your safety, a copy of this information and consent form will be placed in your medical chart. As a result, any person or company to whom you give access to your medical chart will have access to this information.

The study data will be stored for 25 years.

The data may be published or shared during scientific meetings; however, it will not be possible to identify you.

For monitoring, control, safety, security, and marketing of a new study drug, or a new dosage of a standard drug (as in this study) your study file as well as your medical charts may be examined by a person mandated by Canadian or international regulatory authorities, such as Health Canada, as well as by representatives of the study sponsor, the institution, or the Research Ethics Board. All these individuals and organizations adhere to policies on confidentiality.

You have the right to consult your study file in order to verify the information gathered, and to have it corrected if necessary.

However, in order to protect the scientific integrity of the research project, accessing certain information before the project is ended may require that you be withdrawn from the study.

**IS YOUR PARTICIPATION VOLUNTARY?**

Your participation in this research project is voluntary. Therefore, you may refuse to participate. You may also withdraw from the project at any time, without giving any reason, by informing the study doctor or a member of the research team.

Your decision not to participate in the study, or to withdraw from it, will have no impact on the quality of care and services to which you are otherwise entitled, or on your relationship with your doctor or clinical team.

The study doctor, the Research Ethics Board, the funding agency, or the Sponsor may put an end to your participation without your consent. This may happen if new findings or information indicate that participation is no longer in your interest, if you do not follow study instructions, or if there are administrative reasons to terminate the project.

If you withdraw or are withdrawn from the study, the information and biological material already collected for the study will be stored, analyzed and used to ensure the integrity of the study.

Any new findings that could influence your decision to stay in the research project will be shared with you as soon as possible.

**WHOM DO I CALL IF I HAVE QUESTIONS OR PROBLEMS?**

If you have any questions, either now or any time in the future, about this study, please feel free to discuss them with the people who are in charge of the study:

Dr. Dick Menzies (principal investigator) Montreal Chest Institute: 514-934-1934 ext. 32128

Valerya Yatsenko (clinical research coordinator), Montreal Chest Institute: 514-934-1934 ext. 44588; study cell phone 514-213-6520 (this number can be called at any time, 24 hours a day, seven days a week).

Amélie Fosso, Denis Francis and Octavian Boitor (nurse) at Montreal Chest Institute, at (514) 934-1934 ext: 32588 or 37217

Dr. David Zielinski (co-investigator) Montreal Children’s Hospital: 514-412-4400 ext. 24444

For any question concerning your rights as a research participant taking part in this study, or if you have comments, or wish to file a complaint, you may communicate with:

The Patient Ombudsman of the MUHC at the following phone number: **514 934 1934** ext 22223.

**WHERE CAN I GET MORE INFORMATION?**

Feel free to ask any questions that you might have about the study and what you have just heard. You can ask now, or at any time once the study has started.

A description of this clinical trial will also be available on http://www.ClinicalTrials.gov. This Website will not include information that can identify you. You can search this Website at any moment.

**RESEARCH ETHICS COMMITTEE**

The McGill University Health Centre Research Ethics Board reviewed this research and is responsible for monitoring the study

CONSENT AND ASSENT FORM

Title of this research project:  **A 3-arm randomized clinical trial to assess completion and safety of higher dose Rifampin for 2 months vs Standard dose Rifampin for Latent TB infection (2R^2^)**

I have been explained what will happen on this study. I read the information and consent form and was given a copy to keep. I was able to ask my questions and they were answered to my satisfaction. After thinking about it, I agree to, or I agree that my child will, participate in this research project.

I authorize the research team to consult my medical records or the medical records of my child to collect the information relevant to this project.

I authorize the research team to communicate my name and date of birth or my child’s name and date of birth to the public health authority solely for to the purpose of checking if I or my child developed TB in the 2 years following participation in this study.

In no way does consenting to participate in this research study waive your legal rights nor release the sponsor or the institution from their legal or professional responsibilities if you are harmed in any way.

Name of participant < 18 years old Assent of minor, capable of understanding Date

(Print) the nature of the research (signature) or

Verbal assent of minor obtained by:

Name of parent(s) or legal guardian Signature Date

(Print)

Name of participant (18 years +) Signature Date

(Print)

I have explained to the participant and/or his parent/legal guardian all the relevant aspects of this study. I answered any questions they asked. I explained that participation in a research project is free and voluntary and that they are free to stop participating at any time they choose.

Name of Person obtaining consent (signature) Date

(Print)

**Part to complete if the consent process has been done verbally, over the phone, to comply with COVID-19 related measures:**

Participant Name ____________________________________________________________

I have contacted the above named participant by telephone and explained to him/her and/or his parent/legal guardian all the relevant aspects of this study, by reading and explaining all the parts of this research information and consent form. I have answered any questions they asked. I have explained that participation in this research project is free and voluntary, and that they are free to stop participating at any time they choose.

I believe that the participant and/or his parent/legal guardian understands what is involved in the study and voluntarily agrees to participate.

Name of Person obtaining consent (signature) Date

(Print)

Name of impartial witness (signature) Date

(Print)

Note the impartial witness must be present during the whole consent.
