# Supplementary material for: High dose rifampin for 2 months vs standard dose rifampin for 4 months, to treat TB infection: Protocol of a 3-arm randomized trial (2R2)
Source: PLoS One. 2023 Feb 2;18(2):e0278087. doi: 10.1371/journal.pone.0278087 (PMC9894386; doi:10.1371/journal.pone.0278087)
Supplement: S4 File — (PDF) [file pone.0278087.s005.pdf]

Centre universitaire de santé McGill | McGill University Health Centre

Amendment/Modification to an approved study

Protocol title: **2R2: Higher dose Rifampin for 2 months vs Standard dose Rifampin for Latent TB: a 3-arm randomized trial**

Principal investigator: **Dick Menzies**

First submit date: **2021-09-22 11:51**

Submitted by: **Fregonese, Federica**

Project's REB approbation date: **2019-07-17**

Nagano identifier: **2R2**

Project number(s): **2019-5360**

Form: **F1H-84050**

Form status: **Approved**

Administration - REB

1. **MUHC REB Panel & Co-chair(s):**

   Pediatrics (PED)

   Co-chair: Vincent Lajoie

2. **REB Decision:**

   Approved - REB delegated review

3. **Comments on the decision:**

   The REB understands that this amendment had been the object of a CTA-N and does not require a NOL.

4. **Date of the REB final decision & signature**

   2021-09-30

   Signature

   Ms. Elizabeth Craven, Coordinator, MUHC REB
   MUHC REB Coordinator
   for MUHC Co-chair mentioned above
   2021-09-30 19:01

5. **ICF approved by the REB**

   2R2_ICF_Eng_Version 3_2021-09-20_REBapproved.docx

   2R2_ICF_FR_Version 3_2020-09-20_REBapproved .docx

6. **Document(s)approved by the REB**

2R2 Protocol_ShortHighDoseRIF LTBI treatment_Version 4_20Sept2021_REBapproved.docx

7. FWA 00000840 - FWA 00004545

8. **Local REB number**

IRB00010120

Affected sites

1. **The request concerns:**

all institutions

Indicate the status of the project

Project is in progress and recruitment is ongoing in at least one institution

General information

1. **Indicate the name of the Principal Investigator in our institution (MUHC)**

Menzies, Dick

From which department is the principal investigator?

Medicine

Division

Respiratory Medicine

Details about modification

1. **Indicate the nature of the request**

Amendment of the protocol

Yes

Specify the new version/date:

Version 4_20 September 2021

Please attach the amended protocol.

2R2 Protocol_ShortHighDoseRIF LTBI treatment_Version 4_20Sept2021_tracking.docx

2R2 Protocol_ShortHighDoseRIF LTBI treatment_Version 4_20Sept2021_CLEAN.docx

Amendment to the investigator brochure or product monograph

No

Amendment to an approved informed consent form

Yes

Specify the new version/date:

20 September 2021

Please attach the amended informed consent form.

2R2_ICF_Eng_Version 3_2021-09-20_tracking.docx

2R2_ICF_Eng_Version 3_2021-09-20_CLEAN.docx

2R2_ICF_FR_Version 3_2020-09-20_tracking .docx

2R2_ICF_FR_Version 3_2020-09-20_CLEAN .docx

Addition of a new informed consent form

No

Amended (or additional) documents used for the recruitment of participants

No

Amendment to participant questionnaires or other research documents to be given to participants

No

Addition of a new questionnaire or other research documents to be given to the participants

No

Change in the research team

Yes

Indicate the type of change

Other

Specify

Study site coordinator in Montreal is changing from Stephanie Senecal to Valerya Yatsenko, therefore contact name in ICF has been updated.

Change in the source of funding

No

Addition of a new site to the project/ converting a monocentric project into a multi-center project

No

other modifications, please specify

No

2. **Please summarize the amendment(s) made**

Please summarize the change(s) made

Montreal, 20 September 2021

To Research Ethic Board, RI-MUHC
Re: project 2R2 / 2019-5360

Dear Sir/Madam,

We are submitting an amendment for the clinical trial 2019-5360 “2R2: Higher dose Rifampin for 2 months vs Standard dose Rifampin for Latent TB: a 3-arm randomized trial”, a trial which started on September 2019, and which is currently ongoing in the 6 planned study sites.

The main change we propose in this amendment, has been suggested by an internal audit of QA department, which prompted a need for clarification of terminology regarding adverse events reporting and management in the study.

The wording explaining how all events in the study are reported and managed has been clarified at pages 30 and 31 of the protocol (edits in tracking).

We also took the opportunity to add a specification on the statistical significance that will be used in planned interim analyses (page 23).

Finally, as the research site coordinator in Montreal is changing this month, we are also submitting the Inform Consent Form in English and French with updated contact info for the new site coordinator (page 7 of ICF).

Please note that, for completeness, the appendix 2 of the protocol, which has been previously approved, has now been appended to the main protocol file, to have it all in one document.

Please let us know if any other information is needed,

Best regards,

Dick Menzies, Study PI, and, Federica Fregonese, trial coordinator.

Please justify the necessity for the amendment(s)

This amendment was done to clarify and give more details on the AE reporting process. Note: the process itself has not changed.

---

3. **Does this change require approval(acknowledgment) from Health Canada?**

No

Please explain why Health Canada authorization is not required

☐ This project is not a clinical trial governed by Health Canada regulations (Title 5)
☑ This is a clinical trial under Health Canada regulations but the nature of the modification does not require a new decision(NOL / authorization)

Please justify if necessary

We have sent Health Canada (HC) this amended protocol as CTA-Notification, as we consider that changes were mainly to increase clarity and do not change patients' enrollment or follow-up. In case HC asked for a full CTA-A instead: we will submit a CTA-A and will inform REB of this.

---

4. **Does this modifictaion affect the participants?**

No

Impact on feasibility

1. **Does the modification have an impact on other types of specific evaluations in our institution? (eg. use of resources, contract, budget, pharmacy, etc.)**

   No

Signature

Answer of: Fregonese, Federica

1. **I certify that the information provided on this form is correct.**

   Federica Fregonese
   2021-09-22 11:51
