# Supplementary material for: High dose rifampin for 2 months vs standard dose rifampin for 4 months, to treat TB infection: Protocol of a 3-arm randomized trial (2R2)
Source: PLoS One. 2023 Feb 2;18(2):e0278087. doi: 10.1371/journal.pone.0278087 (PMC9894386; doi:10.1371/journal.pone.0278087)
Supplement: S5 File — (PDF) [file pone.0278087.s006.pdf]

KEMENTERIAN RISET, TEKNOLOGI DAN PENDIDIKAN TINGGI
UNIVERSITAS PADJADJARAN
KOMISI ETIK PENELITIAN
RESEARCH ETHICS COMMITTEE
Jl. Prof. Eyckman No. 38 Bandung 40161
Telp. & Fax. 022-2038697 email: kepk.fk.unpad@gmail.com, website : kepk.fk.unpad.ac.id

No. Reg.: 0719091265

PERSETUJUAN ETIK
ETHICAL APPROVAL

Nomor: 1391 /UN6.KEP/EC/2019

Komisi Etik Penelitian Universitas Padjadjaran Bandung, dalam upaya melindungi hak asasi dan kesejahteraan subjek penelitian serta menjamin bahwa penelitian berjalan sesuai dengan pedoman *International Conference on Harmonisation – Good Clinical Practice (ICH-GCP)* dan aturan lainnya yang berlaku, telah mengkaji dengan teliti dan menyetujui proposal penelitian berjudul:

The Research Ethics Committee Universitas Padjadjaran Bandung, in an effort to protect the basic rights and welfare of the subject of the research and to assure that a research operates in accordance with International Conference on Harmonisation – Good Clinical Practice (ICH-GCP) guidlines and other applicable laws and regulations, has throughly reviewed and approved a reaserch proposal entitled:

"DOSIS TINGGI RIFAMPISIN 2 BULAN VERSUS DOSIS STANDAR RIFAMPISIN 4 BULAN UNTUK PENGOBATAN INFEKSI LATEN TB (ILTB): SEBUAH UJI KLINIK (2R2)"

Nama Peneliti Utama<br>*Principal Researcher*	:	Prof. Rovina Ruslami, dr., SpPD., Ph.D.
Pembimbing/Peneliti Lain<br>*Supervisor/Other Researcher*	:	Lika Apriani, dr., M.Sc.<br>Vycke Yunivita, dr., M.Kes.
Nama Institusi<br>*Institution*	:	Pusat Studi Infeksi Klinis<br>Fakultas Kedokteran Universitas Padjadjaran

proposal tersebut dapat disetujui pelaksanaannya.
hereby declare that the proposal is approved.

Ditetapkan di / *Issued in* : Bandung
Tanggal / *Date* : 20-11-2019

Ketua,
Chairman,

Dr. Meita Dhamayanti, dr., SpAK., M.Kes
NIP. 19630519 198712 2 001

Keterangan/*notes*:
Persetujuan etik ini berlaku selama satu tahun sejak tanggal ditetapkan.
This ethical clearance is effective for one year from the due date.
Pada akhir penelitian, laporan pelaksanaan penelitian harus diserahkan ke Komisi Etik Penelitian.
In the end of the research, progress and final summary report should be submitted to the Research Ethics Committee.
Jika ada perubahan atau penyimpangan protokol dan/atau perpanjangan penelitian, harus mengajukan kembali permohonan kajian etik penelitian.
If there be any protocol modification or deviation and/or extension of the study, the Principal Investigator is required to resubmit the protocol for approval.
Jika ada kejadian serius yang tidak diinginkan (KTD) harus segera dilaporkan ke Komisi Etik Penelitian.
If there are Serious Adverse Events (SAE) should be immediately reported to the Research Ethics Committee
