# Supplementary material for: High dose rifampin for 2 months vs standard dose rifampin for 4 months, to treat TB infection: Protocol of a 3-arm randomized trial (2R2)
Source: PLoS One. 2023 Feb 2;18(2):e0278087. doi: 10.1371/journal.pone.0278087 (PMC9894386; doi:10.1371/journal.pone.0278087)
Supplement: S6 File — (PDF) [file pone.0278087.s007.pdf]

BỘ Y TẾ
HỘI ĐỒNG ĐẠO ĐỨC TRONG NGHIÊN CỨU Y SINH HỌC QUỐC GIA

CỘNG HÒA XÃ HỘI CHỦ NGHĨA VIỆT NAM
Độc lập - Tự do - Hạnh phúc

Số: 156 /CN-HĐĐĐ

Hà Nội, ngày 28 tháng 12 năm 2020

GIẤY CHỨNG NHẬN

Chấp thuận của Hội đồng đạo đức trong nghiên cứu y sinh học quốc gia

Căn cứ Quyết định số 1122/QĐ-BYT ngày 02/02/2018 của Bộ trưởng Bộ Y tế về việc thành lập Hội đồng đạo đức trong nghiên cứu y sinh học quốc gia nhiệm kỳ 2018 - 2023;

Căn cứ Quyết định số 1155/QĐ-BYT ngày 02/02/2018 của Bộ trưởng Bộ Y tế về việc ban hành Quy chế tổ chức và hoạt động Hội đồng đạo đức trong nghiên cứu y sinh học quốc gia nhiệm kỳ 2018 - 2023;

Căn cứ Biên bản số 51/BB-HĐĐĐ ngày 04/11/2020 của Hội đồng đạo đức trong nghiên cứu y sinh học quốc gia;

Hội đồng đạo đức trong nghiên cứu y sinh học quốc gia chấp thuận về khía cạnh đạo đức trong nghiên cứu đối với đề cương nghiên cứu:

1. Tên nghiên cứu: 2R2: Thử nghiệm lâm sàng ngẫu nhiên phác đồ sử dụng 2 tháng Rifampin liều cao so với 4 tháng Rifampin liều chuẩn trong điều trị lao tiềm ẩn.
2. Nghiên cứu viên chính: PGS. TS. Nguyễn Viết Nhung, TS. BS Dick Menzies, PGS.TS Gregory James Fox.
3. Cơ sở thử lâm sàng: Bệnh viện Phổi Trung ương.
4. Địa điểm triển khai: Bệnh viện Phổi Hà Nội, Trung tâm y tế quận Hai Bà Trưng- Hà Nội, Trung tâm y tế quận Nam Từ Liêm – Hà Nội, Trung tâm điều trị Bệnh hô hấp Phổi Việt.
5. Đối tượng nghiên cứu: Người lớn và trẻ em từ 10 tuổi trở lên, có cân nặng từ 25 kg trở lên, có bằng chứng nhiễm lao tiềm ẩn (dương tính xét nghiệm da tuberculin hoặc xét nghiệm IGRA dương tính), đáp ứng tất cả tiêu chuẩn lựa chọn và không có bất kỳ tiêu chuẩn loại trừ nào theo đề cương nghiên cứu.
6. Số lượng đối tượng dự kiến: 450 bệnh nhân.
7. Thời gian thực hiện: 2020-2024.

Ngày chấp thuận: Ngày 28 tháng 12 năm 2020

Những thay đổi trong triển khai nghiên cứu sẽ phải được Hội đồng đạo đức trong nghiên cứu y sinh học quốc gia xem xét và chấp thuận, trừ trường hợp rõ ràng cần thiết thay đổi để loại trừ nguy cơ trực tiếp cho đối tượng.

Nghiên cứu viên chính phải báo cáo các trường hợp biến cố bất lợi, biến cố bất lợi nghiêm trọng cho Hội đồng đạo đức trong nghiên cứu y sinh học quốc gia theo đúng các quy định hiện hành.

Nơi nhận:
- Cơ sở thử thuốc trên lâm sàng;
- Nghiên cứu viên chính;
- Lưu: VP HĐ.

CHỦ TỊCH HỘI ĐỒNG

BỘ Y TẾ HỘI ĐỒNG ĐẠO ĐỨC TRONG NGHIÊN CỨU Y SINH HỌC QUỐC GIA NHIỆM KỲ 2018 - 2023

Trương Việt Dũng
