# Supplementary material for: High dose rifampin for 2 months vs standard dose rifampin for 4 months, to treat TB infection: Protocol of a 3-arm randomized trial (2R2)
Source: PLoS One. 2023 Feb 2;18(2):e0278087. doi: 10.1371/journal.pone.0278087 (PMC9894386; doi:10.1371/journal.pone.0278087)
Supplement: S7 File — (PDF) [file pone.0278087.s008.pdf]

BỘ Y TẾ
BỆNH VIỆN PHỔI TRUNG ƯƠNG
HỘI ĐỒNG ĐẠO ĐỨC

Số: ...07..../.20./ CT-HĐĐĐ
V/v: Chấp thuận triển khai TNLS

CỘNG HÒA XÃ HỘI CHỦ NGHĨA VIỆT NAM
Độc lập – Tự do – Hạnh phúc

Hà Nội, ngày ..23...... tháng ...4. năm 2020

CHẤP THUẬN TRIỂN KHAI THỬ NGHIỆM LÂM SÀNG

Căn cứ Quyết định số 527/QĐ-BVPTƯ ngày 23 tháng 5 năm 2019 của Giám đốc Bệnh viện Phổi Trung ương về việc Thành lập Hội đồng Đạo đức.

Căn cứ vào Quy chế tổ chức và hoạt động của Hội đồng Đạo đức, Bệnh viện Phổi Trung ương.

Căn cứ biên bản họp ngày 26/02/2020 của Hội đồng Khoa học, Hội đồng Đạo đức, Bệnh viện Phổi Trung ương và bản chỉnh sửa theo các ý kiến góp ý của hai Hội đồng.

Hội đồng Đạo đức, Bệnh viện Phổi Trung ương chấp thuận về các khía cạnh khoa học và đạo đức trong nghiên cứu đối với đề tài nghiên cứu sau:

1. Tên nghiên cứu: **$2R^2$: Thử nghiệm lâm sàng ngẫu nhiên phác đồ sử dụng 2 tháng Rifampin liều cao so với 4 tháng rifampicin liều chuẩn trong điều trị lao tiềm ẩn.**
2. Mã số: 547/2020/NCKH
3. Chủ nhiệm đề tài: PGS.TS. Nguyễn Viết Nhung, GS. Dick Menzies, PGS. Greg Fox.
4. Thời gian dự kiến nghiên cứu: từ tháng 4/2020 đến tháng 6/2023

Chủ nhiệm đề tài và nhà tài trợ phải tuân thủ theo đúng Hướng dẫn thực hành lâm sàng tốt (GCP) và nội dung đề cương nghiên cứu đã được phê duyệt, đảm bảo an toàn tuyệt đối cho đối tượng tham gia nghiên cứu.

Báo cáo cho Hội đồng Đạo đức, Bệnh viện Phổi Trung ương những thay đổi trong thời gian triển khai nghiên cứu, các trường hợp biến cố ngoại ý không mong muốn và tiến độ thực hiện đề tài theo quy định.

PHÓ GIÁM ĐỐC BỆNH VIỆN

BỘ Y TẾ BỆNH VIỆN PHỔI TRUNG ƯƠNG

Đinh Văn Lượng

CHỦ TỊCH HỘI ĐỒNG ĐẠO ĐỨC

Nguyễn Văn Hưng

Scanned with CamScanner
