# Supplementary material for: High dose rifampin for 2 months vs standard dose rifampin for 4 months, to treat TB infection: Protocol of a 3-arm randomized trial (2R2)
Source: PLoS One. 2023 Feb 2;18(2):e0278087. doi: 10.1371/journal.pone.0278087 (PMC9894386; doi:10.1371/journal.pone.0278087)
Supplement: S8 File — (DOCX) [file pone.0278087.s009.docx]

Inclusivity in global research

PLOS’ policy on inclusivity in global research aims to improve transparency in the reporting of research performed outside of researchers’ own country or community and ensures that PLOS publications reporting global research adhere to high standards for research ethics and authorship. Authors of relevant research articles may be asked to complete the questionnaire below, which outlines ethical, cultural, and scientific considerations specific to inclusivity in global research. This questionnaire may be requested when researchers have travelled to a different country to conduct research, if research uses samples collected in another country, research with Indigenous populations or their lands, or if research is on cultural artefacts. Researchers travelling to another country solely to use laboratory equipment will not normally be required to complete the questionnaire. However, the questionnaire can be requested at the journal’s discretion for any submission – if you have been requested to complete this questionnaire by the PLOS journal you submitted to, please do so.

Please complete the questionnaire below and include this as a Supporting Information file with your manuscript. Note that if your paper is accepted for publication, this checklist will be published with your article in the supporting information files. Please ensure that you reference the checklist in the main body of your manuscript. We suggest adding a subsection ‘Inclusivity in global research’ to your Methods section and adding the following sentence: “Additional information regarding the ethical, cultural, and scientific considerations specific to inclusivity in global research is included in the Supporting Information (SX Checklist)”

The questions have been designed to be applicable to a wide range of study types, and there are subsections for both human subjects research and non-human subjects research. If any of the questions are not relevant to your research please mark them as “N/A” as appropriate.

**Ethical considerations, permits and authorship**

*This section is applicable to all research types.*

Provide details as to who granted permissions and/or consent for the study to take place in the Methods section of your manuscript. This should include the names of **all** ethics boards, governmental organizations, community leaders or other bodies that provided approval for the study. If individuals provided approval refer to these people by their role or title but do not list their name(s).

First referred to on page 20, reported with all required information in Table 4, on page 26.

If there were any deviations from the study protocol after approval was obtained please provide details of these changes in the Methods section of your manuscript.
Did this study involve local collaborators that are residents of the country where the research was conducted or members of the community studied? If you do not have any authors from said communities, please provide an explanation for this below.

This work is conducted by a group of researchers, professors and physicians, which include members from each of the countries in which the project takes place. The same multicenter team has been collaborating in clinical trials in TB prevention and treatment over more than a decade. In particular, the following collaborators are from Indonesia: Prof Rovina Ruslami and Dr Lika Apriani. Both live in Bandung, Indonesia, were they teach at the UNPAD. They have conducted several clinical trials and epidemiology studies in Bandung, Indonesia, independently or as part of the collaboration with McGill. Over the past 10 years, both have come to McGill University, Montreal, for research training courses, as have other members of their TB research group (systematic reviews, and TB research methods). Prof Ruslami is the Head of the Pharmacokinetic Lab that will conduct the PK analyses for the entire 2R^2^ research study, meaning samples are shipped from Canada and Vietnam to her lab at UNPAD, in Bandung. A trainee from Indonesia (Dr Fajri Gafar) will undertake Post-Doc fellowship training under the co-supervision of Prof’s Menzies and Ruslami.

Dr. Thu Anh Nguyen and Dr. Viet Nhung Nguyen both live and work in Ha Noi, Vietnam. Dr Viet Nhung Nguyen works at the National TB Program, managing the TB program in Vietnam. Dr. Thu Anh Nguyen works at the Woolcock Institute in Ha Noi, Vietnam, conducting several clinical trials within Vietnam.

Amendments to the first version of the protocol were submitted to the all etchic committees which had approved the first version. The protocol in supporting material is the last approved version. There was no protocol deviation nor new amendments after that approval.

Everyone listed as an author should meet PLOS’ criteria for authorship and all individuals who meet these criteria should be included in the author byline, rather than the acknowledgements. Authorship criteria is based on the International Committee of Medical Journal Editors (ICMJE) Uniform Requirements for Manuscripts Submitted to Biomedical Journals - for further information please see here: <https://journals.plos.org/plosone/s/authorship>.

**Human subjects research (e.g. health research, medical research, cross-cultural psychology)**

Did you obtain written informed consent from a representative of the local community or region before the research took place? How did you establish who speaks for the community? Details of written informed consent obtained from study participants should be reported separately in the Methods section of your manuscript.

How did members of the local community provide input on the aims of the research investigation, its methodology, and its anticipated outcome(s)?

In both Indonesia and Vietnam, local investigators and study coordinators met multiple times with health care providers working in primary health level and in TB clinics. Also, meetings were held with local stakeholders, in particular with TB program representatives. In Indonesia: meetings were held in Bandung, West Java, with Primary Health Centers (Puskesmas) and Bandung Municipal Health Office (TB program) – these are key local stakeholders and are responsible for providing primary health care, and TB services to the local population.

In Vietnam, meeting were held with the National TB Programme director, and his team, as well as the National TB Scientific and Ethic Committee at the National Lung Hospital to seek approval for the study, following which meetings were held with representatives of study sites; as well as meetings with the Ethic Committee at the Ministry of Health for ethic and scientific approval for clinical trials as part of the local authority requirement.

When engaging with the local community, how did you ensure that the informed consent documents and other materials could be understood by local stakeholders?

In Indonesia: all meetings with the local community were held in Bahasa Indonesia, by the site invetigators and coordiantors. An informed consent form (ICF) and a protocol summary, for the study, were developed in Bahasa Indonesia by Bandung’s research team. These documents were made according to the forms from the local Ethics committee.

In Vietnam: ICF and study materials (including all Case Report Forms- CRFs) were translated into Vietnamese and submitted to local authorities in advance of the formal meetings. The Vietnam co-authors organized a meeting with representatives of TB patient group in Hanoi to present the study protocol, CRFs and ICF, and obtained their comments for finalization. Meetings were also held to explain and discuss the study with doctors and nurses at study sites, as well as managers of TB program activities, and the Ministry of Health which is in charge of approval and monitoring all clinical trials. Comments on study protocol, CRFs and forms, implementation plan as well as allowance rate for study participants were provided at the meeting and were addressed fully in order for the study protocol to be approved. All meetings mentioned above were held by Vietnamese co-authors, in Vietnamese.

Will the findings of the research be made available in an understandable format to stakeholders in the community where the study was conducted (e.g. via a presentation, summary report, copies of publications, etc.)? Please provide details of how this will be achieved.

In Bandung, for a past trial conducted by the same investigators, seminars were given by study PI (Dick Menzies) and site PI (Rovina Ruslami) at National conferences within Indonesia, meetings with TB program, meetings with Bandung Municipal Health Office and Primary Health Centers (Puskesmas). We plan similar activities when this trial is completed.

In Vietnam, periodic reports are prepared in Vietnamese and presented to study sites as well as submitted to the National TB Program. Meetings with the local stakeholders (including National TB Program as well as clinic staff, will be held when the trial is completed to present the results.

**Non-human subjects research using specimens/ animals collected as part of the study, or those housed in archival collections. Examples include archaeology, paleontology, botany and zoology.**

Did the permission you obtained from a local authority to perform the study include an agreement on access to outputs and benefit sharing? This may include procedures to enable fair distribution of the benefits and resources arising from the research performed. Please include any details of Prior Informed Consent and Benefit Sharing Agreements obtained. These may be required by field-specific regulations, for example the Convention on Biological Diversity (CBD) and the associated Nagoya Protocol.

Not applicable

If the material used in your study was imported, please A) provide the year it was imported and B) indicate whether permits were obtained to import/export the materials used, C) provide details of any permits obtained. If this information is not available, please indicate this.

Not applicable

If you used archival specimens, please state how the material used in your study was acquired by the institute it is held in and provide details of any permits obtained for the original excavations/ sample collection. If this information is not available, please indicate this.

Not applicable

How was the potential cultural significance of the materials collected in your study to local communities considered in your research design? Were Indigenous peoples and/or local researchers and institutions involved with archaeological excavations / collection of specimens? If so, please provide a description of their involvement.

Not applicable

If your manuscript includes photographs of human remains please indicate whether authors obtained permission from descendants or affiliated cultural communities to do so.

Not applicable
