# Supplementary material for: High dose rifampin for 2 months vs standard dose rifampin for 4 months, to treat TB infection: Protocol of a 3-arm randomized trial (2R2)
Source: PLoS One. 2023 Feb 2;18(2):e0278087. doi: 10.1371/journal.pone.0278087 (PMC9894386; doi:10.1371/journal.pone.0278087)
Supplement: S3 File — (PDF) [file pone.0278087.s004.pdf]

Centre universitaire de santé McGill | McGill University Health Centre

2019-07-17 revised 2019-08-07

Dr. Dick Menzies
Respiratory Medicine
MUHC - Montreal Children's Hospital
1001 boul. Decarie
Montreal, QC H4A 3J1

email: chantal.valiquette@mail.mcgill.ca

RE: Final REB Approval of a New Research Project
2R2: Higher dose Rifampin for 2 months vs Standard dose Rifampin for Latent TB: a 3-arm randomized trial **(2R2 / 2019-5360)**

MUHC REB Co-Chair for the PED panel: M. Vincent Lajoie

Dear Dr. Menzies,

Thank you for submitting your responses and corrections for the research project indicated above, as requested by the McGill University Health Centre (MUHC) Research Ethics Board (REB).

The MUHC REB, more precisely its pediatric (PED) panel provided conditional approval for the research project at its full board meeting of 2019-04-15.

On 2019-07-17, a delegated review of your responses and corrections was provided by member(s) of the MUHC REB. The research project was found to meet scientific and ethical standards for conduct at the MUHC.

The following documents were approved or acknowledged by the MUHC REB:

- Initial Submission Form (F11-40433)
- REB Conditions & PI Responses Form(s) (F20-43494, 44835, & 46473)
- Protocol 2R2, June 7, 2019_REBapproved
- Health Canada (NOL228350_approval), Date: 2019-07-04
- Document(s) approved by the REB:
- (2R2 card for end of study_En_Fr_REBapproved.docx), Date:2019-05-24, Version: 1
- 2R2_ICF_Eng_REBapproved, Date: 2019-05-23, version 1
- 2R2_ICF_FR_REBapproved, Date: 2019-07-04, version 1

This will be reported to the MUHC REB and will be entered accordingly into the minutes of the next PED meeting. Please be advised that you may only initiate the study after all required reviews and decisions are received and documented and you have received the MUHC authorization letter.

<u>The approval of the research project is valid until 2020-07-16.</u>

All research involving human subjects requires review at recurring intervals. To comply with the regulation for continuing review of at least once per year, it is the responsibility of the investigator to submit an *Annual Renewal Submission Form* (F9) to the REB prior to expiry. Please be advised that should be protocol reach its expiry before a Continuing review has been submitted, the data collected after the expiry date may not be considered valid. However, should the research conclude for any reason prior to approval expiry, you are required to submit a *Completion (End of Study) Report* (F10) to the board once the data analysis is complete to give an account of the study findings and publication status.

Furthermore, should any revision to the project or other development occur prior to the next continuing review, you must advise the REB without delay. Regulation does not permit initiation of a proposed study modification prior to its approval by the REB.

The MUHC REB is registered and works under the published guidelines of the *Tri-Council Policy Statement 2*, in compliance with the *Plan d'action ministériel en éthique de la recherche et en intégrité scientifique* (MSSS, 1998) and the *Food and Drugs Act* (2001.06.07), acting in conformity with standards set forth in the (US) *Code of Federal Regulations* governing human subjects research and functioning in a manner consistent with internationally accepted principles of good clinical practice.

CLINICAL TRIALS: We wish to advise you that the MUHC REB "working procedures" completely satisfies the requirements for Research Ethics Board Attestation (REBA) as stipulated by Health Canada. Neuro and MUHC research that is subject to US Federal Wide Assurance is conducted under FWA00000840.

We trust this will prove satisfactory to you. Thank you for your consideration in this matter.

Best Regards,

Ms. Elizabeth Craven, Coordinator, MUHC REB
MUHC REB Coordinator
for MUHC Co-chair mentioned above
